# Psychological mediators of the associations between adverse and benevolent childhood experiences and posttraumatic stress disorder (PTSD) and complex PTSD: A systematic review and meta‐analysis

**DOI:** 10.1002/jts.70098

**Published:** 2026-06-25

**Authors:** Sarah Louise Guthrie, Thanos Karatzias, Mark Shevlin, Anna Bak‐Klimek, Amanda Woodrow

**Affiliations:** ^1^ Edinburgh Napier University, School of Health & Social Care Edinburgh UK; ^2^ Ulster University, School of Psychology Derry UK; ^3^ NHS Lothian Rivers Centre for Traumatic Stress Edinburgh Uk

## Abstract

Adverse and benevolent childhood experiences (ACEs and BCEs, respectively) are uniquely associated with posttraumatic stress disorder (PTSD) and complex PTSD (CPTSD); however, there is no systematic review on the mechanisms of these associations. This systematic review and meta‐analysis aimed to synthesize studies considering psychological mediators of the associations between ACEs/BCEs and PTSD/CPTSD. Databases (PsychINFO, PubMed, PTSDpubs, PsycARTICLES, and Web of Science) were searched from inception to August 2025 for peer reviewed studies of adult populations examining psychological mediators of the associations between ACEs or BCEs and PTSD or CPTSD. A total of 63 studies involving 31,540 participants met the inclusion criteria. Mediators were grouped into six categories: cognitive beliefs and appraisals (*n* = 33), emotion regulation strategies (*n* = 40), interpersonal relationships (*n* = 7), spiritual and religious beliefs (*n* = 2), attachment style (*n* = 5), and dissociation symptoms (*n* = 9), with mostly small effect sizes. Subcategory meta‐analysis revealed emotion regulation, β = .4, 95% CI [0.001, 0.78], and maladaptive coping strategies, β = .05, 95% CI [0.03, 0.07], significantly mediated the association between ACEs and PTSD. Risk of bias and quality of evidence were assessed. Overall, evidence was insufficient to determine how mediators function across pathways, and a paucity of literature is highlighted, considering BCEs and CPTSD.

Exposure to adverse childhood experiences (ACEs), defined in this review to encompass all types of childhood maltreatment, including physical, emotional, and household dysfunction domains, is a well‐established risk factor for a plethora of physical and mental health conditions across the lifespan (Crouch et al., 2018; Felitti et al., 1998; Merrick et al., 2017), including posttraumatic stress disorder (PTSD; LeardMann et al., 2010; Schalinski et al., 2016) and complex PTSD (CPTSD; Choi et al., 2021). CPTSD was introduced in the 11th revision of the version of *International Statistical Classification of Diseases and Related Health Problems* (*ICD‐11*; World Health Organization, 2019) as a sibling condition to PTSD. The former comprises three symptom clusters—reexperiencing, avoidance, and hypervigilance—whereas the latter includes these three core symptom clusters alongside three symptom clusters commonly referred to as *disturbances in self‐organization* (DSO) symptoms. These DSO clusters include affective dysregulation, negative self‐concept, and difficulties in relationships. Although either condition can result from exposure to any traumatic event, single traumatic events are more likely to be associated with PTSD, whereas childhood, ongoing, or prolonged trauma are more likely to be associated with CPTSD (e.g., Hyland et al., 2017).

Benevolent childhood experiences (BCEs), defined in this review to encompass positive childhood experiences across interpersonal and environmental domains reflecting safety and predictability, have been shown to be protective against ACEs in relation to various conditions, including PTSD, depression, anxiety, and suicidal ideation (Feiler et al., 2023; Hou et al., 2022; Narayan et al., 2023). In relation to CPTSD, Karatzias et al. (2020) found that higher levels of BCE exposure were directly related to lower DSO scores. This study also revealed that ACEs and BCEs were related yet latently distinct concepts, with the authors suggesting that BCEs may be able to offset some of the damaging effects of ACEs, particularly for CPTSD DSO symptoms. This is an important finding, as CPTSD remains a more functionally impairing condition for which current treatments may be less effective in comparison to PTSD (Karatzias et al., 2018).

Alongside acting as a protective buffer against PTSD and CPTSD, BCEs may facilitate the development of posttraumatic Growth (PTG) following trauma exposure. PTG is defined as a positive psychological transformation characterized by growth following adversity through the positive accommodation of trauma‐related information (Joseph & Liney, 2005), a specifically defined trauma outcome differential to the absence of posttraumatic stress symptoms (PTSS). Research has shown that BCEs significantly predict PTG, with this association partly mediated by self‐compassion and self‐esteem (Çağlar & Kaya, 2025). Mechanisms associated with PTG include resilience and adaptive coping (Campodonico et al., 2021), and although PTG is a specifically defined trauma outcome differential to the absence of PTSS, its unique association with BCEs demonstrates a theoretical mechanism for posttraumatic resilience and adaptation. This is also consistent with the *broaden and build theory of positive emotions*, which suggests positive emotions broaden cognitive and behavioral repertoires and build psychological resources, such as resilience and coping capacity (Fredrickson, 2004).

## Theoretical frameworks

Current models of trauma outcomes explain the maintenance of symptoms through the inclusion of core functional psychological mechanisms. One of the most influential models is the *cognitive model of PTSD* (Ehlers & Clark, 2000), which outlines that after trauma exposure, a series of psychological processes, including negative trauma appraisals, disturbances in autobiographical memory, and detrimental behavioral and cognitive coping strategies, is responsible for the maintenance of symptoms. Similarly, the *dual representation theory* (Brewin et al., 1996) outlines that some PTSD symptomatology, especially reexperiencing symptoms, is maintained through impaired memory systems. *Emotional processing theory* (Foa & Kozak, 1986) states that PTSD symptoms are maintained through unprocessed “fear structures” in memory. More recently, the *memory and identity theory of CPTSD* (Hyland et al., 2023) posits that prolonged trauma exposure creates vivid, sensation‐based trauma memories alongside negative perceptions of the self (e.g., worthlessness), which together maintain DSO symptoms (Hyland et al., 2025).

Models and frameworks of trauma outcomes emphasize that PTSS are maintained through psychological mechanisms. Further, such mechanisms are often influenced by childhood experiences. For example, ACEs are associated with an increased tendency to ruminate (Mansueto et al., 2021), disturbances in autobiographical memory (Brown et al., 2007), impaired cognitive control (Rahapsari & Levita, 2025), and maladaptive coping strategies (e.g., avoidance, lower use of problem‐focused strategies; Sheffler et al., 2019). Conversely, BCEs have demonstrated associations with adaptive mechanisms, such as better cognitive functioning and picture sequence memory (Logan & Lewis–de Los Angeles, 2025). ACEs and BCEs may, therefore, be uniquely predictive of PTSS by influencing mechanisms that may function either adaptively or maladaptively, making mediation studies important to consolidate.

## Research aims and rationale

This review aims to reveal which psychological mechanisms are relevant functional mediators in the associations between ACEs/BCEs and PTSD/CPTSD. By considering BCEs alongside ACEs, potential protective mechanisms and pathways can be investigated. Further, the consideration of both PTSD and DSO outcomes allows for the consideration of how ACEs and BCEs may be uniquely associated with differing trauma pathways. Finally, by revealing relevant mechanisms, this review aims to highlight potential targets for interventions and treatments and reveal gaps in the literature.

## METHODS

### Transparency and openness

This review was conducted following the Preferred Reporting Items for Systematic Reviews and Meta‐Analyses (PRISMA; Page et al., 2021) guidelines and the Transparency and Openness Promotion Guidelines (TOP; Nosek et al., 2015) with methodology preregistered (PROSPERO registration number: CRD42023439863). All supplementary materials and data used are publicly available at the Open Science Framework (https://osf.io/a7bj4/?view_only=b3616f0a6854474f927f65a0d039f607).

### Inclusion criteria

For inclusion in this review, articles had to have used a measure of childhood experiences that can be defined as adverse or benevolent, a validated measure of PTSD and/or CPTSD diagnostic criteria, and a measure of psychological mechanisms that may mediate the associations between ACEs/BCEs and PTSD/CPTSD. Studies were eligible if they were a prospective cohort, cross‐sectional, or case‐control study that assessed the previously described associations using a mediation analysis or other suitable modeling approach. Reporting either the completely standardized indirect effect (CSIE) directly or sufficient information to convert indirect effects to the CSIE was not an inclusion criterion, as the feasibility of CSIE conversion could only be determined following full data extraction. Studies for which effect sizes could not be converted were included in the narrative mapping of the literature.

Due to heterogeneity in mediation modeling approaches, non–peer reviewed works were excluded to enhance methodological and reporting consistency. For homogeneity and to maintain developmental consistency, whereby mediation effects may differ substantially in children or adolescents, studies involving children or adolescents under 18 years of age were also excluded. Finally, articles that were not published in English were excluded.

### Search procedure

The PsycInfo, PubMed, PTSDpubs, PsycARTICLES, and Web of Science databases were searched using the following search terms in title and/or abstract: (PTSD or traumatic N1 stress or PTSS or CPTSD) AND (child* N2 experiences or ACEs or BCEs or child* N2 advers* or child* N2 benevolen* or child* N2 maltreat* or child* N2 trauma*) AND (mediat* or associat* or relat*). The same search terms were used in the following journals: *Trauma*, *Psychological Trauma: Theory, Research, Practice, and Policy, Journal of Traumatic Stress*, *Child and Adolescent Mental Health*, and *Childhood*. The search captured studies published up to August 2025 (see Figure 1 for a PRISMA diagram; see Supplementary Materials 1 for the data extraction procedure)

**FIGURE 1:**
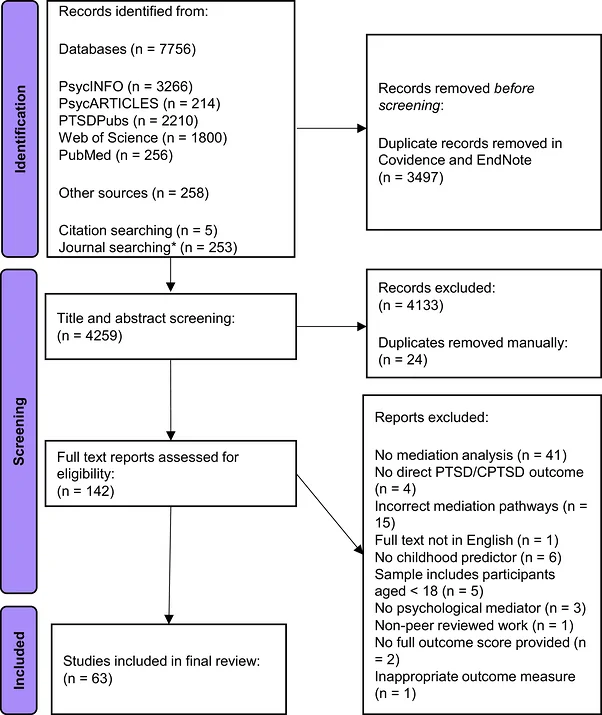
PRISMA flow diagram *Note*: PTSD = posttraumatic stress disorder; CPTSD = complex posttraumatic stress disorder.

### Mediator categorisation & meta‐analysis approach

Two researchers classified mediator variables into psychological categories. In cases of disagreement between these reviewers, detailed consideration of the measure used and additional research, where necessary, were conducted until the reviewers reached an agreement. Following mediator category assignments, data extraction and CSIE calculations were performed; as all standardized CSIE calculations were objective and formula‐based, they were not subject to coding disagreement. Total instrument scores were used in the first instance, with available subscale results included only when total scores were unavailable. This approach allowed for the visualization of effect sizes per category.

Next, subsets of mediators that contained sufficient conceptual and operational similarity across at least three studies were aggregated for meta‐analysis. For inclusion in a subset, mediators had to be of the same type and definition (e.g., “rumination”), considered within the same pathway (e.g., ACEs > PTSD), and be measured using the same instrument or a comparable measurement tool. This dual‐level categorization reflects the heterogeneity in the literature and allowed this review to capture both the broad range and, where possible, meaningful aggregation of indirect effects. Due to high heterogeneity, a limited number of mediator groupings were analyzed. Both Bayesian and frequentist methods were applied, as Bayesian models can accommodate asymmetrical confidence intervals (CI) and skew (Juárez & Steel, 2010), with asymmetrical confidence intervals common with indirect effects (Preacher & Hayes, 2008), and can allow for the consideration of prior knowledge to estimate the degree of heterogeneity between studies (useful if the number of included studies is low; Agapova et al., 2014; R. Harvey et al., 2020).

Meta‐analysis was performed in R (Version 4.5.2; R Core Team, 2020) using the following packages: *brms* (Bürkner, 2017), *meta* (Balduzzi et al., 2019), *demetar* (Harrer et al., 2019), and *metafor* (Viechtbauer, 2010). Graphics were created using *ggplot2* (Wickham, 2016). Fixed effects Bayesian models were used due to the low numbers of CSIEs per mediator group, creating model instability in random‐effects models. Weakly informative priors (normal 0, 1) were specified following stability recommendations for small datasets (Lemoine, 2019) and to avoid overfitting (Semochkina & Walsh, 2025). Gaussian models were used after Student's *t* was found to be incompatible, likely due to small amounts of data per group (Hernandez‐Velasco et al., 2023). The fixed‐model Gaussian approach assumes symmetry, which was appropriate for our dataset; however, it assumes a single true effect and, thus, disregards heterogeneity (Röver, 2020). Fixed‐ and random‐effects frequentist models (restricted maximum likelihood; REML) were also employed to enhance robustness and interpretation and assess heterogeneity, as well as for sensitivity analysis.

### Appraisal of evidence

To assess risk of bias, an adapted version of the Agency for Healthcare Research and Quality (AHRQ) assessment tool (Viswanathan et al., 2017; Woodrow, 2021) was used. To assess the overall quality of evidence, the Grading of Recommendations, Assessment, Development, and Evaluations (GRADE; Guyatt et al., 2008) system was employed. See Supplementary Materials 1 and Supplementary Table S1 for descriptions of each tool and procedure.

## RESULTS

### Search results

The database search yielded 7,756 articles (see Figure 1), 63 of which met the inclusion criteria. Of these 63 studies, 53 either directly provided CSIEs and confidence intervals or provided information to allow them to be calculated (studies requiring calculations: *n* = 24). It was not possible to calculate CSIEs for the remaining 10 papers, and these studies were not considered in the GRADE assessments. See Supplementary Materials 2 for demographic characteristics, specifically Supplementary Table S2, and Supplementary Materials 3 for full calculation details by study.

### Mediator categories

Mediators were grouped into six categories (see Table 1 for the grouping strategy). Only two categories—cognitive beliefs and appraisals and emotion regulation strategies— contained more than 10 effects; thus, the remaining category results are reported in Supplementary Materials 2. For the considered predictors, outcomes, mediators, and mediator grouped categories by study, see Supplementary Table S3.

**TABLE 1:** Definitions used in study categorizations

Category	Number of effects	Definition
Cognitive beliefs & appraisals	33	Adaptive and maladaptive cognitive interpretations, belief systems, and self‐related cognitive processes concerning understanding of the self, others, and traumatic experiences
Emotion regulation strategies	40	Adaptive and maladaptive processes involved in modulating emotional response, including regulatory capacity, coping strategies, and cognitive or attentional regulation of affect
Interpersonal relationships	7	Perceived and structural interpersonal resources, including the availability, quality, and acknowledgement of support
Spiritual and religious beliefs	2	Religious and spiritual belief systems that shape meaning‐making and response to adversity
Attachment style	5	Individual attachment style and insecurities that shape interpersonal relationships and emotional response to others
Dissociation symptoms	9	Symptoms indicative of dissociative experiences, including disruptions to consciousness, memory, and identity

### Quality assessment

As assessed using the AHRQ for risk of bias tool, most studies received a “moderate” risk of bias rating (*n* = 33, 52.4%), and 30 studies received a “low” risk of bias rating (47.6%). No studies received a “high” risk of bias rating. See Supplementary Table S4 for the AHRQ risk of bias assessment for each individual study.

Each category and meta‐analysis subgroup was assessed using the GRADE criteria. All outcomes originated from nonrandomized control trial studies (the majority were cross‐sectional studies) and, thus, were rated “low” before further grading (Balshem et al., 2011). Each category was downgraded at least once and, thus, received a very low‐quality rating. See Supplementary Table S5 and S6, with corresponding text, in Supplementary Materials 2 for full GRADE assessment by category.

### Category results

#### Cognitive beliefs and appraisals

Four studies in this category considered CPTSD, whereas 21 considered PTSD. Watts et al (2025) found the largest effect size, reporting that traumatic appraisals mediated the association between childhood psychological trauma and CPTSD, CSIE = 0.7, 95% CI [0.60, 0.79]. For PTSD outcomes, Mitchell et al. (2018) found the largest effect size, reporting that trauma appraisals mediated the association between childhood trauma and PTSD, CSIE = 0.30 [0.18, 0.46]. Additional support comes from the only longitudinal study in this review (Menke et al., 2018), which showed smaller but consistent effects of maltreatment‐specific shame as a mediator at 6‐month, CSIE = 0.19 [0.10, 0.31]; 12‐month, CSIE = 0.19 [0.10, 0.31]; 15‐month, CSIE = 0.17 [0.07, 0.29]; and 18‐month follow‐ups, CSIE = 0.09 [0.07, 0.29]. See Figure 2 for forest plots and Supplementary Table S7 for full results.

**FIGURE 2:**
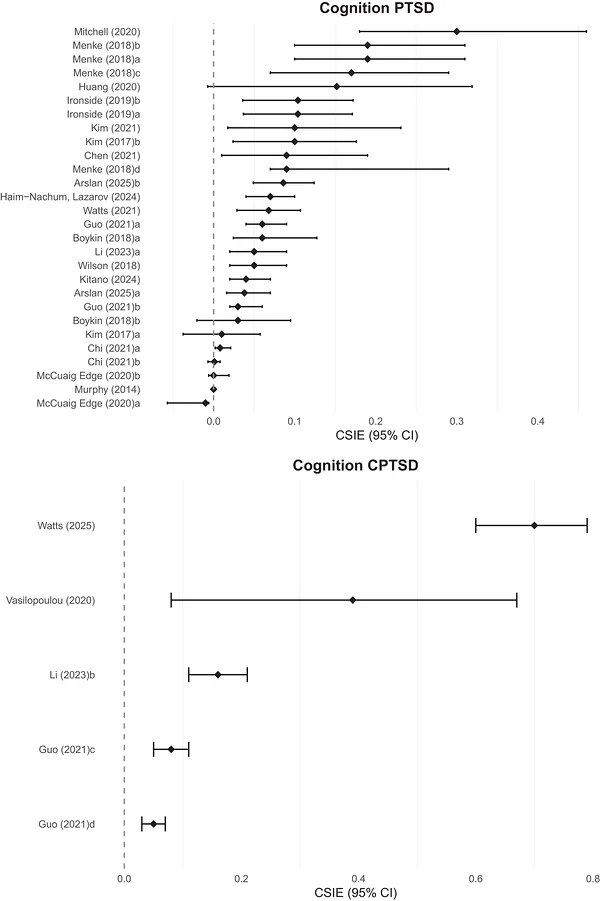
Forest plot for cognitive beliefs and appraisals mediators between adverse childhood experiences (ACEs) and (A) posttraumatic stress disorder (PTSD) and (B) complex PTSD (CPTSD) *Note*: CSIE = completely standardized indirect effect; CI = confidence interval.

#### Emotion regulation strategies

Four studies in this category considered CPTSD, whereas 23 considered PTSD. Feiler et al. (2023), the only included study to consider both adverse and benevolent childhood experiences, reported the largest effect size in this category. The findings demonstrated that emotion dysregulation mediated the associations between both ACEs and PTSD, CSIE = 1.49 [1.20, 1.81], and BCEs and PTSD, CSIE = −2.31 [−1.92, −2.73]. Among studies that considered CPTSD as an outcome, the largest effect size was found by Békés et al. (2023), who found that shame mediated the associations between childhood trauma and both PTSD, CSIE = 0.41 [0.09, 0.82], and DSO symptoms, CSIE = 0.42 [0.13, 0.76]. See Figure 3 for forest plots and Supplementary Table S8 for full results.

**FIGURE 3:**
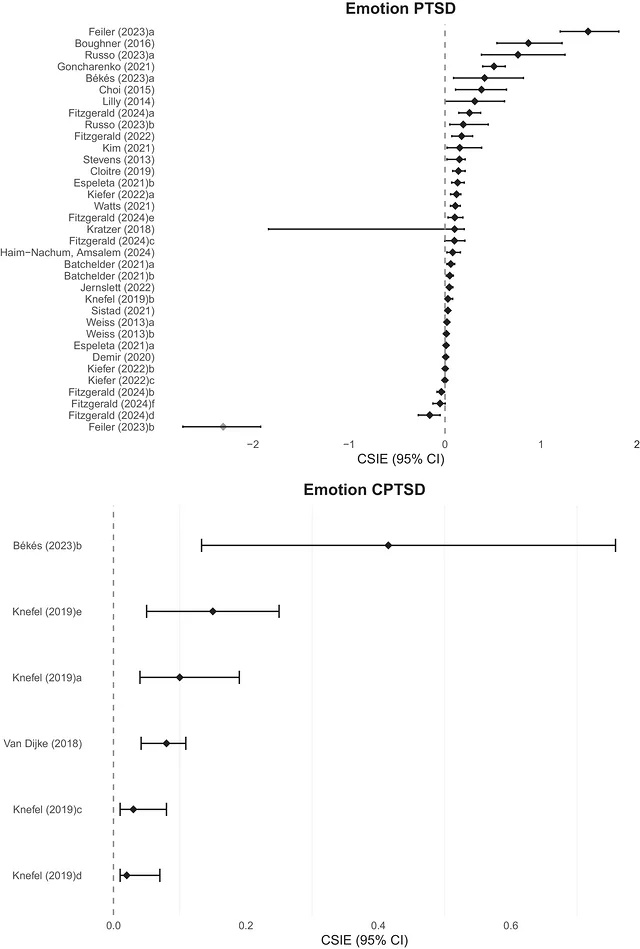
Forest plot for emotion regulation strategies mediators between adverse childhood experiences and (A) posttraumatic stress disorder (PTSD) and (B) complex PTSD (CPTSD) *Note*: Feiler 2 (Panel A) was the only study that reported an effect size considering benevolent childhood experiences (BCEs) as a predictor; it is represented by a blue marker. CSIE = completely standardized indirect effect; CI = confidence interval.

### Meta‐analysis

Four mediator subgroups were aggregated for meta‐analysis, with each conceptualized consistently in the ACEs‐to‐PTSD pathway. Six effects considered emotion regulation, as measured using the Difficulties with Emotion Regulation Scale (DERS; Gratz & Roemer, 2004) or its subscales or short‐form version. Four effects considered resilience, as measured using the Connor–Davidson Resilience Scale (CDRS; Connor & Davidson, 2003) or its short‐form version. Five effects considered maladaptive coping strategies, as measured by The Brief COPE (or subscales; Carver, 1997). Nine effects considered mindfulness, as measured using the Five Facet Mindfulness Questionnaire (FFMQ; Baer et al., 2006) or its subscales or short‐form version or the Freiburg Mindfulness Inventory (FMI; Walach et al., 2006). When measurement tools differed, and when multiple effects originated from the same study, sensitivity analyses were run to ensure stability (see Supplementary Tables S9–S12 for full details).

Due to high heterogeneity across mediation results, it was not possible to use standard effect sizes (e.g., Cohen's *d*, Pearson's *r*). The CSIE was utilized to allow for standardized comparisons of indirect effects across diverse studies and methodologies. Consequently, interpretations focus on the magnitude of effect, with confidence intervals provided for consideration of precision.

#### Meta‐analysis results

Only emotion regulation and maladaptive coping strategies were significant; see Supplementary Materials 2 for the results for resilience and mindfulness.

##### Emotion regulation

This category contained seven effects from six studies (total sample size: *N* = 2,504). A Bayesian fixed‐effects meta‐analysis with weakly informative priors revealed a small but statistically meaningful positive effect, *M*_posterior_ = 0.03 (*SD* = 0.01), 95% CI [0.02, 0.04], with strong convergence, $\hat{R}$ (R‐hat convergence diagnostic for Markov Chain Monte Carlo [MCMC] chain simulations) = 1.00, and effective sample sizes for bulk and tail estimates were high (bulk = 983, tail = 891), demonstrating stable estimates (see Supplementary Figure S1 for posterior distribution). Results from a frequentist fixed‐effects meta‐analysis aligned closely with the Bayesian model and revealed a small but significant positive effect, β = 0.03 (*SE* = 0.01), 95% CI [0.02, 0.04], *p* < .001; however, substantial heterogeneity was observed, *I* ^2^ = 95.13%, *Q*(6) = 123.24, *p* < .001.

A random‐effects model was employed using REML. The pooled effect size was larger, β = 0.39 (*SE* = 0.12, 95% CI [0.001, 0.78], *p* = .05, but it also had much wider confidence intervals and a weaker *p* value. This shows increased uncertainty due to high levels of between‐study variance, *T* ^2^ = 0.27, *I* ^2^ = 99.83%, and true effects, therefore, may vary across studies. Taken together, these findings demonstrate a small positive mediating effect with high levels of heterogeneity across studies (see Figure 4). Sensitivity checks were run twice and did not influence the findings (see Supplementary Table S13 for full results).

**FIGURE 4:**
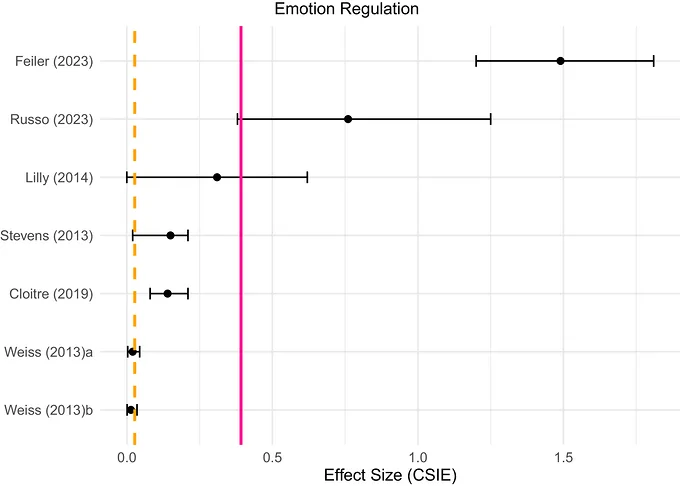
Forest plot for emotion regulation meta‐analysis *Note*: The solid pink line represents frequentist random effects, and the dashed orange line represents Bayesian fixed effects. Error bars represent 95% confidence intervals. CSIE = completely standardized indirect effect.

##### Maladaptive coping strategies

This category contained five effects from four studies (total sample size: *N* = 1,484). A Bayesian fixed‐effects meta‐analysis with weakly informative priors revealed a positive effect that was verging on moderate, *M*_posterior_ = 0.05 (*SD* = 0.01), 95% CI [0.03, 0.07], with strong convergence ($\hat{R}$ = 1.00), and effective sample sizes for bulk and tail estimates were high (bulk = 1,045, tail = 829), indicating stable posterior estimates (see Supplementary Figure S2 for posterior distribution). Results from a frequentist fixed‐effects meta‐analysis showed a smaller significant pooled effect size, β = 0.05 (*SE* = 0.01), 95 % CI [0.03, 0.07], *p* < .001. Although not significant, moderate heterogeneity was observed, *I* ^2^ = 46.55%, *Q*(4) = 7.48, *p* = .112. A random‐effects model was, therefore, conducted using REML and closely aligned with the other models, demonstrating a small, significant pooled effect, β = 0.05 (*SE* =  0.01), 95% CI [0.03, 0.07], *p* < .001. Heterogeneity was shown to be negligible and nonsignificant, *I* ^2^ = 0.23%, *Q*(4) = 7.48, *p* = .112, indicating consistency across studies (see Figure 5). Sensitivity checks were run twice and did not influence the findings (See Supplementary Table S14 for full results).

**FIGURE 5:**
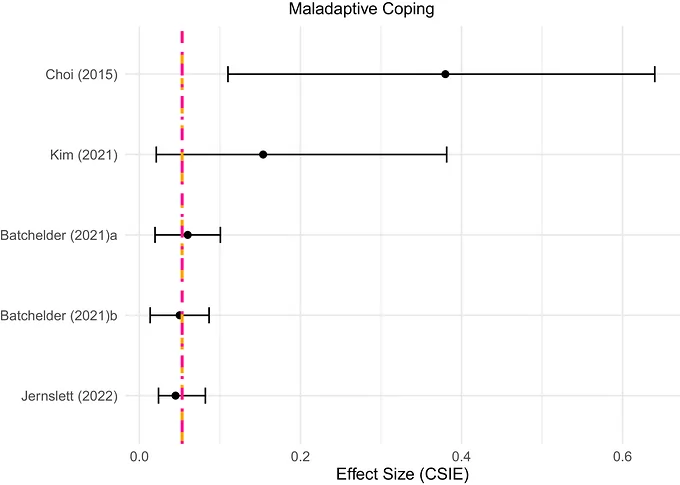
Forest plot for maladaptive coping meta‐analysis *Note*: The pink dot–dash like represents frequentist random effects, and the orange dashed line represents Bayesian fixed effects. Error bars represent 95% confidence intervals. CSIE = completely standardized indirect effect.

## DISCUSSION

This review examined psychological mechanisms in the associations between ACEs and BCEs and the presence of PTSD and CPTSD. Across 63 studies, mediators converged into the following categories: cognitive beliefs and appraisals, emotion regulation strategies, interpersonal relationships, spiritual and religious beliefs, attachment style, and dissociation symptoms. Cognitive beliefs and appraisals and emotion regulation difficulties were the largest categories with the most consistent evidence of mediation effects in the association between ACEs and PTSD, aligning with existing literature (Cloitre et al., 2019). Findings suggest that ACEs may shape trauma‐related cognitions and emotional regulatory capacity that influence vulnerability to PTSD and CPTSD.

Evidence regarding the protective effects of BCEs was limited. The only study that considered BCEs found a moderately significant mediating effect of emotion regulation in the association between BCEs and PTSD, alongside a moderating effect of BCEs on the association between ACEs and emotion dysregulation in relation to PTSD (Feiler et al., 2023).

The meta‐analysis further supported the roles of emotion regulation and maladaptive coping strategies as mediators in the association between ACEs and PTSD, a finding that is also consistent with existing literature (Ehlers & Clark, 2000). Conversely, resilience and mindfulness demonstrated no consistent mediating effects, contrasting with literature suggesting the protective role of these constructs (Ortiz & Sibinga, 2017; Goldenson et al., 2020) and reflecting variability between studies. Overall, the quality of evidence was low, as illustrated by the risk of bias and GRADE assessments; thus, the findings should be interpreted cautiously.

Most studies in this review were conducted in high‐income Western countries, with several using student populations, which may limit the generalizability of the findings. Demographic characteristics were varied, with many samples relatively homogeneous (see Supplementary Table S2 for full demographic data). Individuals from minority backgrounds, specifically low‐income and racial/ethnic minority groups, are more likely to experience ACEs (Mersky et al., 2021) while experiencing inequality regarding access to positive experiences and safety resources (Crouch et al., 2021); thus, the mechanisms identified in this review may operate differently across contexts.

The findings of this review align with posttraumatic models emphasizing the role of cognition and emotional mechanisms. For example, the cognitive model of PTSD (Ehlers & Clark, 2000) states that PTSD is maintained through negative trauma appraisals, memory disturbance, and maladaptive coping strategies. The memory and identity theory of CPTSD (Hyland et al., 2023) emphasizes the role of dissociation, emotion regulation, and negative identities in CPTSD. The findings also suggest that early developmental experiences may impact trauma outcomes through the functionality of specific psychological mechanisms. ACEs have been associated with emotion regulation difficulties, lower executive function, and reduced cognitive flexibility (Cole & Diaz, 2024), mechanisms that may shape how traumatic experiences are interpreted and integrated. Conversely, BCEs may support adaptive processes, which potentially buffer the impact of adversity (Feiler et al., 2023; Karatzias et al., 2020), although research here remains limited. Taken together, these findings correspond with empirical and theoretical frameworks of childhood development and, more specifically, that early childhood is a sensitive developmental window in which adversity can negatively impact the developing brain (Bick & Nelson, 2016). Disruptions during this period may contribute to vulnerability following a traumatic experience.

Emerging research suggests that BCEs may buffer against the effects of adversity. The *PTSD–positive memory model* (Contractor et al., 2022) suggests that activating positive autobiographical memories may reduce PTSD symptoms, with interventions showing initial success (Greene et al., 2024). Such approaches may provide justification for integrating BCEs into treatment. Ongoing research may also provide evidence for preventative measures, especially for children most at risk for experiencing adversity (Sege & Harper Browne, 2017).

The mediators identified in this review also support established treatments for PTSD and CPTSD that target cognitive and emotional processes. *Trauma‐focused cognitive behavioral therapy* (TF‐CBT; de Arellano et al., 2014) addresses aspects of negative cognitive appraisals and maladaptive coping techniques associated with trauma (A. G. Harvey et al., 2003). Similarly, *enhanced skills training in affective and interpersonal regulation* (ESTAIR; Karatzias et al., 2023, 2024) includes traditional interventions related to the processing of the trauma memories, as well as practical skills training and related interventions to improve relationships, sense of self, emotion regulation, and mood management for the DSO symptoms of CPTSD. Treatment nonresponse, however, can be as high as 50% (Kar, 2011), and individuals with childhood trauma exposure have been shown to have poorer outcomes across all intervention types (Karatzias et al., 2019), highlighting the importance of the development of interventions that address underlying developmental mechanisms influenced by early adversity.

Across the studies in this review, several limitations can be observed. All studies were cross‐sectional in nature except one longitudinal study (Menke et al., 2018). Cross‐sectional research cannot determine cause and effect and is often limited by bias (Wang & Cheng, 2020). Future research should, therefore, attempt more robust study designs. In addition, the populations used in many of the studies introduced sample bias. Many studies included student samples (*k* = 19) or samples recruited using methods that can introduce selection bias (e.g., newspaper advertisements, Amazon's Mechanical Turk [MTurk]). Although widely used, MTurk can cause validity issues due to location masking and bots, alongside improbable responses and economic conditions resulting in misrepresentation (Burnette et al., 2022). Despite this, sample characteristics were generally well reported, with strong measurement quality. Future studies should prioritize representative sampling and report sample size calculations.

The reporting of mediation results was varied. This is a common finding across mediation studies, as there is currently no standardized way to report mediation results, making heterogeneity and suboptimal reporting common (Liu et al., 2016; Vo et al., 2020). Some studies in this review did not report nonsignificant pathways, standardized estimates, and/or confidence intervals. These factors impact the interpretation of the findings. Future research should report all pathways regardless of significance and all confidence intervals in a standardized manner to facilitate the correct interpretation of results. Finally, a lack of preregistration seen across all included studies leaves uncertainty regarding data handling and the possibility of distortion. Future researchers should aim to preregister their research to increase transparency.

The limitations of the included studies should be considered along with the limitations of the review and meta‐analysis. First, a change was made to the original search procedure, as outlined in the protocol, which may have impacted the discovered literature. Further, the search strategy did not include synonyms such as “positive” or “negative.” This reflects the language used in current measurement tools; however, studies that may have utilized differing language (e.g., “positive childhood experiences”) may not have been captured adequately. Next, one researcher completed the database searches, title and abstract exclusions, and most full‐text exclusions. For the remaining full‐text exclusions, a second researcher was enlisted. This was done to reduce subjectivity within the remit of available resources. Similarly, only half of the risk of bias ratings were cross‐checked by a second researcher. Finally, it is possible that a case could be made for different categorical groupings of mediators, which may impact the results of this review. In terms of obtaining the CSIEs, many calculations had to be conducted when this information was not reported in the original papers. When possible, total scale scores were used, as this was favored over the use of subscales for simplicity. When total scores were not included, all CSIE subscale data were included. Due to the number of studies and calculations conducted, there is a risk of human error, although a second researcher checked many calculations. Some CSIE values could not be obtained, and these effects had to be excluded, which may have introduced bias. In addition, the meta‐analysis mediator groups contained small numbers, with some cases of multiple effect sizes originating from the same study; this can reduce power, with high heterogeneity seen across groups (apart from maladaptive coping), which may limit generalizability. The predominant use of cross‐sectional designs, alongside publication bias, may also limit the reliability of the meta‐analysis.

This study combined ACEs into a single broad category. To consider individual ACEs would have been beyond the scope of this review, given the substantial number of mediator pathways examined; however, it is important to note that individual ACEs (and BCEs) are often uniquely associated with mediators and symptoms (Sayyah et al., 2022). Additionally, this review did not conduct any moderation analysis to explain effect size variance. Although it is helpful to explain between‐study variance, the available data contributing to each meta‐analysis were insufficient to support reliable subgroup analyses.

This review highlights the importance of psychological mechanisms in the associations between childhood experiences and later PTSS. Specifically, this review revealed that the association between ACEs and PTSD was mediated by maladaptive coping strategies and emotion regulation. The literature on the associations between ACEs and PTSD and CPTSD has demonstrated significant indirect effects from multiple individual mechanisms across multiple categories, including cognitive beliefs and appraisals, emotion regulation strategies, interpersonal relationships, spiritual and religious beliefs, attachment style, and dissociation symptoms, with the majority of evidence belonging to the first two categories listed. Crucially, there is a gap in the literature considering pathways between BCEs and CPTSD, and not enough evidence to make any conclusions regarding the functionality of mediators in differing pathways. Preliminary evidence, however, suggests a potential protective effect of BCEs in the presence of adversity.

## AUTHOR CONTRIBUTIONS

**Sarah Louise Guthrie**: conceptualization, methodology, data curation, investigation, formal analysis, visualization, writing—original draft, writing—review and editing, validation, software, project administration. **Mark Shevlin**: conceptualization, formal analysis, methodology, supervision, writing—original draft, writing—review and editing. **Anna Bak‐Klimek**: supervision, writing—original draft, writing—review and editing. **Thanos Karatzias**: conceptualization, methodology, supervision, writing—original draft, writing—review and editing, project administration. **Amanda Woodrow**: methodology, data curation, formal analysis, supervision, validation, writing—original draft.

## AUTHOR NOTE

This research did not receive any specific grant from funding agencies in the public, commercial, or not‐for‐profit sectors. The authors declare that they have no known competing financial interests or personal relationships that could have appeared to influence the work reported in this paper.

The authors would like to thank Dr. Zoe Wagland for her contributions to the full text review and risk of bias assessment as a second reviewer.

## OPEN PRACTICES STATEMENT

This review was conducted following the Transparency and Openness Promotion Guidelines (TOP; Nosek et al., 2015). Methodology for this review was preregistered (PROSPERO registration number: CRD42023439863). All supplementary materials, data, and R code used in this study are publicly available at the Open Science Framework (https://osf.io/a7bj4/?view_only=b3616f0a6854474f927f65a0d039f607).

## Supporting information



Supplsementary materials
