# Supplementary material for: Psychological mediators of the associations between adverse and benevolent childhood experiences and posttraumatic stress disorder (PTSD) and complex PTSD: A systematic review and meta‐analysis
Source: J Trauma Stress. 2026 Jun 25;39(5):785–800. doi: 10.1002/jts.70098 (PMC13639229; doi:10.1002/jts.70098)
Supplement: Supplementary file 1 [file JTS-39-785-s001.docx]

**Contents**

The materials included are in chronological order as referenced in the main manuscript.

**Supplementary materials 1 (page 2 – 6)**

Methods

Table SA1

**Supplementary materials 2 (page – 7 – 69)**

Results

Tables SB2 – SB20

Figures S1-S6

**Supplementary materials 3 (page 70 – 83)**

Excluded studies

Funnel plots

CSIE calculations

Table SC21

**References (page 84 – 95)**

**Supplementary materials 1**

**Methods**

**Protocol pre-registration details**

The protocol was originally pre-registered on the 29th of June 2023. Post-registration, it was necessary to make some changes to the protocol. First, the initial search procedure was originally detailed to take place in two stages to capture all psychological mediator literature whereby the term ‘mediator’ may not be explicitly used. Thus, the first search would employ mediat* or associat* or relat* to capture relevant literature, and the second search would be conducted using the name of each psychological mediator (for example “ruminat*”) in place of the mediator terminology and will be rerun as per stage one for every psychological mediator identified individually from the initial search. The number of mediators identified however had a very large spread, thus it was deemed unfeasible to continue to the outlined stage two search. Despite this, a few dummy searches were conducted following the stage two search strategy, which yielded no new literature. Second, a correction was made to the quality assessment and risk of bias tools to correct an oversight where there were two quality assessment tools and no risk of bias tool. This has been updated to reflect the correct measures as used in this review, which include an adapted version of the Agency for Healthcare Research and Quality (AHRQ) assessment tool (Viswanathan, Ansari, et al., 2008) to measure risk of bias alongside the grading of recommendations, assessment, development, and evaluations (GRADE) system to assess overall quality of evidence (Guyatt et al., 2008). Both tools are non-ambiguous and have clear guidance for use, hereby minimizing any user error or subjectivity. This change was updated to the PROSPERO registry on the 14^th^ of September 2023. Finally, methodology details were amended, whereby an original plan to use methodology by Cortés-García et al (2020) was updated to reflect the use of CSIE following methodology by Woodrow (2021). This change was updated to the PROSPERO registry on the 26^th^ of February 2024.

**Definitions**

This review uses the terminology “psychological mediator” and “psychological mechanism” interchangeably. They are defined broadly, extending to all relevant factors within the psychological domain considering any aspect of cognition, behaviour, affective symptom or mood (Harvey et al., 2004). Mediators are conceptualised as a third variable (M) which aids in the understanding of the relationship between independent/predictor (X) and dependent/outcome variables (Y) (Baron & Kenny, 1986).

“Childhood experiences” are defined broadly and conceptualised into positive and negative experiences occurring before the age of 18. This includes the 10-item ACE (Felitti et al., 1998) and 10-item BCE measure definitions (Narayan et al., 2018) but also extends to any other measures of experience in childhood which can be defined as positive or negative (or combination of experiences) found in the literature (for example the Life Events Checklist [LEC] if events experienced in childhood (Weathers et al., 2013), the Child and Adolescent Trauma Screen [CATS] (Sachser et al., 2017), the Child and Adolescent Trauma Screen 2 [CATS-2] (Sachser et al., 2022), and the Child and the Childhood Trauma Questionnaire (CTQ; (Bernstein et al., 1998)).

**Data extraction**

The title and abstract review was completed by SG. Following this, a full text screening was completed independently by SG and ZW, with ZW reviewing a random 25% sample of the studies selected for full text screening. Any disagreements were resolved via discussion between SG and ZW. There was an 81% initial agreement rate between reviewers, rising to 100% after discussion (see Figure 2 main text for PRISMA flow diagram). For reasoning behind papers excluded after full text review see Supplementary materials C Table SC21.

Data was extracted from the included studies. This included study and participant characteristics (including demographics), all included variables, the measurement tool used for each variable, and all relevant statistical information. Specifically, the type of effect size reported, whether they were standardised or unstandardised, each pathway coefficient for a, b, c (direct effect between a & b), c’ (total effect between a & b including mediation effects), and ab (indirect effect), confidence intervals, alongside whether the authors reported partial or full mediation. Finally, the main study findings and limitations, clinical implications and suggestions for future research were extracted. In cases where it was not possible to directly obtain the completely standardised indirect effect (CSIE), and where requested information was not provided by authors in order to establish CSIE, calculations as per (Woodrow, 2021) (see thesis Figures 2.3 and 2.4) were utilised to obtain the CSIE and associated confidence intervals to be used in further analysis and facilitate comparison (see Supplementary materials C for full description of individual study calculations).

**Risk of bias**

To assess for risk of bias, an adapted version of the Agency for Healthcare Research and Quality (AHRQ) assessment tool (Viswanathan M et al., 2017; Woodrow, 2021) was used by two reviewers to assess across six criteria: unbiased sample selection, adequate sample description, transparency of power calculation, validated tool used for PTSD and CPTSD diagnoses, transparency of handling missing data, and use of appropriate mediator analytic methods. Studies are rated on each criterion as either “yes” (scoring 0 points), “partial” (scoring 0 points), “no” (scoring 1 point), or “unclear” (scoring 1 point). Total scores are calculated to give an overall score out of six with scores of 0-1 indicating low risk of bias, 2-4 indicating moderate risk of bias, and 5-6 indicating high risk of bias (see Table SA1 for full scoring criteria).

Measures used for ACEs, BCEs and mediators were not assessed for risk of bias as new measures are still emerging and many are therefore not yet validated. Due to the broad definition of these variables used for the inclusion criteria in this study, it was deemed to be unreasonable to downgrade studies for the use of new measurement tools.

The risk of bias assessment was conducted by SG, with a random 50% sample of papers verified by second reviewer ZW. There was an initial low 36% agreement rate between reviewers. This was primarily due to two criteria: sample size calculation (whereby reviewers initially rated differently on papers with no sample size calculation and subjectively larger sample sizes – this was rectified with clarification on whether the paper considered and justified their sample size through requirements of mediation analysis) and missing data (whereby reviewers initially rated differently on papers with unclear descriptions of how missing data was handled, this was rectified by considering if a paper had clearly described why no missing data was present and therefore why no further action was necessary). After further discussion and clarification of scoring criteria, agreement raised quickly to 100%.

Table S1: AHRQ scoring criteria (Viswanathan, Patnode, et al., 2008) adapted from Woodrow (2021).

| **AHRQ aspect** | **Criteria** | **Scoring** |
| --- | --- | --- |
| **Unbiased selection of cohort** | i) Inclusion/exclusion criteria and rationale clear ii) Recruitment strategy and rationale described clearly iii) Recruitment strategy free from bias (i.e., not advertising in specific publications, not only undergraduate students) | Yes- all criteria met  Partial- two criteria met  No- ≤ one criterion met, or recruitment strategy is deemed to be at risk of bias  Unclear- it is not clear whether a criterion is met |
| **Sample size calculation** | i) A sample size calculation was conducted and published ii) Actual recruitment meets target set using calculation (10% tolerance) iii) If no calculation is published, there was consideration of sample size requirements for mediation analysis and the sample size was consistent with this | Yes- all criteria met  Partial- sample size calculation conducted but recruitment target not met  No- no sample size calculation published and/or small sample size  Unclear- it is not clear whether any criterion was met |
| **Adequate description of cohort** | i) Reported age characteristics of sample ii) Reported sex or gender characteristics of sample iii) Reported education characteristics of sample iv) Reported ethnicity characteristics of sample v) Reported employment characteristics of sample. | Yes- four or five criteria reported (if only four reported, omitted criterion must be ethnicity, education, or employment)  Partial- two or three criteria reported, or four criteria reported with age or gender criteria omitted  No- ≤ one criteria reported, or < four criteria reported with age or gender omitted |
| **Validated PTSD/CPTSD assessment tool** | i) Valid and reliable measure used to measure presence of PTSD/CPTSD symptoms | Yes- validated measure used  Partial- measure used is validated but validation research is of poor quality or inconclusive  No- measurement tool is unvalidated  Unclear- tool used to measure DSO symptom is not described |
| **Missing data low or well-handled** | i) It is clear how missing or incomplete data was identified and accounted for ii) Missing data does not exceed 20% iii) Appropriate analytic methods were employed to minimise bias from missing data iv) There is no reason to assume that any portion of data is missing | Yes- reported no missing or incomplete data, or all four above criteria met  Partial- missing data exceeds 20%, or two to three criteria are met  No- ≤ one criteria met  Unclear- missing or incomplete data not referenced |
| **Analytic methods appropriate** | i) Analysis is appropriate (i.e., method of mediation analysis) ii) Controlled for covariates iii) Used bootstrapping or Sobel tests | Yes- all criteria met  Partial- appropriate analysis employed but no controlled covariates and/or bootstrapping/Sobel tests (downgrading will be undertaken on a case-by-case basis as some mediator models imply they are controlling for other variables without explicitly referencing this)  No- analysis not appropriate or no criterion met  Unclear- not enough information to determine suitability of analytic methods |

**Quality Assessment**

The grading of recommendations, assessment, development, and evaluations (GRADE) system was employed to assess overall quality of evidence (Guyatt et al., 2008). This assessment was conducted by SG and verified by second reviewer AW. GRADE considers 5 domains: risk of bias, imprecision, inconsistency, indirectness, and publication bias. GRADE ranks cross-sectional evidence as low quality, and upgrades or downgrades based on each of the 5 outcome domains. The score describes the overall level of certainty that the estimated effect for the outcome is close to the ‘true’ effect.

**Supplementary materials 2**

**Results**

Table S2: Characteristics and baseline demographics for included studies.

| Study | Sample (n) | Country | Age | Gender | Education | Ethnicity/  race | Employment/  income |
| --- | --- | --- | --- | --- | --- | --- | --- |
| Schumm et al (2004) | Clinical (105)  Recruited from drug and alcohol treatment centre | America - Midwestern | M = 37.4  SD = 7.86 | Female | Highest grade completed (%)  Eighth grade or less: 6  Some high school: 28  High school graduate: 37  Some college: 24  College graduate: 6 | Caucasian: N = 58  African American: N = 39  Other: N = 3 | Yearly household income reported (%):  Less than $10,000: 54  $10 - 15,000: 17  $15,000 - 25,000: 12  More than $25,000: 16 |
| Bradley et al (2005) | Clinical (134)  IPV victims presenting for care at a large urban hospital | America (not explicitly stated) | Range = 18-55  M = 34.6 SD = 9.37 | Female | 32% completed a high-school level of education or equivalent (GED)  27% reported some education beyond high school | African American | 20% currently employed  48% reported a monthly household income of less than $500.  21% reported a monthly household income between $500 and $1000 |
| Street et al (2005) | Clinical (63)  Seeking services from battered woman shelters | Southeastern United States | Range = 19-64  M = 32 | Female | 32% - some high-school education  31% - completed high school  23% - some college education  6% - completed college  8% - obtained a technical or vocational degree | White/Caucasian – 63%  African American - 29%  Latina - 5%  Other - 3% | Yearly household income reported:  Range = $0 - $35,000  M = $7,277. |
| Vranceanua et al (2007) | Clinical (100)  Recruited from a gynaecological treatment centre for low-income women | Midwestern US city | M = 28.92  SD = 10.52 | Female | Some high school completed: 24%  High school graduate: 40%  Some College: 27%  College graduate: 7%  Other: 2% | European American (48%)  African American (47%) | Employed: 27%  Yearly income < $ 10,000: 48%  $10,001 – $15,000: 19%  $15,001 – $25,000: 15%  > $25,001: 18% |
| Stevens et al (2013) | Community (139)  Recruited from a larger study of stress and immune function via posters, brochures, and staff invitations from an OB/GYN clinic | United States, Chicago | M = 28.46  SD = 7.76 | Female | 62.6% reported having completed at least some postsecondary education | African American - 83.5 %  Hispanic/Latina – 4.3%  White non-Hispanic - 5.8%  Other - 6.4% | Not reported –  Socio-economically disadvantaged female patients |
| Weiss et al (2013) | Clinical (93)  Substance use disorder patients in residential treatment | United States - Central Mississippi | Range = 19 – 61  M = 40.62  SD = 9.68 | Predominantly male (n = 71, 76.3%) | Endorsed receiving a high school education or less (n = 58, 62.4%) | Black/African American -60.2%  White - 37.6%  Hispanic/Latino - 2.2% | Most participants reported an annual income under $20,000 (n = 62, 69.7%) |
| Lilly et al (2014) | Community (99)  Ethnically diverse, community sample of women who have experienced childhood maltreatment, as well as IPV victimization in adulthood | Midwestern US | Range = 18 - 56  M = 29.72  SD = 10.11 | Female | College degree or greater – 16.1%  Some college or vocational training - 50.5%  High school degree or GED - 23.2%,  Less than a high school degree - 10.1% | African American - 55.6% European American - 29.3%  Biracial - 9.1%  Hispanic American - 3.0%  Other - 2.0%  One participant chose not to respond | Median income for the  month preceding the interview was $500 with a mean of $1,419  Most of the sample was unemployed (72.7%) |
| Murphy et al (2014) | General population (2980)  Total birth cohort of Denmark in 1984 - data from a Danish national study conducted by The Danish  National Centre for Social Research in 2008 and 2009 | Denmark | Aged 24 | Male (N = 1579)  Female (N = 1401) | Not reported | Not reported | Not reported |
| Karmel et al (2015) | Clinical (84)  Pregnant women seeking antenatal care - HIV-positive women intentionally oversampled | South Africa, Cape Town | < 18: N = 3 (3%)  18–25: N = 46 (52%)  26–35: N = 35 (40%)  36+: N = 4 (5%) | Female | Primary school: N = 13 (15%)  High school: N = 68 (77%)  Tertiary education: N = 7 (8%) | Not reported | Unemployed: N = 63 (72%)  Monthly income below 5000 rand: N = 73 (83%) |
| Van Dijke et al (2015) | Clinical (450)  Adult psychiatric inpatients - adult consecutive admissions to two centres for clinical psychotherapy | Netherlands (not explicitly stated) | M = 34.7 SD = 10.1 | 327 female | Primary and low-level secondary education = 24.4%  Middle-level secondary education = 41.1%  High-level secondary education = 34.5% | Not reported | Not reported |
| Zhang et al (2015) | Clinical (192)  recruited from a public hospital - history in the  prior year of a suicide attempt and intimate partner violence (IPV) | Southern America | M = 34.9 SD = 9.29 | Female | Not reported | African American | Unemployed: 86.7%  Individual monthly income –  $0-$249 = 56.1%  $250-$499 = 15.3%  $500-$999 = 21.7% >$999 = 6.9% |
| Broughner et al (2016) | General population (952)  Trauma exposed recruited via online survey advertised through Amazon’s Mechanical Turk (MTurk) web service | North America | M = 35.40  SD = 11.42 | 55% female | 60% completing some form of education after high school | 73 % Caucasian | 62% employed |
| Simonelli & Sacchi (2016) | Students (327)  Community sample of Italian female students | Italy | M = 23.09  SD = 2.9 | Female | College: N = 58 (17.8%)  Professional high school: N = 7 (2.1%)  Bachelor’s degree: N = 254 (77.9%)  Master’s degree: N = 4 (1.2%)  Postgraduate degree: N = 2 (0.6%)  PhD: N = 1 (0.3%) | Italian N = 311 (96.3%) | Not reported (student population) |
| Kim et al (2016) | Community (169)  Active service members in Marine Corps | Republic of Korea | Not reported | Male | High school graduate: N = 22 (13.0%)  General equivalency diploma: N = 7 (4.2%)  In College/University: N = 130 (76.9%)  In graduate school: N = 7 (4.1%)  Missing: N = 3 (1.8%) | Not reported (Korean sample) | Korean conscripts into the Marine Corps |
| McCormick et al (2017) | Students (458)  Undergraduate students recruited from a participant pool administered  by the psychology department of a regional southeastern university | America Southeastern | M = 20.70  SD = 4.205 | Mostly female (61.3%) | Not reported (student population) | Majority of participants identified as Caucasian (60.6%) and Protestant Christian (40.6%) | Not reported (student population) |
| Boykin et al (2018) | Trauma exposed students (288)  Trauma-exposed sample of college women recruited from introductory and upper-level psychology courses at a large Midwestern university | America Midwestern | M = 19.22  SD = 1.46 | Female | First (56.9%) or second undergraduate  year (21.5%)  Completed three or more undergraduate years (16.0% juniors, 4.5% seniors, 1.0% “other”) | White = 60.1% Black = 21.9%  Other/multiracial = 9.7%  Asian or South-Asian = 3.1%  Native Hawaiian or Pacific Islander = 1.0% Preferred not to respond = 4.2%  Non-Hispanic = 80.9%  Hispanic = 18.8% Preferred not to respond = 0.3% | Not reported (student population) |
| Kratzer et al (2018) | Clinical (218)  Severely traumatized inpatients with PTSD | Germany | M = 47.7 SD = 10.3 | Female  N = 182  Male  N = 36 | 207 patients (95%) had some kind of secondary school qualifications  42 (19%) had graduated from university | 199 patients (91%) were German citizens | 43 (20%) worked full-time  170 (78%) received either sickness benefits, unemployment benefits,  (early) pension and/or social care |
| Menke et al (2018) | Community (100)  Postpartum women - subset of a larger, longitudinal parent  study examining the effects of maternal history of CM on mothers’ and infants’ adjustment  and parenting outcomes | America (not explicitly stated) | M = 29.5 SD = 5.9 | Female | Less than high school: 5%  High School degree or General Educational Development: 6%  Some college: 25%  AA degree: 8%  Vocational or technical degree: 3%  Bachelor’s degree: 29%  Master’s degree: 17%  Doctoral degree: 6%  High school or less: 11% | Caucasian: 65%  African American: 21%  Asian or Pacific Islander: 6%  Latino: 3%  Biracial and other: 4%  Minority status: 34% | Income:  Less than US $15,000: 16%  US $I5,000–US $25,000: 11%  US $25,000–US $50,000: 23%  US $50,000+: 49%  Less than US $20,000: 23% |
| Van Dijke et al (2018) | Clinical (472)  Adult psychiatric patients from a tertiary mental health institution diagnosed with severe and chronic psychiatric and personality disorders | Netherlands (not explicitly stated) | M = 34.7 SD = 10.1 | Male = 32.25% | Educational level:  Low (CPTSD):  M = 36 SD = 29  Low (no CPTSD):  M = 74 SD = 23  Middle (CPTSD):  M = 42 SD = 33  Middle (no CPTSD): M = 140 SD = 43  High (CPTSD):  M = 48 SD = 38  High (no CPTSD):  M = 109 SD = 34 | Not reported | Not reported |
| Vang et al (2018) | Clinical (99)  Highly symptomatic, treatment-seeking females. Participants referred by general practitioners, psychiatrists, or psychologists for psychological therapy to an NHS trauma centre | Scotland | Range = 19 – 62  M = 38.96  SD = 10.78 | Female | The majority had finished post-secondary education (69.7%) | The  sample was primarily of British origin (90.6%) | Currently unemployed (58.2%) |
| Wilson & Newins (2018) | Students (336)  The participants were recruited through a university psychology subject pool at a small mid-Atlantic public university using an online research management system and through social media sites | Mid-Atlantic | Range: 18 – 77  M = 22.81  SD = 8.84 | Female: 236 (70.2%)  Men: 96 (28.6%)  Transgender or  other gender: 4 (1.2%) | Not reported (student population) | Caucasian/White:  N = 255 (75.9%) Hispanic/Latino  N = 21 (6.3%)  African American/Black/African origin:  N = 19 (5.7%)  Asian American/Asian Origin/Pacific Islander:  N = 17 (5.1%)  American Indian/Alaska Native:  N = 3 (0.9%) Middle Eastern:  N = 2 (0.6%) Bi-racial/multi-racial:  N = 18 (5.4%) “Other”  N = 1 (0.3%) | Not reported (student population) |
| Ironson et al (2019) | Clinical (290)  Data collected from a two-site (Boston, MA and Miami, FL) randomized clinical trial (RCT)  of HIV-uninfected MSM reporting condomless sex and a history of CSA before the age 17 (N = 290). Data from a  sub study of a larger RCT comparing two treatments: safe sex reduction plus CBT for trauma versus safe sex reduction plus supportive  therapy used. | Boston, MA and Miami, FL | M = 37.95  SD = 11.68 | Male | < Some high school or GED:  N = 73 (24.7%)  Some college:  N = 106 (35.8%)  College graduate:  N = 71 (24.0%)  Graduate degree:  N = 39 (13.2%) | Euro American:  N = 201 (69.3%)  African American:  N = 66 (22.8%)  Asian or Pacific Islander:  N = 11 (3.8%)  Other:  N = 12 (4.1%)  Ethnicity: Latino: N = 87 (30.0%) | Income  < 10,000 per year:  N = 88 (29.4%)  10,001–20,000:  N = 66 (22.3%)  20,001–40,000:  N = 53 (17.9%)  > 40,000 per year:  N = 83 (27.8%) |
| Cloitre et al (2019) | Clinical trauma exposed (290)  Baseline data of 290 women with DSM–IV PTSD related to interpersonal violence enrolled in a randomized controlled trial | America (not explicitly stated) | M = 42.5 SD = 11.9 | Female | High school or less: 24.1%  Some college or grad: 56.9%  More than college: 19.0% | White: 45.3%  Black: 33.2%  Other: 21.5%  Non-Hispanic: 80.3%  Hispanic: 19.7% | Employed full- or part-time: 28.3% Homemaker/student/ volunteer/retired: 9.3%  Unemployed/disabled: 62.4% |
| Haj-Yahia et al (2019) | Students (516)  Israeli  university and college students | Jerusalem | M = 24.9, SD = 2.7 | Female: 90.7% Male: 9.3% | Not reported (student population) | 91.5% were Jews  The remaining 8.5% were Arabs | Participants' perceptions of their families' socio-economic status:  4.7% defined their families as high-class, 36.6% as upper middle-class, 44% as middle-class, 11.3% as lower middle-class, and 3.5% as socio-economically deprived |
| Hosseini Ramaghan et al (2019) | Clinical (432)  Adult patients referred for treatment to psychological,  psychiatric and medical clinics | Shiraz, Iran | Range = 18 - 58  M = 32.50  SD = 9.32 | Female: 278 Male: 154 | Not reported | Not reported | Not reported |
| Knefel et al (2019) | Clinical (193)  Participants referred by general practitioners, psychiatrists, or psychologists for psychological therapy to an NHS trauma centre | Scotland | M = 40.7 SD = 12.4 | Female: 65.1% | The highest level of academic attainment was varied: school (38.5%), College (30.2%) and University (30.2%) | Most born in the United Kingdom (88.7%) and from Scotland (79%) | Approximately a third of the sample was in employment (full-time 20.2%, part-time 13%), 38.9% were unemployed, 7.3% were retired and 5.7% were in voluntary work (15% reported ‘None of these’) |
| McLafferty et al (2019) | Community (veterans) (3092)  US military veterans. The NESARC-III, a cross-sectional study, was designed to be representative of the adult, non-institutionalised population of the US | United States | Range = 19 - 90  M = 60  SD = 16.03 | Male: 2788 Female: 304 | Not reported | White, non-Hispanic: 79.5%  Black, non-Hispanic: 10.5%  Hispanic: 6.5%  American Indian/Alaska native, non-Hispanic: 1.9% Asian/Native Hawaiian/other Pacific Islander, non-Hispanic: 1.6% | 57.2% of veterans earned less than $60,000 per annum, with 13% earning more than $120,001 |
| Demir et al (2020) | Community (refugees) (89)  In Berlin (N = 49), participants were recruited at  the Central Clearing Clinic, an outpatient institution specialized in offering psychiatric services for refugees and collaborating with multiple refugee camps and civic initiatives. In  Amman (N = 40), participants were recruited by the German  humanitarian NGO “Help-Hilfe zur Selbsthilfe”. | Germany and Jordan | M = 34.0 SD = 10.18 | Female: 53.4% | Master’s degree: 19.3%  Bachelor’s degree: 13.6%  High school certification: 27.3%  Lower or none school graduation: 39.8% | Not reported (Syrian refugee population) | Not reported (Syrian refugee population) |
| Huang et al (2020) | Clinical & Community (EG – 221, CG – 119)  Adults who had experienced childhood neglect and abuse (n = 295, 184 patients with personality disorder and 111 community controls) recruited from a large-scale study in Greater London investigating personality disorders with a computational psychiatry approach | London | EG:  Range = 18–65  M = 31.51  SD = 10.32  CG:  Range = 18–52  M = 28.31  SD = 9.33 | EG: 58% female  CG: 58.4% female | Not reported | Not reported | Not reported |
| McCuaig Edge & Lee (2020) | Community (Armed Forces) (2927)  Canadian Armed Forces (CAF) personnel who had completed a postemployment assessment upon return from an overseas deployment | Canada | Range = 19 - 55  M = 27.0  SD = 5.3 | Male: 90.4% | Not reported | Not reported | Not reported (Canadian Armed Forces sample) |
| Mitchell et al (2020) | Clinical (106)  Sample of trauma-exposed, treatment  receiving adults recruited from four sites across  two health trusts | Northern Ireland | M = 47.34  SD =  10.82 | Male:  N = 71  Female: N = 34 Unreported:  N = 1 | Not reported | Not reported | Not reported |
| Vasilopoulou et al (2020) | Clinical (42)  Older adults who were seen for psychological  therapy by psychologists, psychiatrists or community mental health clinicians within the NHS Older Adult services | Scotland | M = 71.5 SD = 4.6 | Male:  N = 11 (26.2%)  Female: N = 31 (73.8%) | M = 13  SD = 3.6  Scale not specified | White British: N = 22 (52.4%)  White Scottish:  N = 19 (45.2%)  White Other: N = 1 (2.4%) | Employed: N = 4 (9.5%)  Retired: N = 38 (90.5%) |
| Batchelder et al (2021) | Clinical (290)  Data from 290 HIV-uninfected MSM with histories of CSA were collected as part of a  comprehensive psychiatric baseline assessment from a multi-site randomized clinical trial | Boston and Miami | Range = 18 – 67  M = 37.95  SD = 11.68 | Male | One quarter of the sample reported  having up to a high school education, 36% some college, 24% some graduate school, and  13% reported completing a graduate degree | White: 68%  Black or African American: 22%  Other: 10%  Latino: 29% | Income:  ≤ $10,000:  N = 88 (29.7%)  $10,001-$20,000:  N = 66 (22.3%)  $20,001-$40,000:  N = 53 (17.9%)  ≥$40,001:  N = 83 (28.0%) |
| Eising et al (2021) | Community (116 EG,  122 CG)  Half were affected by compulsory social measures and placements (CSMP) in childhood and/or adolescence (EG). The other half were an age-matched control group (CG) | Switzerland | M = 70.25 (EG)  M = 70.71 (CG) | 41% female (EG)  51% female (CG) | EG:  No education:  N = 5 (4.3%)  Primary education: N = 8 (6.9%)  Upper secondary education:  N = 19 (16.4%)  High school:  N = 2 (1.7%)  Vocational job training:  N = 50 (43.1%)  Higher professional training:  N = 18 (15.5%)  University:  N = 7 (6%)  Other: N = 7 (6%)  CG:  No education:  N = 0 (05)  Primary education:  N = 1 (0.8%)  Upper secondary education:  N = 5 (4.1%)  High school:  N = 4 (3.3%)  Vocational job training:  N = 43 (35.2%)  Higher professional training:  N = 19 (15.6%)  University:  N = 45 (36.9%)  Other:  N = 5 (4.1%) | Older adults from the Swiss-German part of Switzerland | Not reported |
| Chen et al (2021) | Clinical (108)  Recruited after visiting hospital emergency departments (EDs) in Ohio (USA) within 48 hours after a traumatic event. Participants were recruited as part of a larger longitudinal study investigating brain changes predicting development of PTSD over a one-year period after trauma exposure | Ohio | M = 33.1 SD = 10.6 | Mostly  female (65.7 %) | Not reported | Black or African American:  N = 57 (52.8%)  White:  N = 41 (38.0%)  Asian:  N = 1 (0.9%)  Other:  N = 2 (1.9%)  Unknown:  N = 7 (6.5%) | Not reported |
| Chi et al (2021) | Students (2038)  Chinese university students who have or have not been exposed to domestic violence from more than 180 universities | China | M = 20.56  SD = 1.90 | Male:  N = 755 Female: N = 1286 | Not reported (student population) | Not reported | Subjective social economic status: N = 4.85  SD = 1.38 |
| Espeleta et al (2021) | Students (376)  College students from a large  Midwestern University | Midwestern America | M = 19.83  SD = 1.81 | Female: N = 244 (64.9%)  Male:  N = 131 (34.8%) Chose not to respond: N = 1 (0.3%) | Not reported (student population) | Caucasian: 79.8%  African American: 7.7%  Latino/a:  3.7%  Asian: 1.6%  Chicano/a:  0.5%  Middle Eastern:  0.5%  Other/chose not to respond: 6.1% | Not reported (student population) |
| Goncharenko et al (2021) | Community  (Trauma-exposed military veterans) (465)  Recruited from Amazon’s Mechanical Turk  (MTurk) | North America | M = 38.01 | Female: 71.6% | Not reported | Hispanic/Latin: N = 112 (24.6%)  Not Hispanic/Latin: N = 344 (75.4%)  White:  N = 323 (69.5%) African American/Black:  N = 108 (23.2%)  Asian:  N = 26 (5.6%) American Indian/Alaskan Native:  N = 19 (4.1%) Native Hawaiian/Other Pacific Islander:  N = 5 (1.1%) | Full-time: N = 394 (85.7%)  Part-time: N = 40 (8.7%)  Not in Labor Force (Student, Homemaker):  N = 14 (3.0%)  Unemployed:  N = 12 (2.6%) |
| Guo et al (2021) | Students (1361)  Students  who came from more than 130 universities in China | China | M = 20.73  SD = 1.88 | Male:  N = 465 (34.2%) Female: N = 896 (65.8%) | Not reported (student population) | Not reported | Subjective social-economic status:  M = 4.89  SD = 1.38 |
| Kim et al (2021) | Community (212)  Crime victims with PTSD recruited through the referral from the police stations in Seoul  metropolitan areas of South Korea | South Korea | M = 39.9 SD = 13.3 | Female: N = 174 (82.1%) | Education (years)  M = 13.0  SD = 3.02 | Not reported | Not reported |
| Lagdon et al (2021) | Students (640)  University  students from Northern Ireland | Northern Ireland | Maltreated Group:  N = 167 M = 22.00  Nonmaltreated Group:  N = 473 M = 21.00 | Maltreated group:  Male:  N = 37 (22.16%) Female: N = 130 (77.84%)  Non-maltreated group: Male:  N = 119 (25.16%) Female:  N = 354 (74.84%) | Not reported (student population) | Not reported | Not reported (student population) |
| Seitz et al (2021) | Community (85)  Participants were part of a larger study on the shared effects of CTEs on social information processing across different psychiatric disorders; the larger study included a healthy volunteer group  Participants with psychiatric disorders were recruited through clinical referrals from inpatient and outpatient units as well as advertisements | Germany | Range: 18–59  M = 31.3  SD = 11.1 | Female: N = 67 | Educational level years:  M = 31.31  SD = 11.12 | Not reported | Not reported |
| Sistad et al (2021) | Students (586)  Archival data - recruited from a  Midwestern university through online, flyers, and class announcements | Midwestern America | Range:  18 - 25  M = 19.58  SD = 1.57 | Female: 70.7 % | Not reported (student population) | White: 87%  African American: 3%  Native American: 3%  Asian: 2%  Multiracial: 2%,  Native Hawaiian or Other Pacific Islander: 1%  Chose not to respond: 2% Hispanic: 2% | Not reported (student population) |
| Watts et al (2021) | Students (396)  Convenience sample of  college students at two universities in the southern region of  the United States | Southern region of  the United States | M = 23.63  SD = 6.91 | Female: 79.8%  Male: 19.4%  Transgender:  0.3%  Ambonec/nonbinary: 0.5% | Not reported (student population) | White/Caucasian: 42.7%  Hispanic/Latino(a): 21.2% African American/Black: 23.0%  Asian American/Pacific Islander: 9.6%  Other: 3.5% | Over half (66.2%) of participants were employed |
| Alvarez et al (2022) | Clinical (336)  Women presenting to an urban, primary care setting were approached and  invited to participate in a study about stress and women’s health | America | M = 32 SD = 7.9 | Female | Most participants had less than a high school education (58%; 32% with elementary or less) | Most participants were from the Northern Triangle: Honduras (34%), El Salvador (24%), and Guatemala (11%). Other represented countries included Mexico (18%), Ecuador (7%), and others (Bolivia, Nicaragua, Panama, Dominican Republic, and Venezuela; 6%) | Unemployed: 62% |
| Fitzgerald et al (2022) | Students (177)  Participants were either undergraduate or graduate students enrolled at a southern university | Southern America | Range  18 -58  M = 27.76  SD = 9.74 | Female: N = 163 (92.1%), Male:  N = 13 (7.3%)  Nonbinary:  N = 1 and (.6%) | Not reported (student population) | Among the participants, 90 reported being racial or ethnic minorities (50.8%) and 87 reported being White (49.2%) | Participants tended to report income below $20,000 per year (78.3%) |
| Jernslett et al (2022) | General population (898)  The present study is part of a larger longitudinal pan-European project ‘ADJUST Study’, which explores associations between  risk and resilience factors, stressors, and symptoms of mental health during the COVID-19 pandemic. The  present study used first-wave data from Greece | Greece | Range: 18–83  M = 35.9 SD = 12.5 | Female: N = 753 68% Male:  N = 354 32% | Less than 6 years of schooling:  N = 11 (1%)  6–9 years of schooling:  N = 22 (2%)  10–13 years of schooling:  N = 171 (15.4%) Completed vocational studies: N = 96 (8.7%) Completed university studies: N = 538 (48.6%) Completed postgraduate studies:  N = 269 (24.3%) | Not reported | Income:  Very low (<€500):  N = 110 (9.9%)  Low (€500 to <€1000):  N = 390 (35.2%)  Low moderate (€1000 to <€2000):  N = 371 (33.5%)  High moderate (€2000 to <€3000):  N = 137 (12.4%)  High (≥€3000):  N = 58 (5.2%)  Other:  N = 41 (3.7%) |
| Jowett et al (2022) | General population  (1020)  A nationally representative sample from Ireland - noninstitutionalized Irish adults aged 18 years and older | Ireland | Range: 18 – 87  M = 43.10  SD = 15.12 | Female: 51.0% | Secondary-school completion was the highest educational attainment for 39.2% of participant, 36.9% completed an undergraduate degree, 16.9% completed a postgraduate degree, and 7.1% did not complete secondary school (equivalent to high school in the U.S.) | Not reported:  Most participants (53.9%) resided in Leinster (east of the country including the capital city of Dublin), 26.9% resided in Munster (south of the country), 13.5% resided in Connaught (west of the country), and 5.7% resided in Ulster (north of the country) | Nearly half of participants were in full- time employment (45.8%), 17.8% were in part-time employment, 27.7% were retired, homemaking, or a student, and 8.6% were unemployed |
| Maercker et al (2022) | Community (126)  Swiss individuals who were at least 50 years of age and had been affected by CSMP in their childhood/adolescence & a group of age-matched control individuals | Switzerland | EG:  N = 27  M = 70.8 CG:  N = 125  M = 70.6 | EG:  N = 26 (41.7% female) CG:  N = 125 (51.2% female) | Educational attainment (years)  EG:  M = 11.58 SD = 4.99 Range = 0-17  CG:  M = 14.56 SD = 3.65 Range = 0-17 | Swiss individuals | Average monthly income (CHF):  < 2001 RG:  N = 31 24.3%  < 2001 CG:  N = 11 8.8%  2001-4670 EG:  N = 51 40.9%  2001-4670 CG:  N = 40 32.0%  > 4670 RG:  N = 44 34.8%  > 4670 CG:  N = 74 59.2% |
| Annett et al (2023) | Community (incarcerated women) (488)  500 incarcerated women were randomly selected from eight jails in Kentucky, screened for opioid use disorder (OUD), and invited to participate in a larger clinical trial to increase OUD treatment access and initiation | Kentucky, America | Range = 20 - 62  M = 36.8 | Female | High school diploma/GED or higher: 72.4% | White: 95.1% | Employed prior to incarceration (full time, part time, or day labour): 23.6% |
| Békés et al (2023) | Clinical (226)  Psychiatric inpatients | Hungary | M = 31.57  SD = 11.69 | Female: 71.7% | Not reported | Not reported | Not reported |
| Feiler et al (2023) | Students (1498)  Students were recruited from introductory psychology courses | United States | Range = 18 - 29  M = 19.8 SD = 2.4 | Female: N = 1045 69.8%  Male:  N = 430 28.7% | Student Status:  Freshman (1–29 h): N = 741 49.5%  Sophomore (30–59 h): N = 326 21.8%  Junior (60–89 h): N = 175 11.7%  Senior (90 or more hours):  N = 138 9.2%  Graduate or professional student:  N = 11 17.4%  Other:  N = 7 0.5%  Missing:  N = 0 0% | White or European:  N = 868 57.9% Black or African American:  N = 229 15.3%  Latina/o/x or Hispanic:  N = 179 11.9% Multiple race/ethnicity: N = 87 5.8% Asian:  N = 49 3.3%  Native American, American Indian, or Alaska Native:  N = 7 0.5% West Asian, Middle Eastern, or North African: N = 8 0.5% Native Hawaiian or Other Pacific Islander:  N = 3 0.2% Chose not to disclose:  N = 11 0.7% Missing:  N = 57 0.4% |  |
| Fitzgerald & Lunt (2023) | Students (219)  University students | Southern United States | M = 29.17  SD = 10.47 | Female | Not reported (student population) | White/European American: N = 125 (57.1%)  Black/African American:  N = 82 (37.4%)  Hispanic/Latino: N = 5 (2.3%)  Native American/American Indian:  N = 1 (.5%)  Multi/biracial: N = 6 (2.8%) | Not reported (student population) |
| Kiefer et al (2023) | Trauma exposed community (320)  Trauma-exposed  community individuals who reported past 30-day substance use recruited from Amazon’s Mechanical Turk (MTurk) | North America | M =  35.78 | Male: 50.3% | Not reported | Hispanic/Latin: N = 44 (14.3%)  Not Hispanic/Latin: N = 264 (85.7%)  White: N = 261 (81.6%)  African American/Black: N = 41 (12.8%)  American Indian/Alaskan Native:  N = 14 (4.4%)  Asian: N = 9 (2.8%)  Other/Unspecified: N = 8 (2.5%) | Full-time:  N = 258 (81.6%)  Part-time:  N = 34 (10.8%)  Not in Labor Force (Student, Homemaker):  N = 13 (4.1%)  Unemployed: N = 11 (3.5%) |
| Li & Liang (2023) | Students (360)  Chinese college students | China | M = 20.71  SD = 1.69 | Female:  N = 206  Male:  N = 54 | Not reported (student population) | Not reported | Not reported (student population |
| Russo et al (2023) | Trauma exposed students (385)  Participants who endorsed a history of childhood trauma. The data used in this study originate from a larger online survey on lifetime trauma exposure and outcomes among undergraduate students | Southern United States | Range = 18 – 48  M = 20.02  SD  = 3.47 | Female:  71.9% | Not reported (student population) | White: 69.9%  Black: 26.0%  Asian/Asian-American: 4.4%  American Indian/Alaska Native: 1.8% Native Hawaiian/Pacific Islander: 0.5%  Other ethnicities: 0.5% | Not reported (student population |
| Darnell et al (2024) | Students (203)  Recruited from undergraduate introductory psychology classes - mediation conducted with participants who scored above the cut-off on at least one scale of the Childhood Trauma Questionnaire | Midwest, United States | Range: 18 – 34 M = 19.5 SD = 1.9 | Female: 62.1% | Not reported (student population) | White: 54.5%  Black: 23.6%  Asian: 8.2%  Other: 12.6%  Non-Hispanic: 74.9%  Hispanic: 25.1% | Not reported (student population) |
| Haim-Nachum et al (2024)^1^ | Trauma exposed community (230)  Israeli participants with general lifetime trauma | Israel | M = 46.30  SD = 15.00 | Female: 52.2% | Education (years): M = 15.48  SD = 2.83 | Not reported (Israeli sample) | Not reported |
| Haim-Nachum et al (2024)^2^ | Childhood trauma exposed community (685)  US residents from MTurk | US | M = 36.8  SD = 11.3 | Female (n = 407, 63.4%) | Not reported | Race & ethnicity Hispanic/Latinx (n = 78, 12.1%)  Non-Hispanic White (n = 446, 69.5%) Non-Hispanic Black (n = 76, 11.8%)  Non-Hispanic Asian (n = 25, 3.9%)  Non-Hispanic Native American/Pacific Islander (n = 5, 0.8%) Non-Hispanic Other (n = 12, 1.9%) | Socio-economic levels:  M = 4.5  SD = 1.8) |
| Kitano et al (2024) | Trauma exposed community (752, 537 with ACEs)  Japan air self-defence force new recruits | Japan | M = 20.60  SD = 2.99 | Male = 560 (74.5)  Female = 182 (24.2%)  Other = 10 (1.3%) | High school (n = 444, 59%)  Technical college (n = 58, 7.7%)  Junior college (n = 22, 2.9%)  University (n = 174, 23.1%)  Other (n = 54, 7.2%) | Not reported | Not reported (air-defence force recruits) |
| Arslan et al (2025) | Student population (474)  Recruited from a public university in Türkiye | Türkiye | M = 20.49  SD = 1.45 | Female (70%) | Not reported (university students) | Not reported | Socioeconomic status [SES]:  Low SES = 17 %  Moderate SES = 49 %  High SES = 34 % |
| Özdemir & Yazici (2025) | Community (366)  Participants involved in a romantic relationship for at least 3 months, trauma exposed, internet recruited | Turkey | M = 30.23  SD = 8.14 | Female (n = 311, 85%)  Male (n = 55, 15%) | Primary school (n = 2, 0.5%)  Secondary school (n = 2, 0.5%)  High school (n = 33, 9%)  Bachelor’s degree (n = 259, 70.8%) Master’s degree (n = 56, 15.3%) Doctorate (PhD) (n = 14, 3.8%) | Not reported | Full-time employed (n = 178, 48.6%)  Part-time employed (n = 27, 7.4)  Not in work (n = 14, 3.8%) |
| Watts et al (2025) | Student sample (263) | US | M = 27.19  SD = 10.45  Range = 18 - 62 | Female (n = 202, 76.8%)  Male (n = 43, 16.3)  Nonbinary (n = 14, 5.3%)  Another not listed (n = 1, 0.4%)  Prefer not to say (n = 3, 1.1%) | College students | White (n = 117; 44.5%)  Hispanic/Latinx (n = 56; 21.3%)  African American/Black (n = 47; 17.9%)  Asian American/Pacific Islander (n = 29; 11.0%)  Native American (n = 3; 1.1%). Another race/ethnicity not listed (n = 11; 4.2%) | Employed full time (n = 92; 35.0%)  Part time (n = 94; 35.7%  Unemployed (n = 77; 29.3%) |

Table S3: Study variables by mediator category.

| Category | Study | Predictor  (measure) | Outcome  (measure) | Mediator  (measure) |
| --- | --- | --- | --- | --- |
| Cognitive beliefs & appraisals | **Schumm et al (2005)** | Child physical abuse and child sexual abuse  (abbreviated Childhood Trauma Questionnaire) | PTSD  (The PTSD Symptom Scale-Interview) | **Interpersonal resource loss**  (Nine items from the Conservation of Resources- Evaluation) |
|  | **Street et al (2005)** | Potentially traumatic events in childhood  (The Traumatic Stress Survey) | PTSD  (PTSD Symptom Checklist - Civilian Version) | **Trauma related guilt**  (Trauma-Related Guilt Inventory) |
|  | **Bradley et al (2005)** | Childhood maltreatment  (Childhood Trauma Questionnaire) | PTSD  (Davidson Trauma Scale) | **Self-esteem**  (Taylor Self-Esteem Inventory) |
|  | **Murphy et al (2014)** | Childhood maltreatment  (Interview - several questions across four domains of childhood maltreatment: physical abuse, emotional abuse, neglect, and sexual abuse | PTSD  (the Primary Care PTSD Screen) | **Self-esteem**  (The Rosenberg Self-Esteem Scale) |
|  | **Kim et al (2017)** | Childhood maltreatment  (Childhood Trauma Questionnaire—Short Form Korean Version) | PTSD  (Impact of Event Scale —Revised – Korean Version) | **Resilience**  (Connor – Davidson Resilience Scale Korean Version) |
|  | **Boykin et al (2018)** | Childhood maltreatment  (Childhood Trauma Questionnaire) | PTSD  (PTSD checklist for DSM-5) | **Psychological inflexibility**  (Acceptance and Action Questionnaire) |
|  |  |  |  | **Fear of self-compassion**  (Fears of compassion scale) |
|  | **Menke et al (2018)** | Childhood maltreatment  (Interview - trauma history table developed for this study) | PTSD  (The National Women’s Study PTSD Module) | **Maltreatment specific shame**  (Interview) |
|  | **Wilson & Newins (2018)** | Childhood maltreatment  (Childhood Trauma Questionnaire Short Form) | PTSD  (PTSD Checklist for DSM-5) | **Anxiety sensitivity (cognitive concerns)**  (18-item Anxiety Sensitivity Index-3) |
|  | **Ironside et al (2019)** | Childhood sexual abuse  (clinician-administered structured interview) | PTSD  (Davidson Trauma Scale) | **Post-traumatic cognitions**  (The Posttraumatic Cognitions Inventory) |
|  | **Hosseini Ramaghani et al (2019)** | Childhood trauma  (The Childhood Trauma Questionnaire) | PTSD  (The Post-traumatic Checklist for DSM-5) | **Metacognition**  (The Metacognitions Questionnaire) |
|  |  |  |  | **Time perspectives**  (Zimbardo Time Perspective Inventory) |
|  | **Huang et al (2020)** | Childhood trauma  (Childhood Trauma Questionnaire) | PTSD  (The Posttraumatic Stress Disorder Checklist Scale) | **Mentalizing**  (The Reflective Functioning Questionnaire) |
|  | **McCuaig Edge and Lee (2020)** | Childhood neglect and adversity  (ACE Questionnaire) | PTSD  (PTSD Checklist–Civilian version) | **Alexithymia**  (Toronto Alexithymia Scale) |
|  | **Mitchell et al (2020)** | Childhood trauma  (Childhood Trauma Questionnaire) | PTSD  (Posttraumatic Stress Diagnostic Scale) | **Trauma appraisals**  (Trauma Appraisal Questionnaire) |
|  | **Vasilopoulou et al (2020)** | Childhood trauma  (Childhood Trauma Questionnaire) | PTSD & CPTSD  (International Trauma Questionnaire) | **Early maladaptive schemas**  (Young Schema Questionnaire Short Form, 3rd Edition) |
|  | **Chen et al (2021)** | Childhood maltreatment  (Childhood Trauma Questionnaire) | PTSD  (The Clinician -Administered PTSD Scale) | **Dispositional optimism/pessimism**  (The Revised Life Orientation Test) |
|  | **Chi et al (2021)** | Childhood exposure to domestic violence  (Revised Adverse  Childhood Experiences Questionnaire Chinese version) | PTSD  (Abbreviated PTSD Checklist - Civilian version) | **Self-compassion**  (The Self-compassion Scale) |
|  |  |  |  | **Resilience**  (Connor–Davidson Resilience  Scale Short Version) |
|  | **Guo et al (2021)** | Adverse childhood experiences  (The Revised Adverse Childhood Experiences Scale) | PTSD & CPTSD  (The International Trauma Questionnaire) | **Self-kindness**  (Self-Compassion Scale – Short Form – subscale) |
|  |  |  |  | **Self-judgement**  (Self-Compassion Scale – Short Form – subscale) |
|  | **Kim et al (2021)** | Childhood abuse  (Life Events Checklist) | PTSD  (The Clinician-Administered PTSD Scale (CAPS) for Diagnostic and Statistical Manual of Mental Disorders,  Fifth Edition (DSM-5)) | **Low resilience**  (Connor-Davidson Resilience Scale) |
|  | **Watts et al (2021)** | Childhood emotional maltreatment  (Emotional Abuse subscale of the Childhood Trauma Questionnaire) | PTSD  (Short Post-Traumatic Stress Disorder Rating Interview) | **Event centrality**  (Centrality of Event Scale) |
|  | **Li & Liang (2023)** | Childhood trauma  (The Life Events Checklist for DSM-5) | CPTSD  (The International Trauma Questionnaire) | **Self-esteem**  (The Rosenberg Self-Esteem Questionnaire) |
|  | **Darnell et al (2024)** | Childhood stressful life events  (Childhood Trauma Questionnaire) | PTSD  (PTSD Checklist for DSM-5) | **Metacognition**  (The Metacognitions Questionnaire – PTSD) |
|  | **Haim-Nachum et al (2024)** | Childhood maltreatment  (adapted Adverse Childhood Experiences Scale) | PTSD  (Primary Care PTSD Screen for DSM-5) | **Self-stigma**  (Internalized Stigma subscales, the Secrecy subscale, & the Recovery Assessment Scale) |
|  | **Kitano et al (2024)** | Adverse Childhood Experiences  (Child Abuse and Trauma Scale) | PTSD  (Post-traumatic  Stress Disorder Checklist for Diagnostic and Statistical Manual for Mental Disorders 5) | **Self-compassion**  (Self-Compassion Scale) |
|  | **Arslan (2025)** | Adverse Childhood Experiences  (Adverse Childhood Experiences (ACEs) Checklist | PTSD  (Posttraumatic Stress Disorder Checklist) | **Self-compassion**  (The Self-Compassion Scale-Short Form) |
|  |  |  |  | **Psychological flexibility**  (The Avoidance and Fusion Questionnaire for Youth) |
|  | **Watts (2025)** | Childhood Psychological Abuse  (Childhood Trauma Questionnaire) | CPTSD  (International Trauma Questionnaire | **Posttraumatic appraisals**  (Trauma Appraisal Questionnaire) |
| Emotion regulation strategies | **Stevens et al (2013)** | Childhood abuse  (The Childhood Trauma Questionnaire) | PTSD  (The PTSD Symptom Scale–Self Reported) | **Emotion regulation difficulties**  (The Difficulties with Emotion Regulation Scale) |
|  | **Weiss et al (2013)** | Childhood abuse  (The Childhood Trauma Questionnaire-Short Form) | PTSD  (The PTSD Checklist-Civilian Version) | **Emotion dysregulation**  (The Difficulties in Emotion Regulation Scale) |
|  | **Lilly et al (2014)** | Childhood trauma exposure  (The Childhood Trauma Questionnaire) | PTSD  (The Posttraumatic Stress  Diagnostic Scale) | **Emotion regulation**  (The Difficulties in Emotion Regulation Scale) |
|  | **Choi et al. (2015)** | Childhood trauma  (Childhood Trauma Questionnaire Short Form) | PTSD  (Davidson Trauma Scale) | **Maladaptive coping**  (The Brief COPE) |
|  | **Boughner et al (2016)** | Childhood psychological trauma exposure  (Adverse Childhood Experiences Questionnaire) | PTSD  (PTSD Checklist for DSM-5) | **Mindfulness related traits**  (Five Facet Mindfulness Questionnaire–Short Form) |
|  | **Kratzer et al. (2018)** | Childhood abuse  (The German version of the Childhood Trauma Questionnaire) | PTSD  (The German version of the Impact of Events Scale-R) | **Mindfulness**  (The Freiburg Mindfulness Inventory) |
|  | **Van Dijke et al (2018)** | Childhood trauma  (Traumatic Experiences Checklist) | CPTSD  (Dutch self-report version of the Structured Interview for Disorders of Extreme Stress Not Otherwise Specified) | **Affect dysregulation**  (Over-regulation of affect - The Bermond Vorst Alexithymia Questionnaire. Under-regulation of effect - ‘Affect instability scale’ from the BPDSI) |
|  | **Cloitre et al (2019)** | Adverse childhood experiences  (18-item Life Stressor Checklist) | PTSD  (The Posttraumatic Stress  Disorder Checklist for DSM–5) | **Emotion regulation**  (The Difficulties in  Emotional Regulation Scale) |
|  | **Knefel et al (2019)** | Childhood maltreatment  (The Childhood Trauma Questionnaire) | PTSD & CPTSD  (The International Trauma Questionnaire) | **Emotion regulation**  (The Difficulties in  Emotional Regulation Scale) |
|  | **Demir et al (2020)** | Early adversity  (The Childhood Trauma Questionnaire) | PTSD  (The Harvard Trauma Questionnaire) | **Cognitive emotion regulation**  (The Cognitive Emotion Regulation Questionnaire) |
|  | **Batchelder et al (2021)** | Childhood sexual abuse  (Clinician-administered Interview) | PTSD  (Davidson Trauma Scale) | **Avoidance coping**  (The Brief COPE - subscales) |
|  |  |  |  | **Behavioural disengagement**  (The Brief COPE - subscales) |
|  | **Espeleta et al (2021)** | Childhood maltreatment  (The Computer Assisted Maltreatment Inventory) | PTSD  (PTSD Symptom Scale) | **Attentional control**  (Attention Control Scale) |
|  |  |  |  | **Repetitive negative thinking styles**  (Perseverative Thinking Questionnaire) |
|  | **Goncharenko et al (2021)** | Childhood abuse  (The Childhood Trauma Questionnaire-Short Form) | PTSD  (The PTSD Checklist for DSM-5) | **Positive emotion regulation**  (The Difficulties in Emotion Regulation Scale Positive) |
|  | **Kim et al (2021)** | Childhood abuse  (Life Events Checklist) | PTSD  (The Clinician-Administered PTSD Scale (CAPS) for Diagnostic and Statistical Manual of Mental Disorders,  Fifth Edition (DSM-5)) | **Dysfunctional coping strategies**  (Brief COPE Inventory) |
|  | **Sistad et al (2021)** | Childhood maltreatment  (The Child Abuse and Trauma Scale) | PTSD  (The PTSD Checklist Civilian Version) | **Thought suppression**  (The White Bear Suppression Inventory) |
|  | **Watts et al (2021)** | Childhood emotional maltreatment  (Emotional Abuse subscale of the Childhood Trauma Questionnaire) | PTSD  (Short Post-Traumatic Stress Disorder Rating Interview) | **Intrusive rumination**  (The Event Related Rumination Inventory Intrusive Subscale) |
|  | **Fitzgerald (2022)** | Childhood maltreatment  (Childhood Trauma Questionnaire) | PTSD  (PTSD Checklist – Civilian Form) | **Mindfulness**  (Five Facet Mindfulness Questionnaire) |
|  | **Jernslett et al (2022)** | Childhood adversity  (The Adverse Childhood Experiences Questionnaire) | PTSD  (The Primary Care PTSD Screen for DSM-5) | **Coping strategies**  (The Brief Cope) |
|  | **Kiefer et al (2022)** | Childhood trauma  (The Childhood Trauma Questionnaire-Short Form) | PTSD  (The PTSD Checklist for DSM-5) | **Positive emotion regulation**  (The Responses to Positive Affect Scale) |
|  | **Maercker et al (2022)** | Childhood trauma and maltreatment  (Childhood Trauma Questionnaire German Version) | CPTSD  (The International Trauma Questionnaire) | **Trauma disclosure**  (The Disclosure of Trauma Questionnaire German Version) |
|  | **Feiler et al (2023)** | Adverse childhood experiences  (Adverse Childhood Experiences Questionnaire)  Benevolent childhood experiences  (Benevolent  Childhood Experiences Scale) | PTSD  (PTSD Checklist for the Diagnostic and Statistical Manual of  Mental Disorders Fifth Edition (DSM-5)) | **Emotion dysregulation**  (Difficulties in  Emotion Regulation Scale—Short Form) |
|  | **Fitzgerald & Lunt (2023)** | Childhood emotional abuse and neglect  (Childhood Trauma Questionnaire) | PTSD  (PTSD Checklist—  Civilian Form) | **Mindfulness**  (Five Facet Mindfulness  Questionnaire) |
|  | **Russo et al (2023)** | Childhood trauma exposure  (Traumatic Events Questionnaire) | PTSD  (PTSD Checklist for DSM-5) | **Emotion regulation**  (Difficulties in Emotion Regulation Scale) |
|  |  |  |  | **Distress intolerance**  (Distress Tolerance Scale) |
|  | **Békés et al (2023)** | Childhood trauma  (Early Trauma Inventory-Self-Report) | PTSD & CPTSD  (International Trauma Questionnaire) | **Shame**  (Experiences of Shame Scale) |
|  | **Haim-Nachum et al (2024)** | Childhood trauma  (The Childhood Trauma Questionnaire — Short Form) | PTSD  (Posttraumatic Stress Disorder Checklist) | **Anhedonia**  (The Snaith-Hamilton Pleasure Scale) |
| Interpersonal relationships | **Bradley et al (2005)** | Childhood maltreatment  (Childhood Trauma Questionnaire) | PTSD  (Davidson Trauma Scale) | **Social support**  (Multidimensional Profile of Social Support) |
|  | **Vranceanu et al (2007)** | Child multi-type maltreatment  (The Comprehensive Child Maltreatment Scale  (CCMS) for Adults) | PTSD  (The PTSD Symptom Scale-Interview) | **Social support**  (The Social Support Questionnaire) |
|  | **Stevens et al (2013)** | Childhood abuse  (The Childhood Trauma Questionnaire) | PTSD  (The PTSD Symptom Scale–Self Reported) | **Social support**  (The Social Supports Provision Scale) |
|  | **Murphy et al (2014)** | Childhood maltreatment  (Interview - several questions across four domains of childhood maltreatment: physical abuse, emotional abuse, neglect, and sexual abuse | PTSD  (the Primary Care PTSD Screen) | **Social support**  (The Crisis Support Scale) |
|  | **Wilson & Newins (2018)** | Childhood maltreatment  (Childhood Trauma Questionnaire Short Form) | PTSD  (PTSD Checklist for DSM-5) | **Anxiety sensitivity (social concerns)**  (18-item Anxiety Sensitivity Index-3 – subscale) |
|  | **Haj-Yahia et al (2019)** | Exposure to family violence in childhood  (The Conflict Tactics Scale – Revised) | PTSD  (The Briere and Runtz (1989) Trauma Symptom Checklist) | **Social support**  (The Provision of Social Relations (PSR) Scale) |
|  | **McLafferty et al (2019)** | Adverse childhood experiences  (Childhood Trauma Questionnaire question subset  & The Conflict Tactics Scale) | PTSD  (Structured Diagnostic Interview DSM-5 Criteria) | **Social network quality**  (Social Network Index) |
|  | **Eising et al (2021)** | Childhood maltreatment  (The Traumatic Experience Checklist German Version) | PTSD  (Diagnostic Interview for Mental Disorders) | **Social acknowledgement**  (German 16-item Social Acknowledgement Questionnaire) |
|  | **Lagdon et al (2021)** | Childhood maltreatment  (Items adopted from Christoffersen, Armour, Lasgaard, Andersen, and Elklit  (2013)) | PTSD  (PTSD Checklist for Diagnostic and Statistical Manual of Mental Disorders) | **Social support**  (The Multidimensional Scale of Perceived Social Support) |
|  | **Seitz et al (2021)** | Childhood trauma  (25 items from Childhood Trauma Questionnaire German Version) | PTSD  (PTSD Checklist for DSM-5 German Version) | **Social support**  (ENRICHD Social Support Inventory German Version) |
|  | **Maercker et al. (2022)** | Childhood trauma and maltreatment  (Childhood Trauma Questionnaire German Version) | CPTSD  (The International Trauma Questionnaire) | **Social acknowledgement**  (The German Social Acknowledgment Questionnaire) |
|  |  |  |  | **Social support**  (The German Social Support Questionnaire – Short Version) |
| Spiritual & religious beliefs | **Bradley et al (2005)** | Childhood maltreatment  (Childhood Trauma Questionnaire) | PTSD  (Davidson Trauma Scale) | **Religious coping**  (Brief Religious Coping Activities Scale) |
|  | **Zhang et al (2015)** | Childhood maltreatment  (The Childhood Trauma Questionnaire – Short Form) | PTSD  (Davidson Trauma Scale) | **Religious/existential wellbeing**  (The Spiritual Well-Being Scale) |
|  | **McCormick et al (2017)** | Adverse childhood experiences  (Adverse Childhood Experiences Scale) | PTSD  (Posttraumatic Stress Disorder Checklist - Civilian Version) | **Religious/spiritual struggles**  (Religious and Spiritual Struggles Scale) |
| Attachment style | **Simonelli and Sacchi (2016)** | Childhood traumatic experiences  (The Childhood Trauma Questionnaire - Short Form) | PTSD  (The Post-traumatic Stress Disorder Checklist for DSM-5) | **Romantic attachment style**  (The Experiences in Close Relationships Scale - Revised) |
|  | **Van Dijke et al (2018)** | Childhood trauma  (Traumatic Experiences Checklist) | CPTSD  (Dutch self-report version of the Structured Interview for Disorders of Extreme Stress Not Otherwise Specified) | **Adult relational fears**  (Relationship Style Questionnaire Dutch Version) |
|  | **Huang et al (2020)** | Childhood trauma  (Childhood Trauma Questionnaire) | PTSD  (The Posttraumatic Stress Disorder Checklist Scale) | **Attachment insecurity**  (The Experiences in Close Relationships - Revised) |
|  | **Maercker et al (2022)** | Childhood trauma and maltreatment  (Childhood Trauma Questionnaire German Version) | CPTSD  (The International Trauma Questionnaire) | **Attachment style**  (Experiences in Close Relationships – Revised Questionnaire German Version) |
|  | **Özdemir & Yazici (2025)** | Childhood maltreatment  (Childhood Trauma Questionnaire Short Form) | PTSD  (Posttraumatic Stress Disorder Checklist-5) | **Insecure attachment**  (Experiences in Close Relationships-Revised Scale) |
| Dissociation symptoms | **Van Dijke et al (2015)** | Childhood complex trauma  (Traumatic Experiences Checklist) | CPTSD  (Report Version of The Structured Interview for Disorders of Extreme Stress Not Otherwise Specified) | **Psychoform dissociation**  (The Dissociative Experiences Scale) |
|  | **Kratzer et al (2018)** | Childhood abuse  (The German version of the Childhood Trauma Questionnaire) | PTSD  (The German version of the Impact of Events Scale-R) | **Pathological dissociation**  (The Dissociative Experiences Scale German Version) |
|  | **Van Dijke et al (2018)** | Childhood trauma  (Traumatic Experiences Checklist) | CPTSD  (Dutch self-report version of the Structured Interview for Disorders of Extreme Stress Not Otherwise Specified) | **Psychoform dissociation**  (The Dissociative  Experiences Scale) |
|  |  |  |  | **Somatoform Dissociation**  (Somatoform Dissociation Questionnaire) |
|  | **Vang et al (2018)** | Childhood abuse and neglect  (The Childhood Trauma Questionnaire) | PTSD  (The Posttraumatic Checklist for DSM-5) | **Dissociation**  (The Dissociative Symptoms Scale - Brief) |

Table S4: AHRQ risk of bias assessment.

| **Study** | **Unbiased selection of cohort** | **Sample size calculation** | **Adequate description of cohort** | **Validated PTSD/CPTSD assessment tool** | **Missing data low or well handled** | **Analytic methods appropriate** | **Score** |
| --- | --- | --- | --- | --- | --- | --- | --- |
| **Schumm et al. (2004)** | Unclear | Unclear | Yes | Yes | Partial | Yes | **2** |
| **Bradley et al. (2005)** | Yes | Unclear | Yes | Yes | Unclear | Yes | **2** |
|  |  |  |  |  |  |  |  |
| **Street et al. (2005)** | Unclear | Unclear | Yes | Yes | Partial | Yes | **2** |
|  |  |  |  |  |  |  |  |
| **Vranceanu et al. (2007)** | Partial | Yes | Yes | Yes | Partial | Yes | **0** |
|  |  |  |  |  |  |  |  |
| **Stevens et al. (2013)** | Partial | Unclear | Yes | Yes | Unclear | Yes | **2** |
|  |  |  |  |  |  |  |  |
| **Weiss et al. (2013)** | Partial | Unclear | Yes | Yes | Unclear | Yes | **2** |
|  |  |  |  |  |  |  |  |
| **Lilly et al. (2014)** | No | Unclear | Yes | Yes | Unclear | Partial | **3** |
| **Murphy et al. (2014)** | Partial | Yes | Partial | Yes | Unclear | Partial | **1** |
|  |  |  |  |  |  |  |  |
| **Choi et al. (2015)** | Partial | Unclear | Yes | Yes | Unclear | Yes | **2** |
|  |  |  |  |  |  |  |  |
| **van Dijke et al. (2015)** | Partial | Yes | Yes | Yes | Yes | Yes | **0** |
|  |  |  |  |  |  |  |  |
| **Zhang et al. (2015)** | Partial | Unclear | Yes | Yes | Unclear | Yes | **2** |
|  |  |  |  |  |  |  |  |
| **Boughner et al. (2016)** | No | Yes | Partial | Yes | Unclear | Yes | **2** |
|  |  |  |  |  |  |  |  |
| **Simonelli and Sacchi (2016)** | No | Unclear | Partial | Yes | Unclear | Partial | **3** |
|  |  |  |  |  |  |  |  |
|  |  |  |  |  |  |  |  |
| **Kim et al. (2017)** | Partial | Yes | No | Yes | Partial | Yes | **1** |
|  |  |  |  |  |  |  |  |
| **McCormick et al. (2017)** | No | Yes | Yes | Yes | Yes | Partial | **1** |
|  |  |  |  |  |  |  |  |
| **Boykin et al. (2018)** | No | Unclear | Yes | Yes | Yes | Yes | **2** |
|  |  |  |  |  |  |  |  |
| **Kratzer et al. (2018)** | Partial | Yes | Yes | Yes | Unclear | Yes | **1** |
|  |  |  |  |  |  |  |  |
| **Menke et al. (2018)** | Partial | Unclear | Yes | Yes | Yes | Yes | **1** |
|  |  |  |  |  |  |  |  |
| **van Dijke et al. (2018b)** | Partial | Yes | Partial | Partial | Unclear | Yes | **1** |
|  |  |  |  |  |  |  |  |
| **Vang et al. (2018)** | Partial | Unclear | Yes | Yes | Unclear | Yes | **2** |
|  |  |  |  |  |  |  |  |
| **Wilson and Newins (2018)** | No | Unclear | Partial | Yes | Yes | Yes | **2** |
|  |  |  |  |  |  |  |  |
| **Cloitre et al. (2019)** | Yes | Unclear | Yes | Yes | Unclear | Yes | **2** |
|  |  |  |  |  |  |  |  |
| **Haj-Yahia et al. (2019)** | No | Yes | Partial | Yes | Unclear | Yes | **2** |
|  |  |  |  |  |  |  |  |
| **Hosseini Ramaghani et al. (2019)** | Yes | Yes | Partial | Yes | Unclear | Yes | **1** |
|  |  |  |  |  |  |  |  |
| **Ironson et al (2019)** | Yes | Unclear | Yes | Yes | Unclear | Yes | **2** |
| **Knefel et al. (2019)** | Partial | Unclear | Yes | Yes | Partial | Yes | **1** |
|  |  |  |  |  |  |  |  |
| **McLafferty et al. (2019)** | Partial | Yes | Yes | Yes | Yes | Partial | **0** |
|  |  |  |  |  |  |  |  |
| **Demir et al. (2020)** | Yes | Unclear | Yes | Yes | Unclear | Yes | **2** |
|  |  |  |  |  |  |  |  |
| **Huang et al. (2020)** | Partial | Unclear | Partial | Yes | Yes | Partial | **1** |
|  |  |  |  |  |  |  |  |
| **McCuaig Edge and Lee (2020)** | Partial | Yes | Partial | Yes | Yes | Yes | **0** |
| **Mitchell et al. (2020)** | Yes | Yes | Partial | Yes | Unclear | Yes | **1** |
|  |  |  |  |  |  |  |  |
| **Vasilopoulou et al. (2020)** | Partial | Partial | Yes | Yes | Unclear | Yes | **1** |
|  |  |  |  |  |  |  |  |
| **Batchelder et al. (2021)** | Yes | Unclear | Yes | Yes | Unclear | Partial | **2** |
|  |  |  |  |  |  |  |  |
| **Chen et al. (2021)** | Yes | Yes | Partial | Yes | Partial | Yes | **0** |
|  |  |  |  |  |  |  |  |
| **Chi et al. (2021)** | No | Yes | Partial | Partial | Partial | Yes | **1** |
|  |  |  |  |  |  |  |  |
| **Eising et al (2021)** | No | Yes | Yes | Yes | Yes | Yes | **1** |
| **Espeleta et al. (2021)** | No | Unclear | Partial | Yes | Partial | Yes | **2** |
|  |  |  |  |  |  |  |  |
| **Goncharenko et al. (2021)** | No | Yes | Partial | Yes | Unclear | Partial | **2** |
|  |  |  |  |  |  |  |  |
| **Guo et al. (2021b)** | No | Yes | Partial | Yes | Unclear | Yes | **2** |
|  |  |  |  |  |  |  |  |
| **Jowett et al. (2021)** | Yes | Yes | Yes | Yes | Unclear | Yes | **1** |
|  |  |  |  |  |  |  |  |
| **Kim et al. (2021)** | Yes | Unclear | Partial | Yes | Partial | Yes | **1** |
|  |  |  |  |  |  |  |  |
| **Lagdon et al. (2021)** | No | Yes | Partial | Yes | Yes | Yes | **2** |
|  |  |  |  |  |  |  |  |
| **Seitz et al. (2021)** | Partial | Unclear | Partial | Yes | Partial | Yes | **1** |
|  |  |  |  |  |  |  |  |
| **Sistad et al. (2021)** | No | Yes | Partial | Yes | Unclear | Yes | **2** |
|  |  |  |  |  |  |  |  |
| **Watts et al. (2021)** | No | Yes | Partial | Yes | Yes | Yes | **1** |
|  |  |  |  |  |  |  |  |
| **Alvarez et al. (2022)** | Yes | Unclear | Yes | Yes | Unclear | Yes | **2** |
|  |  |  |  |  |  |  |  |
| **Fitzgerald (2022)** | No | Yes | Partial | Yes | Unclear | Yes | **2** |
|  |  |  |  |  |  |  |  |
| **Jernslett et al. (2022)** | No | Yes | Partial | Yes | Yes | Partial | **1** |
|  |  |  |  |  |  |  |  |
| **Kiefer et al. (2022)** | No | Unclear | Partial | Yes | Yes | Yes | **2** |
|  |  |  |  |  |  |  |  |
| **Maercker et al. (2022)** | Partial | Unclear | Partial | Yes | Yes | Partial | **1** |
|  |  |  |  |  |  |  |  |
| **Annett et al (2023)** | Yes | Unclear | Yes | Yes | Partial | Yes | **1** |
|  |  |  |  |  |  |  |  |
| **Feiler et al. (2023)** | No | Yes | Partial | Yes | Unclear | Yes | **2** |
|  |  |  |  |  |  |  |  |
| **Fitzgerald & Lunt (2023)** | No | Unclear | Partial | Yes | Unclear | Yes | **3** |
| **Russo et al. (2023)** | No | Yes | Partial | Yes | Yes | Yes | **1** |
|  |  |  |  |  |  |  |  |
| **Békés et al (2023)** | Unclear | Unclear | Partial | Yes | Yes | Yes | **2** |
| **Li & Yiming Liang (2023)** | No | Yes | Partial | Yes | Unclear | Yes | **2** |
| **Darnell et al (2024)** | No | Unclear | Partial | Yes | Partial | Yes | **2** |
| **Haim-Nachum et al (2024)^1^** | No | Yes | Partial | Yes | Unclear | Partial | **2** |
| **Haim-Nachum et al (2024)^2^** | Partial | Yes | Yes | Yes | Unclear | Yes | **1** |
| **Kitano et al (2024)** | Partial | Unclear | Yes | Yes | Yes | Yes | **1** |
| **Arslan (2025)** | No | Unclear | Partial | Yes | Unclear | Yes | **2** |
| **Özdemir &** **Yazici (2025)** | No | Unclear | Yes | Yes | Yes | Yes | **1** |
| **Watts et al (2025)** | No | Yes | Yes | Yes | Yes | Yes | **1** |

Table S5: GRADE category assessment by category.

| Category | GRADE rating | Criteria |
| --- | --- | --- |
| Cognitive beliefs and appraisals  (n = 29) | Very low | Publication bias – 1 |
| Emotion regulation strategies  (n = 40) | Very low | Risk of bias – 1  Publication bias - 1 |
| Interpersonal relationships  (n = 7) | Very low | Publication bias – 1 |
| Spiritual and religious beliefs  (n = 2) | Very low | Risk of bias – 1  Inconsistency – 1  Imprecision – 1 |
| Attachment style  (n = 4) | Low | Publication bias - 1 |
| Dissociation symptoms  (n = 9) | Very low | Risk of bias – 1  Imprecision – 1  Publication bias – 1 |

Emotion regulation strategies, spiritual and religious beliefs, and dissociation symptoms were all downgraded for risk of bias, as most of its effects were assessed as having a moderate risk of bias using the AHRQ assessment. Cognitive beliefs and appraisals, interpersonal relationships, attachment style and dissociation symptoms were all downgraded for publication bias after inspection of funnel plots revealed obvious asymmetry (see Supplementary materials C). Spiritual and religious beliefs and dissociation symptoms were both downgraded for imprecision due to low sample size (> 400) combined with upper or lower confidence limits crossing the effect size of 0.5 in either direction. Lastly, spiritual and religious beliefs were downgraded for inconsistency due to wide variation in CSIE and overlapping confidence intervals (Ryan & Hill, 2016)

Table S6: GRADE meta-analysis assessment by category.

| Category | GRADE rating | Criteria |
| --- | --- | --- |
| Emotion regulation strategies  (n = 7) | Low | Inconsistency – 1  Publication bias - 1 |
| Resilience  (n = 3) | Very low | Indirectness – 1  Inconsistency - 1 |
| Maladaptive coping strategies  (n = 5) | Very low | Publication bias – 1  Publication bias - 1 |
| Mindfulness  (n = 9) | Very low | Risk of bias – 1  Indirectness – 1 |

All included outcomes for each meta-analysis category originated from non-randomised control trial studies (the majority of which were cross-sectional) and thus were rated low before downgrading or upgrading following GRADE criteria for study design quality (Balshem et al., 2011). Each category was downgraded at least once and thus received a very low-quality rating. Mindfulness was downgraded for risk of bias, as most of its effects were assessed as having a moderate risk of bias using the AHRQ assessment. Emotion regulation and resilience were downgraded for inconsistency due to high levels of heterogeneity. Emotion regulation and maladaptive coping strategies were downgraded for publication bias after inspection of funnel plots revealed obvious asymmetry (see Supplementary materials C). Resilience and mindfulness were downgraded for indirectness as most studies in this category did not use representative samples (majority used student populations).

**Category results**

**Cognitive beliefs and appraisals**

Table S7: Cognitive beliefs and appraisals category results.

| Study | N | Population | Predictor | Mediator(s) | Outcome | Effect size(s) | 95% CIs (lower, upper) | Effect type | Risk of bias | Forest plot ref |
| --- | --- | --- | --- | --- | --- | --- | --- | --- | --- | --- |
| Schumm et al (2005) * | 105 | Clinical | Child physical abuse | Interpersonal resource loss | PTSD | Z = .25 | (-0.00404, 0.01596) | R² | Moderate | - |
| Schumm et al (2005) * | 105 | Clinical | Child sexual abuse | Interpersonal resource loss | PTSD | Z = 1.22 | (0.01596, 0.40404) | R² | Moderate | - |
| Street et al (2005) * | 63 | Clinical | Potentially traumatic events in childhood | Trauma related guilt | PTSD | - | - | - | Moderate | - |
| Bradley et al (2005) * | 134 | Clinical | Childhood maltreatment | Self-esteem | PTSD | - | - | - | Moderate | - |
| Murphy et al (2014) | 2980 | General population | Childhood maltreatment | Self-esteem | PTSD | .000 | (- 0.001, 0.001) | CSIE | Low | Murphy (2014) |
| Kim et al (2017) | 169 | Community | Childhood maltreatment (abuse) | Resilience | PTSD | 0.01 | (-0.037519399, 0.057519399) | CSIE | Low | Kim (2017)a |
| Kim et al (2017) | 169 | Community | Childhood maltreatment (neglect) | Resilience | PTSD | 0.1 | (0.023908001, 0.176091999) | CSIE | Low | Kim (2017)b |
| Boykin et al (2018) | 288 | Trauma exposed students | Childhood maltreatment | Psychological inflexibility | PTSD | 0.06 | (0.024437148, 0.127345216) | CSIE | Moderate | Boykin (2018)a |
| Boykin et al (2018) | 288 | Trauma exposed students | Childhood maltreatment | Fear of self-compassion | PTSD | 0.03 | (-0.020590994, 0.094981238) | CSIE | Moderate | Boykin (2018)b |
| Menke et al (2018) | 100 | Community  6-month follow up | Childhood maltreatment | Maltreatment specific shame | PTSD | 0.19 | (0.10, 0.31) | CSIE | Low | Menke (2018)a |
| Menke et al (2018) | 100 | Community  12-month follow up | Childhood maltreatment | Maltreatment specific shame | PTSD | 0.19 | (0.10, 0.31) | CSIE | Low | Menke (2018)b |
| Menke et al (2018) | 100 | Community  15-month follow up | Childhood maltreatment | Maltreatment specific shame | PTSD | 0.17 | (0.07, 0.29) | CSIE | Low | Menke (2018)c |
| Menke et al (2018) | 100 | Community  18-month follow up | Childhood maltreatment | Maltreatment specific shame | PTSD | 0.09 | (0.07, 0.29) | CSIE | Low | Menke (2018)d |
| Wilson & Newins (2018) | 336 | Students | Childhood maltreatment | Anxiety sensitivity (cognitive concerns) | PTSD | 0.05 | (0.02, 0.09) | CSIE | Moderate | Wilson (2018) |
| Ironside et al (2019) | 290 | Clinical | Childhood sexual abuse with physical injury | Post-traumatic cognitions | PTSD | 0.104 | (0.036792615, 0.171207385) | CSIE | Moderate | Ironside (2019)a |
| Ironside et al (2019) | 290 | Clinical | Childhood sexual abuse with intense fear | Post-traumatic cognitions | PTSD | 0.104 | (0.035826087, 0.172173913) | CSIE | Moderate | Ironside (2019)b |
| Hosseini Ramaghani et al (2019) * | 432 | Clinical | Childhood trauma | Metacognition | PTSD | - | - | - | Low | - |
| Hosseini Ramaghani et al (2019) * | 432 | Clinical | Childhood trauma | Time perspectives | PTSD | - | - | - | Low | - |
| Huang et al (2020) | 296 | Clinical & Community | Childhood trauma | Mentalizing | PTSD | 0.1519 | (-0.0072, 0.319) | CSIE | Low | Huang (2020) |
| McCuaig Edge and Lee (2020) | 2927 | Community (Armed Forces) | Childhood neglect | Alexithymia | PTSD | −0.01 | [−0.006, [−0.057] | CSIE | Low | McCuaig Edge (2020)a |
| McCuaig Edge and Lee (2020) | 2927 | Community (Armed Forces) | Childhood adversity | Alexithymia | PTSD | 0.00 | [−0.006, 0.019] | CSIE | Low | McCuaig Edge (2020)b |
| Mitchell et al (2020) | 106 | Clinical | Childhood trauma | Trauma appraisals | PTSD | 0.30 | (0.18, 0.46) | CSIE | Low | Mitchell (2020) |
| Vasilopoulou et al (2020) | 42 | Clinical | Childhood trauma | Early maladaptive schemas | CPTSD | 0.39 | (0.08, 0.67) | CSIE | Low | Vasilopoulou (2020) |
| Chen et al (2021) | 108 | Clinical | Childhood maltreatment | Dispositional optimism/  pessimism | PTSD | 0.09 | (0.01, 0.19) | CSIE | Low | Chen (2021) |
| Chi et al (2021) | 2038 | Students | Childhood exposure to domestic violence | Self-compassion | PTSD | 0.008 | (0.002, 0.021) | CSIE | Low | Chi (2021)a |
| Chi et al (2021) | 2038 | Students | Childhood exposure to domestic violence | Resilience | PTSD | 0.001 | (-0.007,0.008) | CSIE | Low | Chi (2021)b |
| Guo et al (2021) | 1361 | Students | Adverse childhood experiences | Self-kindness | PTSD | 0.06 | (0.04, 0.09) | CSIE | Moderate | Guo (2021)a |
| Guo et al (2021) | 1361 | Students | Adverse childhood experiences | Self-judgement | PTSD | 0.03 | (0.02, 0.06) | CSIE | Moderate | Guo (2021)b |
| Guo et al (2021) | 1361 | Students | Adverse childhood experiences | Self-kindness | DSO | 0.08 | (0.05, 0.11) | CSIE | Moderate | Guo (2021)c |
| Guo et al (2021) | 1361 | Students | Adverse childhood experiences | Self-judgement | DSO | 0.05 | (0.03, 0.07) | CSIE | Moderate | Guo (2021)d |
| Kim et al (2021) | 212 | Community | Childhood abuse | Low resilience | PTSD | 0.1 | (0.0175, 0.231) | CSIE | Low | Kim (2021) |
| Watts et al (2021) | 396 | Students | Childhood emotional maltreatment | Event centrality | PTSD | 0.068 | (0.0288, 0.1072) | CSIE | Low | Watts (2021) |
| Li & Liang (2023) | 360 | Students | Childhood trauma | Self-esteem | PTSD | 0.05 | (0.02, 0.09) | CSIE | Moderate | Li (2023)a |
| Li & Liang (2023) | 360 | Students | Childhood trauma | Self-esteem | DSO | 0.16 | (0.11, 0.21) | CSIE | Moderate | Li (2023)b |
| Darnell et al (2024) * † | 203 | Students | Childhood stressful life events | Metacognition | PTSD | - | - | - | Moderate | - |
| Haim-Nachum et al (2024)^2^ | 230 | Community | Childhood maltreatment | Self-stigma | PTSD | 0.07 | (0.04, 0.10) | CSIE | Moderate | Haim-Nachum, Lazarov (2024) |
| Kitano et al (2024) | 752 | Community | Adverse childhood experiences | Self-compassion | PTSD | 0.04 | (0.02, 0.07) | CSIE | Low | Kitano (2024) |
| Arslan (2025) | 474 | Students | Adverse childhood experiences | Self-compassion | PTSD | 0.038 | (0.016, 0.07) | CSIE | Moderate | Arslan (2025)a |
| Arslan (2025) | 474 | Students | Adverse childhood experiences | Psychological flexibility | PTSD | 0.086 | (0.049, 0.124) | CSIE | Moderate | Arslan (2025)b |
| Watts et al (2025) | 263 | Students | Childhood psychological abuse | Posttraumatic appraisals | CPTSD | 0.7 | (0.6, 0.79) | CSIE | Low | Watts (2025) |

** Narrative inclusion only. † Non-significant results omitted from paper*

**Emotion regulation strategies**

Table S8: Emotion regulation strategies results.

| Study | N | Population | Predictor | Mediator(s) | Outcome | Effect size(s) | 95% CIs (lower, upper) | Effect type | Risk of bias | Forest plot ref |
| --- | --- | --- | --- | --- | --- | --- | --- | --- | --- | --- |
| Stevens et al (2013) | 139 | Community | Childhood abuse | Emotion regulation difficulties | PTSD | 0.15 | (0.02, 0.21) | CSIE | Moderate | Stevens (2013) |
| Weiss et al (2013) | 93 | Clinical | Childhood emotional abuse | Emotion dysregulation | PTSD | 0.02 | (0.003, 0.044) | CSIE | Moderate | Weiss (2013)a |
| Weiss et al (2013) | 93 | Clinical | Childhood physical abuse | Emotion dysregulation | PTSD | 0.014 | (0.001, 0.035) | CSIE | Moderate | Weiss (2013)b |
| Lilly et al (2014) | 99 | Community | Childhood trauma exposure | Emotion regulation | PTSD | 0.31 | (-0.00000569643, 0.6200) | CSIE | Moderate | Lilly (2014) |
| Choi et al (2015) | 84 | Clinical | Childhood trauma | Maladaptive coping | PTSD | 0.38 | (0.11, 0.64) | CSIE | Moderate | Choi (2015) |
| Boughner et al (2016) | 952 | General population | Childhood psychological trauma exposure | Mindfulness related traits | PTSD | 0.869 | (0.541, 1.219) | CSIE | Moderate | Boughner (2016) |
| Kratzer et al (2018) | 218 | Clinical | Childhood abuse | Mindfulness | PTSD | 0.1 | (-1.838, 0.2) | CSIE | Low | Kratzer (2018) |
| Van Dijke et al (2018) | 472 | Clinical | Childhood trauma | Affect dysregulation | CPTSD | 0.08 | (0.041749347, 0.109190601) | CSIE | Moderate | Van Dijke (2018) |
| Cloitre et al (2019) | 290 | Clinical trauma exposed | Adverse childhood experiences | Emotion regulation | PTSD | 0.14 | (0.08, 0.21) | CSIE | Moderate | Cloitre (2019) |
| Knefel et al (2019) † | 193 | Clinical | Childhood abuse | Emotion regulation: strategies | DSO | 0.10 | (0.04, 0.19) | CSIE | Low | Knefel (2019)a |
| Knefel et al (2019) † | 193 | Clinical | Childhood abuse | Emotion regulation: impulse | PTSD | 0.03 | (0.01, 0.08) | CSIE | Low | Knefel (2019)b |
| Knefel et al (2019) † | 193 | Clinical | Childhood abuse | Emotion regulation: impulse | DSO | 0.03 | (0.01, 0.08) | CSIE | Low | Knefel (2019)c |
| Knefel et al (2019) † | 193 | Clinical | Childhood neglect | Emotion regulation: clarity | DSO | 0.02 | (0.01, 0.07) | CSIE | Low | Knefel (2019)d |
| Knefel et al (2019) † | 193 | Clinical | Childhood abuse | Emotion regulation total | DSO | 0.15 | (0.05, 0.25) | CSIE | Low | Knefel (2019)e |
| Demir et al (2020) | 89 | Community (refugees) | Early adversity | Cognitive emotion regulation | PTSD | .007 | (0.003, 0.011) | CSIE | Moderate | Demir (2020) |
| Batchelder et al (2021) | 290 | Clinical | Childhood sexual abuse | Avoidance coping | PTSD | 0.06 | (0.019448276, 0.100551724) | CSIE | Moderate | Batchelder (2021)a |
| Batchelder et al (2021) | 290 | Clinical | Childhood sexual abuse | Behavioural disengagement | PTSD | 0.05 | (0.013432836, 0.086567164) | CSIE | Moderate | Batchelder (2021)b |
| Espeleta et al (2021) | 376 | Students | Childhood maltreatment | Attentional control | PTSD | 0.011 | (0.0003, 0.037) | CSIE | Moderate | Espeleta (2021)a |
| Espeleta et al (2021) | 376 | Students | Childhood maltreatment | Repetitive negative thinking styles | PTSD | 0.131 | (0.07, 0.20) | CSIE | Moderate | Espeleta (2021)b |
| Goncharenko et al (2021) | 465 | Community  (Trauma-exposed military veterans) | Childhood abuse | Positive emotion regulation | PTSD | 0.51 | (0.3924, 0.6276) | CSIE | Moderate | Goncharenko (2021) |
| Kim et al (2021) | 212 | Community | Childhood abuse | Dysfunctional coping strategies | PTSD | 0.1537 | (0.021, 0.3816) | CSIE | Low | Kim (2021) |
| Sistad et al (2021) | 586 | Students | Childhood maltreatment | Thought suppression | PTSD | 0.03 | (0.00, 0.05) | CSIE | Moderate | Sistad (2021) |
| Watts et al (2021) | 396 | Students | Childhood emotional maltreatment | Intrusive rumination | PTSD | 0.108 | (0.05704, 0.15896) | CSIE | Low | Watts (2021) |
| Fitzgerald (2022) | 177 | Students | Childhood maltreatment | Mindfulness | PTSD | 0.173 | (0.071, 0.287) | CSIE | Moderate | Fitzgerald (2022) |
| Jernslett et al (2022) | 898 | General population | Childhood adversity | Coping strategies | PTSD | 0.044937 | (0.02379, 0.081944) | CSIE | Low | Jernslett (2022) |
| Kiefer et al (2022) | 320 | Trauma exposed community | Childhood trauma | Positive emotion regulation: dampening | PTSD | 0.12 | (0.058, 0.165) | CSIE | Moderate | Kiefer (2022)a |
| Kiefer et al (2022) | 320 | Trauma exposed community | Childhood trauma | Positive emotion regulation: self-focused | PTSD | 0.002 | (-0.009, 0.019) | CSIE | Moderate | Kiefer (2022)b |
| Kiefer et al (2022) | 320 | Trauma exposed community | Childhood trauma | Positive emotion regulation: emotion-focused | PTSD | -0.001 | (-0.019, 0.0097) | CSIE | Moderate | Kiefer (2022)c |
| Maercker et al (2022) * | 126 | Community | Childhood trauma and maltreatment | Trauma disclosure | CPTSD | - | - | - | Low | - |
| Feiler et al (2023) | 1498 | Students | Adverse childhood experiences | Emotion dysregulation | PTSD | 1.49 | (1.20, 1.81) | CSIE | Moderate | Feiler (2023)a |
| Feiler et al (2023) * | 1498 | Students | Benevolent childhood experiences | Emotion dysregulation | PTSD | -2.31 | (-1.92, -2.73) | CSIE | Moderate | Feiler (2023)b |
| Fitzgerald & Lunt (2024) | 219 | Students | Emotional abuse | Mindfulness: non-judgement | PTSD | 0.255 | (0.144, 0.371) | CSIE | Moderate | Fitzgerald (2024)a |
| Fitzgerald & Lunt (2024) | 219 | Students | Emotional abuse | Mindfulness: non-reactivity | PTSD | -0.038 | (−0.083, −0.006) | CSIE | Moderate | Fitzgerald (2024)b |
| Fitzgerald & Lunt (2024) | 219 | Students | Emotional abuse | Mindfulness: acting with awareness | PTSD | 0.098 | (0.003, 0.207) | CSIE | Moderate | Fitzgerald (2024)c |
| Fitzgerald & Lunt (2024) | 219 | Students | Emotional neglect | Mindfulness: non-judgement | PTSD | -0.159 | (−0.278, −0.051) | CSIE | Moderate | Fitzgerald (2024)d |
| Fitzgerald & Lunt (2024) | 219 | Students | Emotional neglect | Mindfulness: nonreactivity | PTSD | 0.102 | (0.032, 0.186) | CSIE | Moderate | Fitzgerald (2024)e |
| Fitzgerald & Lunt (2024) | 219 | Students | Emotional neglect | Mindfulness: acting with awareness | PTSD | -0.052 | (-0.126, 0.002) | CSIE | Moderate | Fitzgerald (2024)f |
| Russo et al (2023) | 385 | Trauma exposed students | Childhood trauma exposure | Emotion regulation | PTSD | 0.76 | (0.38 ,1.25) | CSIE | Low | Russo (2023)a |
| Russo et al (2023) | 385 | Trauma exposed students | Childhood trauma exposure | Distress intolerance | PTSD | 0.19 | (0.05, 0.45) | CSIE | Low | Russo (2023)b |
| Békés et al (2023) | 226 | Clinical | Childhood trauma | Shame | PTSD | 0.412 | (0.090, 0.817) | CSIE | Moderate | Békés (2023)a |
| Békés et al (2023) | 226 | Clinical | Childhood trauma | Shame | DSO | 0.415 | (0.133, 0.758) | CSIE | Moderate | Békés (2023)b |
| Haim-Nachum et al (2024) | 230 | Trauma exposed community | Childhood trauma | Anhedonia | PTSD | 0.08 | (0.02 ,0.16) | CSIE | Moderate | Haim-Nachum, Amsalem (2024) |

** Narrative inclusion only. † Non-significant results omitted from paper*

**Meta-analysis**

Table S9: Studies for emotion regulation meta-analysis

| Study  *Key* | Predictor measure | Outcome measure | Mediator measure | CSIE (CI) | Suitable for inclusion? |
| --- | --- | --- | --- | --- | --- |
| Stevens et al (2013)  *Stevens (2013)* | The Childhood Trauma Questionnaire | The PTSD Symptom Scale–Self Reported | The Difficulties with Emotion Regulation Scale | 0.15 (0.02, 0.21) | Yes |
| Weiss et al (2013)  *Weiss (2013)a*  *(physical abuse)*  *Weiss (2013)b*  *(emotional abuse)* | The Childhood Trauma Questionnaire-Short Form: emotional & physical abuse subscales | The PTSD Checklist-Civilian Version | The Difficulties in Emotion Regulation Scale | Physical abuse: 0.02 (0.003, 0044)  Emotional abuse: 0.014 (0.001, 0.035) | Yes – sensitivity analysis for predictor subscales |
| Lilly et al (2014)  *Lilly (2014)* | The Childhood Trauma Questionnaire | The Posttraumatic Stress  Diagnostic Scale | The Difficulties in Emotion Regulation Scale | 0.31 (-0.00000569643, 0.62000) | Yes |
| Cloitre et al (2019)  *Cloitre (2019)* | 18-item Life Stressor Checklist | The Posttraumatic Stress  Disorder Checklist for DSM–5 | The Difficulties in  Emotional Regulation Scale | 0.14 (0.08, 0.21) | Yes |
| Knefel et al (2019) | The Childhood Trauma Questionnaire: abuse & neglect subscales | The International Trauma Questionnaire: PTSD & DSO | The Difficulties in  Emotional Regulation Scale: strategies, impulse, clarity and total scales | Abuse > strategies > DSO: 0.10 (0.04, 0.19)  Abuse > impulse > PTSD; 0.03 (0.01, 0.08)  Abuse > impulse > DSO: 0.03 (0.01, 0.08)  Neglect > clarity > DSO: 0.02 (0.01, 0.07)  Abuse > total > DSO: 0.15 (0.05, 0.25). | No: non-significant pathways not published, no PTSD total score available |
| Feiler et al (2023)  *Feiler (2023) (ACEs)* | Adverse Childhood Experiences Questionnaire  Benevolent Childhood Experiences Scale | PTSD Checklist for the Diagnostic and Statistical Manual of  Mental Disorders Fifth Edition (DSM-5) | Difficulties in  Emotion Regulation Scale—Short Form | ACEs: 1.49 (1.20, 1.81)  BCEs: -2.31 (-1.92, -2.73) | Yes: ACEs only, sensitivity analysis for short form mediator measure |
| Russo et al (2023)  *Russo (2023)* | Traumatic Events Questionnaire) | PTSD Checklist for DSM-5) | Difficulties in Emotion Regulation Scale | 0.76 (0.38, 1.25) | Yes |
| Total effects for analysis: 7 | | | | | |

Table S10: Studies for resilience meta-analysis

|  | Study | Predictor measure | Outcome measure | Mediator measure | CSIE (CI) | Suitable for inclusion? |
| --- | --- | --- | --- | --- | --- | --- |
|  | Kim et al (2017)  *Kim 1 (abuse)*  *Kim 2 (neglect)* | Childhood Trauma Questionnaire—Short Form Korean Version: abuse & neglect subscales | Impact of Event Scale —Revised – Korean Version | Connor – Davidson Resilience Scale Korean Version | Abuse: 0.01 (-0.037519399, 0.057519399)  Neglect: 0.1  (0.023908001, 0.176091999) | Yes |
|  | Chi et al (2021)  *Chi 1* | Revised Adverse  Childhood Experiences Questionnaire Chinese version | Abbreviated PTSD Checklist - Civilian version | Connor–Davidson Resilience  Scale Short Version | 0.001 (-0.007,0.008) | Yes – sensitivity analysis for short form mediator measure |
|  | Kim et al (2021)  *Kim 3* | Life Events Checklist | The Clinician-Administered PTSD Scale (CAPS) for Diagnostic and Statistical Manual of Mental Disorders,  Fifth Edition (DSM-5) | Connor-Davidson Resilience Scale | 0.1 (0.0175, 0.231) | Yes |
| Total effects for analysis: 4 | | | | | | |

Table S11: Studies for maladaptive coping meta-analysis

| Study | Predictor measure | Outcome measure | Mediator measure | CSIE (CI) | Suitable for inclusion? |
| --- | --- | --- | --- | --- | --- |
| Choi et al. (2015)  *Choi (2015)* | Childhood Trauma Questionnaire Short Form | Davidson Trauma Scale | The Brief COPE | 0.38 (0.11, 0.64) | Yes – sensitivity analysis for predictor short form measure |
| Batchelder et al (2021)  *Batchelder (2021)a*  *(coping)*  *Batchelder (2021)b*  *(disengagement)* | Clinician-administered Interview | Davidson Trauma Scale | The Brief COPE – subscales: maladaptive coping & behavioural disengagement | Coping: 0.06 (0.019448276, 0.100551724)  Disengagement: 0.05 (0.013432836, 0.086567164) | Yes – sensitivity analysis for mediator subscales |
| Kim et al (2021)  *Kim (2021)* | Life Events Checklist | The Clinician-Administered PTSD Scale (CAPS) for Diagnostic and Statistical Manual of Mental Disorders,  Fifth Edition (DSM-5) | Brief COPE Inventory | 0.1537 (0.021, 0.3816) | Yes |
| Jernslett et al (2022)  *Jernslett (2022)* | The Adverse Childhood Experiences Questionnaire | The Primary Care PTSD Screen for DSM-5 | The Brief COPE | 0.044937 (0.02379, 0.081944) | Yes |
| Total effects for analysis: 5 | | | | | |

Table S12: Studies for mindfulness meta-analysis

| Study | Predictor measure | Outcome measure | Mediator measure | CSIE (CI) | Suitable for inclusion? |
| --- | --- | --- | --- | --- | --- |
| Boughner et al (2016)  *Boughner 1* | Adverse Childhood Experiences Questionnaire | PTSD Checklist for DSM-5 | Five Facet Mindfulness Questionnaire–Short Form | 0.869 (0.541, 1.219) | Yes – sensitivity analysis for mediator short form measure |
| Kratzer et al. (2018)  *Kratzer 1* | The German version of the Childhood Trauma Questionnaire | The German version of the Impact of Events Scale-R | The Freiburg Mindfulness Inventory | 0.1 (-1.838, 0.2) | Maybe – mediator measures differ, sensitivity analysis required |
| Fitzgerald (2022)    *Fitzgerald 1* | Childhood Trauma Questionnaire | PTSD Checklist – Civilian Form | Five Facet Mindfulness Questionnaire | 0.173 (0.071, 0.287) | Yes |
| Fitzgerald & Lunt (2023)  *Fitzgerald 2 (abuse > non-judgement)*    *Fitzgerald 3 (abuse > non-reactivity)*  *Fitzgerald 4 (abuse > acting with awareness)*  *Fitzgerald 5 (neglect > non-judgement)*  *Fitzgerald 6 (neglect > non-reactivity)*  *Fitzgerald 7 (neglect > acting with awareness)* | Childhood Trauma Questionnaire: abuse and neglect subscales | PTSD Checklist—  Civilian Form | Five Facet Mindfulness  Questionnaire: subscales | Abuse > non-judgement > PTSD: 0.255 (0.144, 0.371)  Abuse > non-reactivity > PTSD: -0.038 (−0.083, −0.006)  Abuse > acting with awareness > PTSD: 0.098 (0.003, 0.207)  Neglect > non-judgement > PTSD:  -0.159 (−0.278, −0.051)  Neglect > non-reactivity > PTSD: 0.102 (0.032, 0.186)  Neglect > acting with awareness > PTSD: -0.052 (-0.126, 0.002) | Yes – sensitivity analysis for multiple subscales |
| Total effects for analysis: 9 | | | | | |

**Meta-analysis results**

**Emotion regulation**

Table S13: Emotion regulation meta-analysis results

| Model | No of effects | Effect size | SE | CI | p/Rhat | I^2^/bulk ESS | H^2^/tail ESS |
| --- | --- | --- | --- | --- | --- | --- | --- |
| Bayesian (fixed effects gaussian) | 7 | 0.03 | 0.01 | 0.02, 0.04 | Rhat: 1.00 | Bulk ESS: 983 | Tail ESS: 891 |
| Frequentist (fixed effects) | 7 | 0.027 | 0.007 | 0.015, 0.04 | p < 0.0001 | I^2^: 95.13% | T^2^: 20.54 |
| Frequentist (random effects REML) | 7 | 0.392 | 0.2 | 0.001, 0.784 | p = 0.0496 | I^2^: 99.83% | T^2^: 573.48 |
| Sensitivity analysis 1* | 6 | 0.46 | 0.227 | 0.015, 0.904 | p = 0.0426 | I^2^: 99.36% | T^2^: 156.75 |
| Sensitivity analysis 2* | 6 | 0.134 | 0.058 | 0.019, 0.248 | p = 0.022 | I^2^: 97.47% | T^2^: 39.54 |

**Sensitivity analysis 1: Weiss 1 removed. Sensitivity analysis 2: Feiler 1 removed.*

Figure S1: Posterior distribution of pooled effect (Bayesian)

Random effects models were re-estimated for sensitivity checks. With Weiss 1 removed (due to predictor subscales used), the pooled effect size slightly increased (b: 0.46, SE: 0.23, CI 0.02, 0.90, p = 0.043), with heterogeneity remaining high (I2: 99.36%). When Feiler 1 was removed (due to short form mediator measure used), a smaller pooled effect was generated (b: 0.13, SE: 0.06, CI 0.02, 0.25, p = 0.022) alongside a decrease in between-study variance (T2: 0.015) and slightly decreased heterogeneity (I2: 97.47%). These findings suggest that while the effect of emotion regulation as a mediator between ACEs and PTSD is a robust small effect, the strength and heterogeneity is influenced by individual studies.

**Maladaptive coping**

Table S14: Maladaptive coping meta-analysis results

| Model | No of effects | Effect size | SE | CI | p/Rhat | I^2^/bulk ESS | H^2^/tail ESS |
| --- | --- | --- | --- | --- | --- | --- | --- |
| Bayesian (fixed effects gaussian) | 5 | 0.05 | 0.01 | 0.03, 0.07 | Rhat: 1.00 | Bulk ESS: 1045 | Tail ESS: 829 |
| Frequentist (fixed effects) | 5 | 0.053 | 0.01 | 0.033, 0.073 | p < 0.0001 | 46.55% | 1.87 |
| Frequentist (random effects REML) | 5 | 0.053 | 0.01 | 0.033, 0.073 | p < 0.0001 | 0.23% | 1.00 |
| Sensitivity analysis 1* | 4 | 0.051 | 0.01 | 0.03, 007 | p < 0.0001 | 1.18% | 1.01 |
| Sensitivity analysis 2* | 4 | 0.054 | 0.01 | 0.03, 0.078 | p < 0.0001 | 0.14% | 1.00 |

**Sensitivity analysis 1: Choi 1 removed. Sensitivity analysis 2: Batchelder 2 removed.*

Figure S2: Posterior distribution of pooled effect (Bayesian)

Random effects models were re-estimated twice for sensitivity checks. With Choi 1 removed (due to predictor short form measure used), the model shows similar effect size and significance level to the full models (b: 0.05, SE: 0.01, CI: 0.03, 0.07, p < 0.0001) indicating a strong consistent effect. Heterogeneity was very low (I2: 1.18%, Q(3) = 1.6, p = 0.658). With Batchelder 2 removed (due to mediator subscales used), the findings were again very consistent with the other models (b: 0.05, SE: 0.01, CI: 0.03, 0.07, p < 0.0001) with minimal heterogeneity (I2: 0.14%, Q(3) = 7.4, p = 0.06). Overall, the findings across this category demonstrate a consistent, stable, and robust, small effect of maladaptive coping as a mediator between ACEs and PTSD.

**Category results continued**

**Interpersonal relationships**

This category contains 7 effect sizes from 6 studies. 5 studies considering interpersonal relationship mediators were unable to have CSIE’s calculated. The included samples consisted of clinically recruited outpatients (n = 234), community (including community and online community recruitment, veterans and those affected by compulsory social measures and placement in Switzerland) (n = 3680), undergraduate university students (n = 1492) and a national Danish general population sample (n = 2980). Only one study considered CPTSD (of which CSIEs could not be obtained) (Maercker et al., 2022) compared to those which considered PTSD as the outcome (n = 10). All studies had small effect sizes with mostly narrow confidence intervals. Risk of bias across studies was mostly low, and the category was downgraded in the GRADE assessment for publication bias, thus the overall quality of evidence was very low, and effect sizes should be interpreted with caution.

Most mediators reached significance, including social support, anxiety sensitivity (social concerns) and social acknowledgement. However, Bradley et al. (2005) did not test social support as a mediator between childhood maltreatment and PTSD as the Baron and Kenny (1986b) recommendations for mediation analysis were not met (the relationship between social support and PTSD was no longer significant when child abuse was entered into the regression predicting PTSD). More recent studies in this category however used modern mediation analysis approaches such as Hayes PROCESS, and consistently found significant mediation effects from social support (albeit with small effect sizes) (Haj-Yahia et al., 2019; Lagdon et al., 2021; Murphy et al., 2014; Seitz et al., 2021; Stevens et al., 2013). The only non-significant effect was found by McLafferty et al. (2019), who report social network quantity did not mediate the relationship between household dysfunction in childhood and PTSD. The largest overall effect size in this category was reported by Vranceanu et al. (2007), who demonstrated social support mediated the relationship between child multi-type maltreatment and PTSD (CSIE: 0.26 [0.06597, 0.4540]), however confidence intervals were wide, and overall all effect sizes were small. See Table S14 for full results and Figure S3 for forest plot.

Table S14: Interpersonal relationships category results.

| Study | N | Population | Predictor | Mediator(s) | Outcome | Effect size(s) | 95% CIs (lower, upper) | Effect type | Risk of bias | Forest plot ref |
| --- | --- | --- | --- | --- | --- | --- | --- | --- | --- | --- |
| Bradley et al (2005)* | 134 | Clinical | Childhood maltreatment | Social support | PTSD | - | - | - | Moderate | - |
| Vranceanu et al (2007) | 100 | Clinical | Child multi-type maltreatment | Social support | PTSD | 0.26 | (0.06597, 0.4540) | CSIE | Low | Vranceanu 1 |
| Stevens et al (2013) | 139 | Community | Childhood abuse | Social support | PTSD | 0.05 | (0.01, 0.20) | CSIE | Moderate | Stevens 2 |
| Murphy et al (2014) | 2980 | General population | Childhood maltreatment | Social support | PTSD | 0.066 | (0.044, 0.087) | CSIE | Low | Murphy 2 |
| Wilson & Newins (2018) | 336 | Students | Childhood maltreatment | Anxiety sensitivity (social concerns) | PTSD | 0.03 | (0.01, 0.06) | CSIE | Moderate | Wilson 2 |
| Haj-Yahia et al (2019) | 516 | Students | Experiencing interparental violence | Social support | PTSD | 0.04 | (0.007, 0.07) | CSIE | Moderate | Haj-Yahia 1 |
| Haj-Yahia et al (2019) | 516 | Students | Witnessing interparental violence | Social support | PTSD | 0.02 | (0.008, 0.06) | CSIE | Moderate | Haj-Yahia 2 |
| McLafferty et al (2019)* | 3092 | Community (veterans) | Adverse childhood experiences | Social network quality | PTSD | - | - | OR | Low | - |
| Eising et al (2021)* | 238 | Community | Childhood maltreatment | Social acknowledgement | PTSD | - | - | - | Low | - |
| Lagdon et al (2021)* | 640 | Students | Childhood maltreatment | Social support | PTSD | - | - | OR | Moderate | - |
| Seitz et al (2021) | 85 | Community | Childhood trauma | Social support | PTSD | 0.101 | (0.013, 0.209) | CSIE | Low | Seitz 1 |
| Maercker et al (2022)* | 126 | Community | Childhood trauma and maltreatment | Social acknowledgement | CPTSD | - | - | - | Low | - |
| Maercker et al (2022)* | 126 | Community | Childhood trauma and maltreatment | Social support | CPTSD | - | - | - | Low | - |

** Narrative inclusion only. † Non-significant results omitted from paper*

Figure S3: Forest plot for interpersonal relationships category results PTSD

**Spiritual and religious beliefs**

This small category contains results from two studies. It was not possible to calculate CSIEs for one study. The included samples comprised of clinical (both inpatient and outpatient; n = 326) and undergraduate university students (n = 458). Of which, all considered PTSD as the outcome (n = 3). Two studies had a moderate risk of bias (Bradley et al., 2005; Zhang et al., 2015) and one study had a low risk of bias (McCormick et al., 2017). The category overall was downgraded three times in the GRADE assessment for risk of bias, inconsistency, and imprecision which resulted in a rating of very low quality. Thus, all effect sizes should be interpreted with caution.

Negative religious coping (no CSIE available), existential well-being and religious meaning struggles were found to be significant mediators. The only non-significant finding was from Zhang et al. (2015), who found that religious well-being did not mediate the relationship between childhood maltreatment and PTSD, however the CSIE for this pathway was not reported and it was not possible to be calculated. Where the CSIE was available or possible to be calculated, all effect sizes were observed to be very small with moderate to wide confidence intervals suggesting lower precision. See Table S15 for full results and Figure S4 for forest plot.

Table S15: Spiritual and religious beliefs category results.

| Study | N | Population | | Predictor | Mediator(s) | Outcome | Effect size(s) | 95% CIs (lower, upper) | Effect type | Risk of bias | Forest plot ref |
| --- | --- | --- | --- | --- | --- | --- | --- | --- | --- | --- | --- |
| Bradley et al (2005)* | 134 | Clinical | Childhood maltreatment | | Religious coping | PTSD | - | - | - | Moderate | - |
| Zhang et al (2015)*†* | 192 | Clinical | Childhood maltreatment | | Existential wellbeing | PTSD | 0.049 | (0.001, 0.114) | CSIE | Moderate | Zhang 1 |
| McCormick et al (2017) | 458 | Students | Adverse childhood experiences | | Religious/spiritual struggles | PTSD | 0.051898 | (-0.7, 0.138) | CSIE | Low | McCormick 1 |

** Narrative inclusion only. † Non-significant results omitted from paper*

Figure S4: Forest plot for spiritual and religious beliefs category results PTSD

**Attachment style**

This category contains five effect sizes from three studies. It was not possible to calculate CSIEs for two studies. The included samples comprised of clinical outpatients (n = 472), undergraduate university students (n = 327), community recruited and those affected by compulsory social measures and placement in Switzerland (n = 492), and a mixed clinical/community sample (n = 296). Two studies considered CPTSD as the outcome and three studies considered PTSD. Most studies in this category received a low risk of bias rating, and this category was downgraded for publication bias in the GRADE quality assessment. All effect sizes should therefore be interpreted with some caution.

All reported mediator pathways bar one (CA > attachment avoidance > PTSD; this study used Sobel tests to determine significance) (Özdemir & Yazici, 2025) were significant, including relational fears (both fear of abandonment and fear of closeness), attachment insecurity, and romantic attachment (on some subscale pathways only; CSIE unable to be calculated or extracted and non-significant pathways not reported) (Simonelli & Sacchi, 2016). The largest effect size in this category was reported by Huang et al. (2020), who considered attachment insecurity as a mediator between childhood trauma and PTSD (CSIE: 0.2244 [0.0555, 0.5056]). The effect size is verging on moderate, however confidence intervals are wide, reducing precision. All other effect sizes were small. See Table S16 for full results and Figure S5a and S5b for forest plots.

Table S16: Attachment style category results.

| Study | N | Population | Predictor | Mediator(s) | Outcome | Effect size(s) | 95% CIs (lower, upper) | Effect type | Risk of bias | Forest plot ref |
| --- | --- | --- | --- | --- | --- | --- | --- | --- | --- | --- |
| Simonelli and Sacchi (2016)*† | 327 | Students | Childhood traumatic experiences | Romantic attachment style | PTSD | - | - | - | Moderate | - |
| Van Dijke et al (2018) | 472 | Clinical | Childhood trauma | Adult relational fears: fear of abandonment | CPTSD | 0.03 | (0.016057441, 0.07386423) | CSIE | Low | Van Dijke 2 |
| Van Dijke et al (2018) | 472 | Clinical | Childhood trauma | Adult relational fears: fear of closeness | CPTSD | 0.04 | (0.009634465, 0.0545953) | CSIE | Low | Van Dijke 3 |
| Huang et al (2020) | 296 | Clinical and community | Childhood trauma | Attachment insecurity | PTSD | 0.2244 | (0.0555, 0.5056) | CSIE | Low | Huang 2 |
| Maercker et al (2022)* | 126 | Community | Childhood trauma and maltreatment | Attachment style | CPTSD | - | - | - | Low | - |
| Özdemir & Yazici (2025) | 366 | Community | Childhood maltreatment | Attachment anxiety | PTSD | 0.09 | (0.0293, 0.175) | CSIE |  | Özdemir 1 |
| Özdemir & Yazici (2025) | 366 | Community | Childhood maltreatment | Attachment avoidance | PTSD | 0.04 | (0.0022, 0.111) | CSIE |  | Özdemir 2 |

** Narrative inclusion only. † Non-significant results omitted from paper*

Figure S5a: Forest plot for attachment style category PTSD

Figure S5b: Forest plot for attachment style category PTSD

**Dissociation symptoms**

This category contains nine effect sizes from four studies. This is the only category consisting of all clinically recruited participants (both inpatient and outpatient; n = 1239). Two studies considered CPTSD as the outcome, and two studies considered PTSD. Most studies in this category received a low risk of bias rating, however more CSIE outcomes originated from studies with moderate risk of bias. This category was therefore marked down in the GRADE assessment for risk of bias, alongside imprecision and publication bias, and thus received an overall quality rating of very low. All effect sizes should therefore be interpreted with caution.

Half of included pathways showed significant mediation effects, including dissociation (from sexual and emotional abuse subscales), psychoform dissociation, and pathological dissociation. van Dijke et al. (2018) found that positive somatoform dissociation did not mediate the relationship between childhood trauma and CPTSD, however the reported standardised beta effect size was out with the calculated standardised CIs (CSIE: 0.08 [0.00, 0.029] and was therefore excluded from the forest plot diagram due to possible error. Vang et al. (2018) found dissociation did not mediate the relationship between childhood physical abuse, physical neglect, emotional neglect and PTSD, with all non-significant pathways reported in full. The strongest evidence in this category is seen in Vang et al. (2018), where childhood sexual abuse (CSIE: 0.225 [0.106, 0.344]) and childhood emotional abuse (CSIE: 0.297 [0.158, 0.437]) are both mediated by dissociation in their relationship to PTSD. These effect sizes are verging on moderate, and confidence intervals for both effects are moderately narrow, indicating some precision. Overall, all effect sizes were small. See Table S17 for full results and Figure S6a and S6b for forest plots.

Table S17: Dissociation symptoms category results.

| Study | N | Population | Predictor | Mediator(s) | Outcome | Effect size(s) | 95% CIs (lower, upper) | Effect type | Risk of bias | Forest plot ref |
| --- | --- | --- | --- | --- | --- | --- | --- | --- | --- | --- |
| Van Dijke et al (2015) | 450 | Clinical | Childhood complex trauma | Psychoform dissociation | CPTSD | 0.115549772 | (0.06214704, 0.178321405) | CSIE | Low | Van Dijke 4 |
| Kratzer et al (2018) | 218 | Clinical | Childhood abuse | Pathological dissociation | PTSD | 0.14 | (-2.573, 0.28) | CSIE | Low | Kratzer 2 |
| Van Dijke et al (2018) | 472 | Clinical | Childhood trauma | Psychoform dissociation | CPTSD | 0.05 | (0.01926893, 0.077075718) | CSIE | Low | Van Dijke 5 |
| Van Dijke et al (2018)* | 472 | Clinical | Childhood trauma | Somatoform dissociation | CPTSD | 0.08 | (0.00, 0.028903394) | CSIE | Low | - |
| Vang et al (2018) | 99 | Clinical | Childhood emotional abuse | Dissociation | PTSD | 0.297 | (0.158, 0.437) | CSIE | Moderate | Vang 1 |
| Vang et al (2018) | 99 | Clinical | Childhood physical abuse | Dissociation | PTSD | -0.100 | (-0.243, 0.044) | CSIE | Moderate | Vang 2 |
| Vang et al (2018) | 99 | Clinical | Childhood sexual abuse | Dissociation | PTSD | 0.225 | (0.106, 0.344) | CSIE | Moderate | Vang 3 |
| Vang et al (2018) | 99 | Clinical | Childhood emotional neglect | Dissociation | PTSD | 0.102 | (-0.054, 0.258) | CSIE | Moderate | Vang 4 |
| Vang et al (2018) | 99 | Clinical | Childhood physical neglect | Dissociation | PTSD | 0.010 | (-0.147, 0.168) | CSIE | Moderate | Vang 5 |

** Narrative inclusion only. † Non-significant results omitted from paper*

Figure S6a: Forest plot for dissociation symptoms category results PTSD

Figure S6b: Forest plot for dissociation symptoms category results CPTSD

**Meta-analysis results continued**

**Resilience**

This category contains four effects from three studies, and a total sample size of n = 2419. A Bayesian fixed effects meta-analysis with weakly informative priors was conducted to estimate the overall pooled effect size. Results showed no meaningful or consistent effect, with no effect size and credible intervals including 0 (posterior mean: 0.00, CI: -0.00, 0.01). The model demonstrated strong convergence (Rhat: 1.00) and effective sample sizes for bulk and tail estimates were high (Bulk ESS: 1085, Tail ESS: 945). Results from a frequentist fixed effects metanalysis aligned with the Bayesian model with a very small non-significant effect size (b: 0.003, SE: 0.004, CI: -0.005, 0.01, p = 487) and moderate heterogeneity (I^2^: 69.26%, Q(3) = 9.76, p = 0.021) suggesting a random effects model would be a more appropriate fit. A random effects model was conducted using REML and found a slightly larger but non-significant effect (b: 0.038, SE: 0.026, CI -0.0132, 0.089, p = 0.146) with moderate heterogeneity (I^2^: 75.99%, T^2^: 0.0018).

Sensitivity checks were run twice (see supplementary materials Table 7), with random effects models re-estimated. With Kim 2 removed, there was no meaningful change in pooled effect size or significance (b: 0.002, SE: 0.004, CI: -0.006, 0.009, p = 0.654), however heterogeneity dropped substantially (I^2^: 0%). With Chi 1 removed, a larger non-significant effect size was observed (b: 0.06, SE: 0.034, CI: -0.006, 0.127, p = 0.077) with moderate heterogeneity (I^2^: 59.2%). Overall, the above models do not support resilience as a mediator in the ACEs > PTSD pathway. See Table S18 for full results.

Table S18: Resilience meta-analysis results

| Model | No of effects | Effect size | SE | CI | p/Rhat | I^2^/bulk ESS | H^2^/tail ESS |
| --- | --- | --- | --- | --- | --- | --- | --- |
| Bayesian (fixed effects gaussian) | 4 | 0.00 | 0.00 | -0.00, 0.01 | Rhat: 1.00 | Bulk ESS: 1085 | Tail ESS: 945 |
| Frequentist (fixed effects) | 4 | 0.003 | 0.004 | -0.005, 0.01 | p = 0.487 | 69.26% | 3.25 |
| Frequentist (random effects REML) | 4 | 0.038 | 0.023 | -0.013, 0.089 | p = 0.146 | 75.99% | 4.16 |
| Sensitivity analysis 1* | 3 | 0.002 | 0.004 | -0.006, 0.009 | p = 0.448 | 0.03% | 1.00 |
| Sensitivity analysis 2* | 3 | 0.06 | 0.03 | -0.006, 0.127 | p = 0.077 | 59.19% | 2.45 |

**Sensitivity analysis 1: Kim 2 removed. Sensitivity analysis 2: Chi 1 removed.*

**Mindfulness**

This category contains nine effects from four studies, and a total sample size of n = 1566. A Bayesian fixed effects meta-analysis with weakly informative priors was conducted to estimate the overall pooled effect size. Results showed a small pooled effect size with credible intervals including 0 (posterior mean: 0.01, SE: 0.01, CI: -0.01, 0.04) indicating no clear evidence of a consistent or true effect. The model showed strong convergence (Rhat: 1.00), and effective sample sizes for bulk and tail estimates were high (Bulk ESS: 920, Tail ESS: 916). Results from a frequentist fixed effects meta-analysis aligned with the Bayesian model, with a very small non-significant pooled effect size (b: 0.01, SE: 0.01, CI: -0.01, 0.04, p = 0.345) and high significant heterogeneity (I^2^: 89.71%, Q(8) = 77.74, p < 0.0001). Thus, a random effects model was conducted using REML and closely aligned with the other models, finding a small non-significant effect (b: 0.01, SE: 0.08, CI: -0.046, 0.297, p = 0.153) with significant high heterogeneity (I^2^: 96.67, Q(8) = 77.74, p < 0.0001).

Sensitivity checks were run three times (see supplementary materials Table 9), with random effects models re-estimated. With Boughner et al (2016; Broughner 1) removed, the model shows similar effect size and significance level to the full models (b: 0.05, SE: 0.05, CI: -0.05, 0.153, p = 0.319) and heterogeneity remained significant and high (I2: 90.49%, Q(7) = 53.08, p < 0.0001). Similar findings were found with Kratzer et al. (2018; Kratzer 1) removed (b: 0.13, SE: 0.09, CI: -0.05, 0.31, p = 0.162; I2: 97.21%, Q(7) = 77.712, p < 0.0001). Finally, with Fitzgerald 3, 4, 5, 6, and 7 removed (leaving only Fitzgerald 2 from this study), a significant pooled effect was found (b: 0.372, SE: 0.178, CI: 0.023, 0.722, p = 0.037). Heterogeneity however remained significant and high (I2: 91.96%, Q(3) = 14.83, p = 0.002). These findings suggest that this study cluster has a large influence on the main models, however despite its significance when removed in sensitivity analysis, it has very high heterogeneity and wide CIs indicating low precision. The evidence for this mediator effect in the ACEs > PTSD pathway is therefore inconsistent. See Table S19 for full results.

Table S19: Mindfulness meta-analysis results

| Model | No of effects | Effect size | SE | CI | p/Rhat | I^2^/bulk ESS | H^2^/tail ESS |
| --- | --- | --- | --- | --- | --- | --- | --- |
| Bayesian (fixed effects gaussian) | 9 | 0.01 | 0.01 | -0.01, 0.04 | Rhat: 1.00 | Bulk ESS: 920 | Tail ESS: 916 |
| Frequentist (fixed effects) | 9 | 0.013 | 0.014 | -0.014, 0.039 | p = 0.345 | 89.71% | 9.72 |
| Frequentist (random effects REML) | 9 | 0.0126 | 0.088 | -0.046, 0.297 | p = 0.1526 | 96.67% | 30.01 |
| Sensitivity analysis 1* | 8 | 0.052 | 0.052 | -0.05, 0.154 | p = 0.319 | 90.49% | 10.52 |
| Sensitivity analysis 2* | 8 | 0.127 | 0.091 | -0.05, 0.305 | p = 0.162 | 97.21% | 35.81 |
| Sensitivity analysis 3* | 4 | 0.0372 | 0.178 | 0.023, 0.722 | p = 0.0368 | 91.96% | 12.44 |

**Sensitivity analysis 1: Broughner 1 removed. Sensitivity analysis 2: Kratzer 1 removed. Sensitivity analysis 3: Fitzgerald 3, 4, 5, 6, and 7 removed*

**Supplementary materials 3**

Table S20: Excluded studies after full text consideration

| **Study** | **Reason for exclusion** |
| --- | --- |
| Afifi et al (2009) | No mediation analysis |
| Alpay et al (2017) | No direct PTSD/CPTSD outcome |
| Arpino et al (2022) | No mediation analysis |
| Augsburger et al (2017) | Incorrect pathways |
| Bachar et al (2015) | No mediation analysis |
| Barlow et al (2017) | Outcome measure not appropriate |
| Berman et al (2021) | No mediation analysis |
| Berthelot et al (2019) | No direct PTSD/CPTSD outcome |
| Bradley et al (2011) | No mediation analysis |
| Bulut (2018) | No mediation analysis |
| Busuito et al (2014) | No mediation analysis |
| Cantón-Cortés et al (2011) | Full text not in English |
| Chung & Freh (2019) | No childhood predictor |
| Clint & Fessler (2022) | No mediation analysis |
| Cloitre et al (2005) | No mediation analysis |
| Crow et al (2021) | No mediation analysis |
| Daniels et al (2012) | No direct PTSD/CPTSD outcome |
| Doba et al (2022) | Participants aged < 18 |
| Dorahy et al (2015) | No mediation analysis |
| Ensink et al (2023) | No childhood predictor |
| Fani et al (2010) | No full outcome measure used |
| Fasciano et al (2021) | No mediation analysis |
| Flynn et al (2023) | No mediation analysis |
| Gambaro et al (2020) | Incorrect pathways |
| Gilbar et al (2020) | Incorrect pathways |
| Gluck et al (2017) | No mediation analysis |
| Gobin et al (2013) | No psychological mediator |
| Goldstine-Cole (2021) | Thesis (not peer reviewed) |
| Guyon-Harris et al (2017) | No mediation analysis |
| Haim-Nachum et al (2022) | No mediation analysis |
| Hirai et al (2020) | No childhood predictor |
| Hood & Carter (2008) | No mediation analysis |
| Huang et al (2023) | No mediation analysis |
| Kalmakis et al (2020) | No mediation analysis |
| Kamen et al (2013) | No mediation analysis |
| Kampling et al (2022) | Participants aged < 18 |
| Kim et al (2018) | No mediation analysis |
| Krammer et al (2016) | No full outcome measure used |
| Krause-Utz et al (2023) | No mediation analysis |
| Kulkarni et al (2013) | No mediation analysis |
| Lee et al (2020) | Incorrect pathways |
| Lee et al (2020) | No mediation analysis |
| Lee et al (2022) | Incorrect pathways |
| Liu et al (2020) | No childhood predictor |
| MacKinnon et al (2018) | No mediation analysis |
| Mccutchen et al (2023) | No mediation analysis |
| McKeever et al (2006) | No mediation analysis |
| Meyer et al (2019) | No mediation analysis |
| Mirhashem et al (2017) | Incorrect pathways |
| Molsa et al (2017) | No mediation analysis |
| Narayan et al (2017) | No mediation analysis |
| Nguyen et al (2023) | Incorrect pathways |
| Nobakht et al (2019) | No mediation analysis |
| Opaas & Varvin (2015) | No mediation analysis |
| Ortigo et al (2013) | No childhood predictor |
| Patock-Peckham et al (2020) | Incorrect pathways |
| Reinert et al (2016) | No mediation analysis |
| Ross & Kearney (2015) | Participants aged < 18 |
| Roth et al (2022) | No psychological mediator |
| Russo et al (2022) | No mediation analysis |
| Sciarrino et al (2020) | No mediation analysis |
| Sexton et al (2015) | No mediation analysis |
| Shenk et al (2012) | Participants aged < 18 |
| Shenk et al (2014) | Participants aged < 18 |
| Simons et al (2017) | Incorrect pathways |
| Steele et al (2017) | Incorrect pathways |
| Stein et al (2005) | No mediation analysis |
| Swopes et al (2013) | Incorrect pathways |
| Thomas et al (2025) | No psychological mediator (behavioural) |
| Thome et al (2018) | No mediation analysis |
| Tran et al (2013) | No mediation analysis |
| Van Buren & Weierich (2015) | No childhood predictor |
| Veith et al (2017) | Incorrect pathways |
| Walker et al (2022) | No mediation analysis |
| Walker et al (2023) | No direct PTSD/CPTSD outcome |
| Wiseman et al (2021) | Incorrect pathways |
| Wrenn et al (2011) | No mediation analysis |
| Yang et al (2022) | Incorrect pathways |
| Zerach et al (2025) | Incorrect pathways |

**Details of CSIE calculations per study**

Where not noted otherwise, the authors were contacted in the first instance for the CSIE, standardised confidence intervals, and any other clarifications required. Where this was not available, they were asked for any missing pathway information which would make the calculations simpler to conduct.

All calculations were conducted in excel.

**Schumm et al (2004)**

Unable to be calculated. Authors provided Sobel Z ab, but did not report CI's, SE, SDs for x and y variables, or p values for Z.

Note: authors were not able to be contacted to provide further pathway information

**Bradley et al (2005)**

Unable to be calculated. Authors provided Sobel Z ab, but did not report CI's, SE, SDs for x and y variables, or p values for Z.

Note: authors were not able to be contacted to provide further pathway information

**Street et al (2005)**

Unable to be calculated. Authors provided standardised ab, but did not report CI's, SE for c pathways, or p values, so CIs could not be calculated.

Note: authors were not able to be contacted to provide further pathway information.

**Vranceauna et al (2007)**

Provided: standardised structural coefficients and standardised ab.

Calculations: to calculate confidence intervals, firstly the ab was unstandardised using std coefficient/(SDx/SDy). T values for ab were then calculated using =TINV(p-value, df). The unstandardised ab was then divided by t to calculate SE. Confidence intervals could then be calculated using the following: upper CI = ab + 1.96*SEab, lower CI = ab – 1.96*SEab. Finally, the confidence intervals could both be standardised using the following: upper 95% CI = unstd upper CI x (SDx/SDy), lower 95% CI = unstd lower CI x (SDx/SDy).

Note: authors were not able to be contacted to provide further pathway information

**Weiss et al (2013)**

Provided: unstandardised pathway coefficients, unstandardised ab, unstandardised CI’s.

Calculations: ab was standardised using StdB = b x (SDx/SDy) and confidence intervals were standardised using the following: upper 95% CI = unstd upper CI x (SDx/SDy), lower 95% CI = unstd lower CI x (SDx/SDy).

Note: authors were not able to be contacted to provide further pathway information

**Lilly et al (2014)**

Provided: Z coefficients, standardised ab.

Calculations: to calculate confidence intervals in the absence of an overall x scale SD, SE values were derived by computing Z values: Z values were calculated using =ABS(NORMSINV(p-value/2)), SE ab value was then computed using SE = standardised beta/Z. Finally, CIs were calculated using the following: upper CI = Std B + (1.96*SE), lower CI = Std B – (1.96*SE).

**van Dijke et al (2015)**

Provided: Unstandardised pathway coefficients

Calculations: ab was standardised using StdB = b x (SDx/SDy) and confidence intervals were standardised using the following: upper 95% CI = unstd upper CI x (SDx/SDy), lower 95% CI = unstd lower CI x (SDx/SDy).

**Simonelli & Sacchi (2016)**

Unable to calculate CI’s. Authors did not provide CI’s, p values, or c pathways. Non-significant pathways not reported.

**Kim et al (2016)**

In this paper there was a discrepancy between the pathway figures provided in the text for the childhood abuse subscale and the figures reported in Table 3 - Direct, Indirect (Through Resilience), and Total Effects of Childhood Maltreatment on Post-traumatic Stress Symptoms in Korean Marine Conscript.

The text description states: “higher scores of childhood abuse were associated with higher scores of PTSS during military service, but the relationship was not mediated by resilience (regression coefﬁcient = .01, p = .68). However, resilience mediated the relationship between childhood neglect and PTSS during military service (total effect = -.10). In the SEM model, childhood neglect had no signiﬁcant direct effect on PTSS, but resilience had a signiﬁcant positive buffering effect on the indirect relationship between childhood neglect and PTSS, for a signiﬁcant total effect (see Table 3)”

The table reports the figures as follows:

Childhood abuse direct effect = .49**, indirect effect = .01**, total effect = .49**.

Childhood neglect direct effect = -.20, indirect effect = .10*, total effect = -.10*.

Due to the higher likelihood that the table figures were copied directly from analysis output, these were the figures used in this study. It is however important to acknowledge the possibilities of inaccuracies with the utilised figures.

Provided: beta coefficients, standardised ab.

Calculations: to calculate confidence intervals in the absence of overall score SDs for x, SE values were derived by computing Z values: Z values were calculated using =ABS(NORMSINV(p-value/2)), SE ab value was then computed using SE = standardised beta/Z. Finally, CIs were calculated using the following: upper CI = Std B + (1.96*SE), lower CI = Std B – (1.96*SE).

**Wesley et al (2017)**

Provided: standardised beta coefficients, standardised CI’s, Z ab.

Calculations: to calculate the CSIE, the provided standardised a and b pathways were multiplied together.

**Boykin et al (2018)**

Provided: beta coefficients, standardised ab, unstandardised CIs.

Calculations: confidence intervals were standardised using the following: upper 95% CI = unstd upper CI x (SDx/SDy), lower 95% CI = unstd lower CI x (SDx/SDy).

**Kratzer et al (2018)**

Provided: beta coefficients, standardised ab, unstandardised CIs.

Calculations: in order to calculate the standardised CIs in the absence of the overall x score SD, SE values were derived by computing Z values: Z values were calculated using =ABS(NORMSINV(p-value/2)). SE ab value was then computed using SE = standardised beta/Z. Finally, CIs were calculated using the following: upper CI = Std B + (1.96*SE), lower CI = Std B – (1.96*SE).

**Van Dijke et al (2018)**

Provided: unstandardised CIs, standardised ab

Confidence intervals were standardised using the following: upper 95% CI = unstd upper CI x (SDx/SDy), lower 95% CI = unstd lower CI x (SDx/SDy).

**Wilson et al (2018)**

Provided: unstandardised pathway coefficients, unstandardised ab, unstandardised CIs

Calculations: ab was standardised using StdB = b x (SDx/SDy) and confidence intervals were standardised using the following: upper 95% CI = unstd upper CI x (SDx/SDy), lower 95% CI = unstd lower CI x (SDx/SDy).

**Hosseini Ramaghani et al (2019)**

Unable to be calculated. Authors did not report ab for desired pathways, CI's, p values or c pathways.

**Ironson et al (2019)**

Provided: standardised regression coefficients, Z Sobel ab, standardised CIs.

Calculations: the CSIE was computed using ab = std c – std c’. In order to calculate CIs in the absence of x and y SDs, SE values were derived using Z values. SE ab value was computed using SE = standardised beta/Z. Finally, CIs were calculated using the following: upper CI = Std B + (1.96*SE), lower CI = Std B – (1.96*SE).

**McLafferty et al (2019)**

Odds Ratios unable to be converted.

**Haj-Yahia et al (2019)**

Provided: unstandardised regression coefficients, unstandardised ab, unstandardised CIs.

Calculations: ab was standardised using StdB = b x (SDx/SDy) and confidence intervals were standardised using the following: upper 95% CI = unstd upper CI x (SDx/SDy), lower 95% CI = unstd lower CI x (SDx/SDy).

**Huang et al (2020)**

Provided: standardised pathway coefficients, sequential ab pathways, standardised CIs.

Calculations: in order to establish the non-sequential CSIE of each individual mediator, the provided standardised a and b pathways were multiplied together. The same method was applied to calculate the CIs.

**Lagdon et al (2021)**

Odds Ratios unable to be converted.

**Batchelder et al (2021)**

Provided: standardised regression coefficients, Sobel Z score

Calculations: the CSIE was computed using ab = std c – std c’. In order to calculate CIs, SE values were derived using Z values. SE ab value was computed using SE = standardised beta/Z. Finally, CIs were calculated using the following: upper CI = Std B + (1.96*SE), lower CI = Std B – (1.96*SE).

**Eising et al (2021)**

Unable to be calculated. ab reported pathways are unstandardised, missing SD values for x and y. No ab for non-significant effects. No ab p values. No c, no SE. No M and SD for PTSD. Some pathways are reported but not all, and these are all unstandardised.

**Chen et al (2021)**

Provided: unstandardised regression coefficients, unstandardised ab, unstandardised CIs.

Calculations: ab was standardised using StdB = b x (SDx/SDy) and confidence intervals were standardised using the following: upper 95% CI = unstd upper CI x (SDx/SDy), lower 95% CI = unstd lower CI x (SDx/SDy).

**Espeleta et al (2021)**

Provided: unstandardised regression coefficients, unstandardised ab, unstandardised CIs.

Calculations: ab was standardised using StdB = b x (SDx/SDy) and confidence intervals were standardised using the following: upper 95% CI = unstd upper CI x (SDx/SDy), lower 95% CI = unstd lower CI x (SDx/SDy).

**Goncharenko et al (2021)**

Provided: beta coefficients, standardised ab.

Calculations: confidence intervals were calculated using the following: upper CI = ab + 1.96*SEab, lower CI = ab – 1.96*SEab.

**Kim et al (2021)**

Provided: beta coefficients, sequential ab.

Calculations: in order to establish the non-sequential CSIE of each individual mediator, the provided standardised a and b pathways were multiplied together. The same method was applied to calculate the CIs.

**Watts et al (2021)**

Provided: beta coefficients, standardised ab

Calculations: confidence intervals were calculated using the following: upper CI = ab + 1.96*SEab, lower CI = ab – 1.96*SEab.

**Jernslett et al (2022)**

Provided: unstandardised coefficients, unstandardised ab, unstandardised CIs.

Calculations: ab was standardised using StdB = b x (SDx/SDy) and confidence intervals were standardised using the following: upper 95% CI = unstd upper CI x (SDx/SDy), lower 95% CI = unstd lower CI x (SDx/SDy).

**Maercker et al (2022)**

Unable to be calculated. Authors did not report any ab paths, SE, c pathways or SDs for x and y.

**Kiefer et al (2023)**

Provided: unstandardised pathway coefficients, unstandardised ab, unstandardised CIs.

Calculations: ab was standardised using StdB = b x (SDx/SDy) and confidence intervals were standardised using the following: upper 95% CI = unstd upper CI x (SDx/SDy), lower 95% CI = unstd lower CI x (SDx/SDy).

**Annett et al (2023)**

Provided: unstandardised estimates, standardised ab

Calculations: in order to calculate CIs, T-values were first calculated using =TINV(p-value, df). SEab could then be calculated by dividing ab by t. Then, CIs were calculated using the following: upper CI = Std B + (1.96*SE), lower CI = Std B – (1.96*SE). They were standardised using the following: upper 95% CI = unstd upper CI x (SDx/SDy), lower 95% CI = unstd lower CI x (SDx/SDy).

**Darnell et al (2024)**

Unable to be calculated. Authors did not report nonsignificant pathways, ab, CI for ab, p values for ab, SE’s, c or c'.

**Arslan (2025)**

Provided: unstandardised estimates

Calculations: ab was standardised using StdB = b x (SDx/SDy) and confidence intervals were standardised using the following: upper 95% CI = unstd upper CI x (SDx/SDy), lower 95% CI = unstd lower CI x (SDx/SDy).

**Özdemir &** **Yazici (2025)**

Provided: beta coefficients, sequential ab.

Calculations: in order to establish the non-sequential CSIE of each individual mediator, the provided standardised a and b pathways were multiplied together. The same method was applied to calculate the CIs.

**Systematic category funnel plots with mean effect size**

**Meta-analysis category funnel plots with pooled effect size**

**References (* for included in review)**

Aafjes-van Doorn, K., Kamsteeg, C., & Silberschatz, G. (2020). Cognitive mediators of the relationship between adverse childhood experiences and adult psychopathology: A systematic review. *Dev Psychopathol*, *32*(3), 1017-1029. https://doi.org/10.1017/s0954579419001317

Agapova, M., Devine, E. B., Nguyen, H., Wolf, F. M., & Inoue, L. Y. (2014). Using indirect comparisons to compare interventions within a Cochrane review: a tool for comparative effectiveness research. *J Comp Eff Res*, *3*(4), 345-357. https://doi.org/10.2217/cer.14.29

*Annett, J., Tillson, M., Walker, M., Webster, J. M., & Staton, M. (2023). Adverse childhood experiences and mental health among incarcerated women: Self-esteem as a mediating mechanism. *Child Abuse & Neglect*, *146*, 106486. <https://doi.org/https://doi.org/10.1016/j.chiabu.2023.106486>

de Arellano, M. A., Lyman, D. R., Jobe-Shields, L., George, P., Dougherty, R. H., Daniels, A. S., Ghose, S. S., Huang, L., & Delphin-Rittmon, M. E. (2014). *Trauma-focused cognitive-behavioral therapy for children and adolescents: assessing the evidence*. Psychiatric services (Washington, D.C.), 65(5), 591–602. https://doi.org/10.1176/appi.ps.201300255

*Arslan, G., Özdemir Bişkin, S., & Kocaayan, F. (2025). Adverse childhood experiences, self-compassion, psychological flexibility, and posttraumatic stress disorder. *Children and Youth Services Review*, *169*, 108109. https://doi.org/https://doi.org/10.1016/j.childyouth.2024.108109

Association, A. P. (1980). *Diagnostic and statistical manual of mental disorders. (3rd ed.).*

Balshem, H., Helfand, M., Schünemann, H. J., Oxman, A. D., Kunz, R., Brozek, J., Vist, G. E., Falck-Ytter, Y., Meerpohl, J., Norris, S., & Guyatt, G. H. (2011). GRADE guidelines: 3. Rating the quality of evidence. *Journal of Clinical Epidemiology*, *64*(4), 401-406. https://doi.org/10.1016/j.jclinepi.2010.07.015

*Barlow, M. R., Goldsmith Turow, R. E., & Gerhart, J. (2017). Trauma appraisals, emotion regulation difficulties, and self-compassion predict posttraumatic stress symptoms following childhood abuse. *Child Abuse Negl*, *65*, 37-47. https://doi.org/10.1016/j.chiabu.2017.01.006

Baron, R. M., & Kenny, D. A. (1986a). The moderator-mediator variable distinction in social psychological research: conceptual, strategic, and statistical considerations. *J Pers Soc Psychol*, *51*(6), 1173-1182. https://doi.org/10.1037//0022-3514.51.6.1173

*Batchelder, A. W., Safren, S. A., Coleman, J. N., Boroughs, M. S., Thiim, A., Ironson, G. H., Shipherd, J. C., & O'Cleirigh, C. (2021). Indirect Effects From Childhood Sexual Abuse Severity to PTSD: The Role of Avoidance Coping. *J Interpers Violence*, *36*(9-10), NP5476-NP5495. https://doi.org/10.1177/0886260518801030

Bernstein, D. P., Fink, L., Handelsman, L., & Foote, J. (1998). Childhood trauma questionnaire. *Assessment of family violence: A handbook for researchers and practitioners.*

Bethell, C., Jones, J., Gombojav, N., Linkenbach, J., & Sege, R. (2019). Positive Childhood Experiences and Adult Mental and Relational Health in a Statewide Sample: Associations Across Adverse Childhood Experiences Levels. *JAMA Pediatrics*, *173*(11), e193007-e193007. https://doi.org/10.1001/jamapediatrics.2019.3007

Bick, J., & Nelson, C. A. (2016). Early Adverse Experiences and the Developing Brain. *Neuropsychopharmacology*, *41*(1), 177-196. https://doi.org/10.1038/npp.2015.252

Bing-Canar, H., Stocks, J. K., Khan, H., Rauch, A. A., Obolsky, M. A., Lapitan-Moore, F., Phillips, M. S., Soble, J. R., Pliskin, N. H., Song, W., & Resch, Z. J. (2024). Adverse childhood experiences, cognitive functioning, depression, and anxiety in adulthood. *Psychol Trauma*, *16*(7), 1139-1148. https://doi.org/10.1037/tra0001637

Boals, A., & Murrell, A. R. (2016). I Am > Trauma: Experimentally Reducing Event Centrality and PTSD Symptoms in a Clinical Trial*. Journal of Loss and Trauma*, 21(6), 471–483. https://doi.org/10.1080/15325024.2015.1117930

*Boughner, E., Thornley, E., Kharlas, D., & Frewen, P. (2016). Mindfulness-related traits partially mediate the association between lifetime and childhood trauma exposure and PTSD and dissociative symptoms in a community sample assessed online*. Mindfulness*, 7(3), 672-679. https://doi.org/10.1007/s12671-016-0502-3

*Boykin, D. M., Himmerich, S. J., Pinciotti, C. M., Miller, L. M., Miron, L. R., & Orcutt, H. K. (2018). Barriers to self-compassion for female survivors of childhood maltreatment: The roles of fear of self-compassion and psychological inflexibility. *Child Abuse & Neglect*, *76*, 216-224. https://doi.org/10.1016/j.chiabu.2017.11.003

*Bradley, R., Schwartz, A. C., & Kaslow, N. J. (2005). Posttraumatic Stress Disorder Symptoms Among Low-Income, African American Women With a History of Intimate Partner Violence and Suicidal Behaviors: Self-Esteem, Social Support, and Religious Coping. *Journal of Traumatic Stress*, *18*(6), 685-696. https://doi.org/10.1002/jts.20077

Brewin, C. R. (2011). The Nature and Significance of Memory Disturbance in Posttraumatic Stress Disorder. *Annual Review of Clinical Psychology*, *7*(Volume 7, 2011), 203-227. https://doi.org/https://doi.org/10.1146/annurev-clinpsy-032210-104544

Burnette, C. B., Luzier, J. L., Bennett, B. L., Weisenmuller, C. M., Kerr, P., Martin, S., Keener, J., & Calderwood, L. (2022). Concerns and recommendations for using Amazon MTurk for eating disorder research. *Int J Eat Disord*, *55*(2), 263-272. https://doi.org/10.1002/eat.23614

Burns, E. E., Jackson, J. L., & Harding, H. G. (2010). Child Maltreatment, Emotion Regulation, and Posttraumatic Stress: The Impact of Emotional Abuse. *Journal of Aggression, Maltreatment & Trauma*, *19*(8), 801-819. https://doi.org/10.1080/10926771.2010.522947

Cashin, A. G., & Lee, H. (2021). An introduction to mediation analyses of randomized controlled trials. *Journal of Clinical Epidemiology*, *133*, 161-164. https://doi.org/https://doi.org/10.1016/j.jclinepi.2021.02.014

*Chen, J., Christ, N. M., Shih, C.-H., Xie, H., Grider, S. R., Lewis, C., Elhai, J. D., & Wang, X. (2021). Dispositional optimism mediates relations between childhood maltreatment and PTSD symptom severity among trauma-exposed adults. *Child Abuse & Neglect*, *115*. https://doi.org/10.1016/j.chiabu.2021.105023

*Chi, X. L., Huang, Q. M., Liu, X. F., Huang, L. Y., Hu, M. J., Chen, Z. J., Jiao, C., Stubbs, B., Hossain, M. M., & Zou, L. Y. (2021). Self-compassion and resilience mediate the relationship between childhood exposure to domestic violence and posttraumatic growth/stress disorder during COVID-19 pandemic. *World Journal of Psychiatry*, *11*(11), 1106-1115. https://doi.org/10.5498/wjp.v11.i11.1106

Choi, H., Lee, W., & Hyland, P. (2021). Factor structure and symptom classes of ICD-11 complex posttraumatic stress disorder in a South Korean general population sample with adverse childhood experiences. *Child Abuse &amp; Neglect*, *114*, 104982. https://doi.org/10.1016/j.chiabu.2021.104982

*Choi, K. W., Sikkema, K. J., Velloza, J., Marais, A., Jose, C., Stein, D. J., Watt, M. H., & Joska, J. A. (2015). Maladaptive coping mediates the influence of childhood trauma on depression and PTSD among pregnant women in South Africa. *Archives of Women's Mental Health*, *18*(5), 731-738. https://doi.org/10.1007/s00737-015-0501-8

*Cloitre, M., Khan, C., Mackintosh, M.-A., Garvert, D. W., Henn-Haase, C. M., Falvey, E. C., & Saito, J. (2019). Emotion regulation mediates the relationship between ACES and physical and mental health. *Psychological trauma: theory, research, practice, and policy*, *11*(1), 82-89. https://doi.org/10.1037/tra0000374

Cole, E., & Diaz, A. (2024). Specific emotion regulation deficits differentiate and mediate the relationship between adverse childhood experiences and internalizing psychopathology. *Journal of Affective Disorders Reports*, *16*, 100722. https://doi.org/https://doi.org/10.1016/j.jadr.2024.100722

Contractor, A. A., Armour, C., Shea, M. T., Mota, N., & Pietrzak, R. H. (2016). Latent profiles of DSM-5 PTSD symptoms and the “Big Five” personality traits. *Journal of Anxiety Disorders*, *37*, 10-20. <https://doi.org/https://doi.org/10.1016/j.janxdis.2015.10.005>

Contractor, A. A., Banducci, A. N., & Weiss, N. H. (2022). Critical considerations for the positive memory-posttraumatic stress disorder model. *Clin Psychol Psychother*, *29*(1), 81-91. <https://doi.org/10.1002/cpp.2599>

Cortés-García L, Takkouche B, Rodriguez-Cano R, Senra C. Mediational mechanisms involved in the relation between attachment insecurity and depression: A meta-analysis. J Affect Disord. 2020 Dec 1;277:706-726. doi: 10.1016/j.jad.2020.08.082. Epub 2020 Sep 2. PMID: 32911221.

Crandall, A., Miller, J. R., Cheung, A., Novilla, L. K., Glade, R., Novilla, M. L. B., Magnusson, B. M., Leavitt, B. L., Barnes, M. D., & Hanson, C. L. (2019). ACEs and counter-ACEs: How positive and negative childhood experiences influence adult health. *Child Abuse & Neglect*, *96*, 104089. https://doi.org/https://doi.org/10.1016/j.chiabu.2019.104089

Crouch, E., Strompolis, M., Radcliff, E., & Srivastav, A. (2018). Examining exposure to adverse childhood experiences and later outcomes of poor physical and mental health among South Carolina adults. *Children and Youth Services Review*, *84*, 193-197. https://doi.org/https://doi.org/10.1016/j.childyouth.2017.11.031

*Darnell, B. C., Lee, S. R., Despotes, A. M., Jones, D. A., & Valentiner, D. P. (2024). Metacognition as a Mediator for Posttraumatic Stress Symptoms Following Childhood Stressful Life Events: An Examination of the Construct Validity of the Metacognitions Questionnaire-Posttraumatic Stress Disorder. *Psychological Reports*. https://doi.org/https://doi.org/10.1177/00332941231217467

*Demir, Z., Böge, K., Fan, Y., Hartling, C., Harb, M., Hahn, E., Seybold, J., & Bajbouj, M. (2020). The role of emotion regulation as a mediator between early life stress and posttraumatic stress disorder, depression and anxiety in Syrian refugees. *Translational Psychiatry*, *10*(1). https://doi.org/https://doi.org/10.1038/s41398-020-01062-3

Dunn, E. C., Soare, T. W., Zhu, Y., Simpkin, A. J., Suderman, M. J., Klengel, T., Smith, A., Ressler, K. J., & Relton, C. L. (2019). Sensitive Periods for the Effect of Childhood Adversity on DNA Methylation: Results From a Prospective, Longitudinal Study. *Biol Psychiatry*, *85*(10), 838-849. https://doi.org/10.1016/j.biopsych.2018.12.023

Ehlers, A., & Clark, D. M. (2000). A cognitive model of posttraumatic stress disorder. *Behav Res Ther*, *38*(4), 319-345. https://doi.org/10.1016/s0005-7967(99)00123-0

*Eising, C. M., Voelkle, M. C., Rohner, S. L., Maercker, A., & Thoma, M. V. (2021). Lifetime post-traumatic stress disorder in older individuals with a history of institutional upbringing in childhood: The role of social acknowledgement and stressful life events. *European Journal of Psychotraumatology*, *12*(1). https://doi.org/10.1080/20008198.2021.1915578

*Espeleta, H. C., Taylor, D. L., Kraft, J. D., & Grant, D. M. (2021). Child maltreatment and cognitive vulnerabilities: Examining the link to posttraumatic stress symptoms. *Journal of American College Health*, *69*(7), 759-766. https://doi.org/10.1080/07448481.2019.1706538

*Feiler, T., Vanacore, S., & Dolbier, C. (2023). Relationships Among Adverse and Benevolent Childhood Experiences, Emotion Dysregulation, and Psychopathology Symptoms. *Advers Resil Sci*, 1-17. https://doi.org/10.1007/s42844-023-00094-0

Felitti, V. J., Anda, R. F., Nordenberg, D., Williamson, D. F., Spitz, A. M., Edwards, V., Koss, M. P., & Marks, J. S. (1998). Relationship of childhood abuse and household dysfunction to many of the leading causes of death in adults. The Adverse Childhood Experiences (ACE) Study. *Am J Prev Med*, *14*(4), 245-258. https://doi.org/10.1016/s0749-3797(98)00017-8

*Fitzgerald, M. (2022). Childhood maltreatment is associated with adult psychopathology through decreased dispositional mindfulness. *Journal of Aggression, Maltreatment & Trauma*. https://doi.org/10.1080/10926771.2022.2043971

*Fitzgerald, M., & Lunt, K. (2024). What Aspects of Dispositional Mindfulness Mediate the Association Between Childhood Emotional Maltreatment and Posttraumatic Stress Disorder? *Traumatology*, *Advance online publication.* https://doi.org/https://dx.doi.org/10.1037/trm0000476

Goncharenko, S., Forkus, S. R., Contractor, A. A., Kiefer, R., & Weiss, N. H. (2021). The role of positive emotion dysregulation in the relationship between childhood abuse and PTSD in a community sample of veterans. *Child Abuse & Neglect*, *114*, Article 104979. https://doi.org/10.1016/j.chiabu.2021.104979

Gopalakrishnan, S., & Ganeshkumar, P. (2013). Systematic Reviews and Meta-analysis: Understanding the Best Evidence in Primary Healthcare. *J Family Med Prim Care*, *2*(1), 9-14. https://doi.org/10.4103/2249-4863.109934

Greene, T., Contractor, A. A., Dicker-Oren, S. D., Fentem, A., & Sznitman, S. R. (2024). The Effects of the Processing of Positive Memories Technique on Posttrauma Affect and Cognitions Among Survivors of Trauma: Protocol for a Daily Diary Study. *JMIR Res Protoc*, *13*, e51838. https://doi.org/10.2196/51838

Gruhn, M. A., & Compas, B. E. (2020). Effects of maltreatment on coping and emotion regulation in childhood and adolescence: A meta-analytic review. *Child Abuse & Neglect*, *103*, 104446. https://doi.org/https://doi.org/10.1016/j.chiabu.2020.104446

*Guo, T., Huang, L., Hall, D. L., Jiao, C., Chen, S.-T., Yu, Q., Yeung, A., Chi, X., & Zou, L. (2021a). The relationship between childhood adversities and complex posttraumatic stress symptoms: a multiple mediation model. *European Journal of Psychotraumatology*, *12*(1), 1936921. https://doi.org/10.1080/20008198.2021.1936921

Guyatt, G. H., Oxman, A. D., Vist, G. E., Kunz, R., Falck-Ytter, Y., Alonso-Coello, P., & Schünemann, H. J. (2008). GRADE: an emerging consensus on rating quality of evidence and strength of recommendations. *BMJ*, *336*(7650), 924-926. https://doi.org/10.1136/bmj.39489.470347.ad

*Haim-Nachum, S., Amsalem, D., Lazarov, A., Zabag, R., Neria, Y., & Sopp, M. R. (2024). Anhedonia mediates the relationships between childhood trauma and symptom severity of PTSD and depression, but not of social anxiety. *Journal of Affective Disorders*, *344*, 577-584. https://doi.org/https://doi.org/10.1016/j.jad.2023.10.107

*Haim-Nachum, S., Lazarov, A., Zabag, R., Martin, A., Bergman, M., Neria, Y., & Amsalem, D. (2024). Self-stigma mediates the relationships between childhood maltreatment and symptom levels of PTSD, depression, and anxiety. *Eur J Psychotraumatol*, *15*(1), 2370174. https://doi.org/10.1080/20008066.2024.2370174

*Haj-Yahia, M. M., Sokar, S., Hassan-Abbas, N., & Malka, M. (2019). The relationship between exposure to family violence in childhood and post-traumatic stress symptoms in young adulthood: The mediating role of social support. *Child Abuse & Neglect*, *92*, 126-138. https://doi.org/10.1016/j.chiabu.2019.03.023

Hariton, E., & Locascio, J. J. (2018). Randomised controlled trials - the gold standard for effectiveness research: Study design: randomised controlled trials. *Bjog*, *125*(13), 1716. https://doi.org/10.1111/1471-0528.15199

Harris, J., Loth, E., & Sethna, V. (2024). Tracing the paths: a systematic review of mediators of complex trauma and complex post-traumatic stress disorder. *Front Psychiatry*, *15*, 1331256. https://doi.org/10.3389/fpsyt.2024.1331256

Harvey, A. G., Bryant, R. A., & Tarrier, N. (2003). Cognitive behaviour therapy for posttraumatic stress disorder. *Clinical psychology review*, *23*(3), 501-522. https://doi.org/https://doi.org/10.1016/S0272-7358(03)00035-7

Harvey, R., Charokopou, M., Arteaga Duarte, C., & Kiri, S. (2020). PNS22 Network Meta-Analysis: A Comparison of Bayesian and Frequentist Approaches to Synthesis, and Evaluation of Their Suitability for Decision-Making. *Value in Health*, *23*, S647. https://doi.org/10.1016/j.jval.2020.08.1466

Hayes, A. F. (2009). Beyond Baron and Kenny: Statistical mediation analysis in the new millennium. *Communication Monographs*, *76*(4), 408-420. https://doi.org/10.1080/03637750903310360

Hayes, A. F., & Preacher, K. J. (2010). Quantifying and Testing Indirect Effects in Simple Mediation Models When the Constituent Paths Are Nonlinear. *Multivariate Behav Res*, *45*(4), 627-660. https://doi.org/10.1080/00273171.2010.498290

Hernandez-Velasco, L. L., Abanto-Valle, C. A., Dey, D. K., & Castro, L. M. (2023). A Bayesian approach for mixed effects state-space models under skewness and heavy tails. *Biom J*, *65*(8), e2100302. https://doi.org/10.1002/bimj.202100302

*Hosseini Ramaghani, N. A., Rezaei, F., Sepahvandi, M. A., Gholamrezaei, S., & Mirderikvand, F. (2019). The mediating role of the metacognition, time perspectives and experiential avoidance on the relationship between childhood trauma and post-traumatic stress disorder symptoms. *European Journal of Psychotraumatology*, *10*(1). https://doi.org/10.1080/20008198.2019.1648173

Hou, H., Zhang, C., Tang, J., Wang, J., Xu, J., Zhou, Q., Yan, W., Gao, X., & Wang, W. (2022). Childhood Experiences and Psychological Distress: Can Benevolent Childhood Experiences Counteract the Negative Effects of Adverse Childhood Experiences? *Front Psychol*, *13*, 800871. https://doi.org/10.3389/fpsyg.2022.800871

*Huang, Y. L., Fonagy, P., Feigenbaum, J., Montague, P. R., & Nolte, T. (2020). Multidirectional pathways between attachment, mentalizing, and posttraumatic stress symptomatology in the context of childhood trauma. *Psychopathology*, *53*(1), 48-58. https://doi.org/10.1159/000506406

Hyland, P., Shevlin, M., & Brewin, C. R. (2023). The memory and identity theory of ICD-11 complex posttraumatic stress disorder. *Psychological Review*, *130*(4), 1044-1065. https://doi.org/10.1037/rev0000418

Hyland, P., Shevlin, M., Brewin, C. R., Cloitre, M., Downes, A. J., Jumbe, S., Karatzias, T., Bisson, J. I., & Roberts, N. P. (2017). Validation of post-traumatic stress disorder (PTSD) and complex PTSD using the International Trauma Questionnaire. *Acta Psychiatrica Scandinavica*, *136*(3), 313-322. https://doi.org/10.1111/acps.12771

*Ironson, G., Fitch, C., Banerjee, N., Hylton, E., Ivardic, I., Safren, S. A., & O'Cleirigh, C. (2019). Posttraumatic cognitions, childhood sexual abuse characteristics, and posttraumatic stress disorder in men who have sex with men. *Child Abuse & Neglect*, *98*. https://doi.org/10.1016/j.chiabu.2019.104187

*Jernslett, M., Anastassiou-Hadjicharalambous, X., Lioupi, C., Syros, I., Kapatais, A., Karamanoli, V., Evgeniou, E., Messas, K., Palaiokosta, T., Papathanasiou, E., & Lotzin, A. (2022). Disentangling the associations between past childhood adversity and psychopathology during the COVID-19 pandemic: The mediating roles of specific pandemic stressors and coping strategies. *Child Abuse & Neglect*, *129*, 1-12. https://doi.org/10.1016/j.chiabu.2022.105673

Juárez, M. A., & Steel, M. F. J. (2010). Non-Gaussian dynamic Bayesian modelling for panel data. *Journal of Applied Econometrics*, *25*(7), 1128-1154. https://doi.org/https://doi.org/10.1002/jae.1113

Kar, N. (2011). Cognitive behavioral therapy for the treatment of post-traumatic stress disorder: a review. *Neuropsychiatr Dis Treat*, *7*, 167-181. https://doi.org/10.2147/ndt.S10389

Karatzias, T., Mc Glanaghy, E., & Cloitre, M. (2023). Enhanced Skills Training in Affective and Interpersonal Regulation (ESTAIR): A New Modular Treatment for ICD-11 Complex Posttraumatic Stress Disorder (CPTSD). *Brain Sci*, *13*(9). https://doi.org/10.3390/brainsci13091300

Karatzias, T., Murphy, P., Cloitre, M., Bisson, J., Roberts, N., Shevlin, M., Hyland, P., Maercker, A., Ben-Ezra, M., Coventry, P., Mason-Roberts, S., Bradley, A., & Hutton, P. (2019). Psychological interventions for ICD-11 complex PTSD symptoms: systematic review and meta-analysis. *Psychological Medicine*, *49*(11), 1761-1775. https://doi.org/10.1017/S0033291719000436

Karatzias, T., Shevlin, M., Cloitre, M., Busuttil, W., Graham, K., Hendrikx, L., Hyland, P., Biscoe, N., & Murphy, D. (2024). Enhanced Skills Training in Affective and Interpersonal Regulation versus Treatment as Usual for ICD-11 Complex PTSD: A Pilot Randomised Controlled Trial (The RESTORE Trial). *Psychotherapy and Psychosomatics*, *93*(3), 203-215. https://doi.org/10.1159/000538428

Karatzias, T., Shevlin, M., Fyvie, C., Grandison, G., Garozi, M., Latham, E., Sinclair, M., Ho, G. W. K., McAnee, G., Ford, J. D., & Hyland, P. (2020). Adverse and benevolent childhood experiences in Posttraumatic Stress Disorder (PTSD) and Complex PTSD (CPTSD): implications for trauma-focused therapies. *European Journal of Psychotraumatology*, *11*(1), 1793599. https://doi.org/10.1080/20008198.2020.1793599

Karatzias, T., Shevlin, M., Hyland, P., Brewin, C. R., Cloitre, M., Bradley, A., Kitchiner, N. J., Jumbe, S., Bisson, J. I., & Roberts, N. P. (2018). The role of negative cognitions, emotion regulation strategies, and attachment style in complex post-traumatic stress disorder: Implications for new and existing therapies. *British Journal of Clinical Psychology*, *57*(2), 177-185. https://doi.org/10.1111/bjc.12172

Kenny, D. A., Kashy, D. A., & Bolger, N. (1998). Data analysis in social psychology. In *The handbook of social psychology, Vols. 1-2, 4th ed.* (pp. 233-265). McGraw-Hill.

*Kiefer, R., Goncharenko, S., Forkus, S. R., Contractor, A. A., LeBlanc, N., & Weiss, N. H. (2022). Role of positive emotion regulation strategies in the association between childhood trauma and posttraumatic stress disorder among trauma-exposed individuals who use substances. *Anxiety, Stress & Coping: An International Journal*. https://doi.org/10.1080/10615806.2022.2079636

*Kim, H., Kim, S. A., & Kong, S. (2017). Resilience mediates impact of some childhood maltreatment on post‐traumatic stress symptoms in conscripted Marines in the Republic of Korea. *Research in Nursing & Health*, *40*(1), 51-62. https://doi.org/10.1002/nur.21773

*Kim, M., Hong, G., Kim, R. Y., Song, Y., Lee, H., Joo, Y., Kim, J., & Yoon, S. (2021). Severity of post-traumatic stress disorder and childhood abuse in adult crime victims as mediated by low resilience and dysfunctional coping strategies. *Child Abuse & Neglect*, *118*. https://doi.org/10.1016/j.chiabu.2021.105154

*Kitano, M., van der Does, F. H. S., Saito, T., Giltay, E. J., Chiba, T., Edo, N., Waki, F., Tachimori, H., Koga, M., van der Wee, N. J., Vermetten, E., & Nagamine, M. (2024). Self-compassion as a protective factor against post-traumatic stress symptoms induced by adverse childhood experiences: A cross-sectional study among Japan air self-defense force new recruits. *J Psychiatr Res*, *180*, 204-212. https://doi.org/10.1016/j.jpsychires.2024.10.010

*Knefel, M., Lueger-Schuster, B., Karatzias, T., Shevlin, M., & Hyland, P. (2019). From child maltreatment to ICD-11 complex post‐traumatic stress symptoms: The role of emotion regulation and re‐victimisation. *Journal of Clinical Psychology*, *75*(3), 392-403. https://doi.org/10.1002/jclp.22655

Kraemer, H.C. (2010). Moderators and Mediators: The MacArthur Updated View. In: Steptoe, A. (eds) *Handbook of Behavioral Medicine*. Springer, New York, NY. https://doi.org/10.1007/978-0-387-09488-5_55

*Kratzer, L., Heinz, P., Pfitzer, F., Padberg, F., Jobst, A., & Schennach, R. (2018). Mindfulness and pathological dissociation fully mediate the association of childhood abuse and PTSD symptomatology. *European Journal of Trauma & Dissociation*, *2*(1), 5-10. https://doi.org/https://doi.org/10.1016/j.ejtd.2017.06.004

*Lagdon, S., Ross, J., Robinson, M., Contractor, A. A., Charak, R., & Armour, C. (2021). Assessing the mediating role of social support in childhood maltreatment and psychopathology among college students in Northern Ireland. *Journal of Interpersonal Violence*, *36*(3-4), NP2112-NP2136. https://doi.org/10.1177/0886260518755489

Lapointe-Shaw, L., Bouck, Z., Howell, N. A., Lange, T., Orchanian-Cheff, A., Austin, P. C., Ivers, N. M., Redelmeier, D. A., & Bell, C. M. (2018). Mediation analysis with a time-to-event outcome: a review of use and reporting in healthcare research. *BMC Med Res Methodol*, *18*(1), 118. https://doi.org/10.1186/s12874-018-0578-7

LeardMann, C. A., Smith, B., & Ryan, M. A. K. (2010). Do adverse childhood experiences increase the risk of postdeployment posttraumatic stress disorder in US Marines? *BMC Public Health*, *10*(1), 437. https://doi.org/10.1186/1471-2458-10-437

Lee, H., & Schafer, M. (2021). Are Positive Childhood Experiences Linked to Better Cognitive Functioning in Later Life?: Examining the Role of Life Course Pathways. *J Aging Health*, *33*(3-4), 217-226. https://doi.org/10.1177/0898264320972547

Lemoine, N. (2019). Moving beyond noninformative priors: why and how to choose weakly informative priors in Bayesian analyses. *Oikos*, *128*, 912-928. https://doi.org/10.1111/oik.05985

Leo, D., Izadikhah, Z., Fein, E. C., & Forooshani, S. A. (2021). The Effect of Trauma on Religious Beliefs: A Structured Literature Review and Meta-Analysis. *Trauma Violence Abuse*, *22*(1), 161-175. https://doi.org/10.1177/1524838019834076

*Li, Y., & Liang, Y. (2023). The effect of childhood trauma on complex posttraumatic stress disorder: the role of self-esteem. *European Journal of Psychotraumatology*, *14*(2). https://doi.org/10.1080/20008066.2023.2272478

*Lilly, M. M., London, M. J., & Bridgett, D. J. (2014). Using SEM to examine emotion regulation and revictimization in predicting PTSD symptoms among childhood abuse survivors. *Psychological trauma: theory, research, practice, and policy*, *6*(6), 644-651. https://doi.org/10.1037/a0036460

Liu, S.-H., Ulbricht, C. M., Chrysanthopoulou, S. A., & Lapane, K. L. (2016). Implementation and reporting of causal mediation analysis in 2015: a systematic review in epidemiological studies. *BMC Research Notes*, *9*(1), 354. https://doi.org/10.1186/s13104-016-2163-7

Mackinnon, D. P., Lockwood, C. M., & Williams, J. (2004). Confidence Limits for the Indirect Effect: Distribution of the Product and Resampling Methods. *Multivariate Behav Res*, *39*(1), 99. https://doi.org/10.1207/s15327906mbr3901_4

*Maercker, A., Bernays, F., Rohner, S., & Thoma, M. (2022). A cascade model of complex posttraumatic stress disorder centered on childhood trauma and maltreatment, attachment, and socio-interpersonal factors. *Journal of Traumatic Stress*, *35*(2), 446-460. https://doi.org/https://doi.org/10.1002/jts.22756

Maxwell, S. E., Cole, D. A., & Mitchell, M. A. (2011). Bias in Cross-Sectional Analyses of Longitudinal Mediation: Partial and Complete Mediation Under an Autoregressive Model*. Multivariate Behavioral Research*, 46(5), 816–841. https://doi.org/10.1080/00273171.2011.606716

*McCormick, W. H., Carroll, T. D., Sims, B. M., & Currier, J. (2017). Adverse childhood experiences, religious/spiritual struggles, and mental health symptoms: Examination of mediation models. *Mental Health, Religion & Culture*, *20*(10), 1042-1054. https://doi.org/10.1080/13674676.2018.1440544

*McCuaig Edge, H. J., & Lee, J. E. C. (2020). The mediating role of alexithymia in the association between adverse childhood experiences and postdeployment mental health in Canadian Armed Forces Personnel. *Journal of Traumatic Stress*, *33*(6), 1029-1038. https://doi.org/10.1002/jts.22547

*McLafferty, M., Ross, J., Waterhouse-Bradley, B., & Armour, C. (2019). Childhood adversities and psychopathology among military veterans in the US: The mediating role of social networks. *Journal of Anxiety Disorders*, *65*, 47-55. https://doi.org/10.1016/j.janxdis.2019.05.001

*Menke, R. A., Morelen, D., Simon, V. A., Rosenblum, K. L., & Muzik, M. (2018). Longitudinal Relations Between Childhood Maltreatment, Maltreatment-Specific Shame, and Postpartum Psychopathology. *Child Maltreatment*, *23*(1), 44-53. https://doi.org/10.1177/1077559517720070

Merrick, M. T., Ports, K. A., Ford, D. C., Afifi, T. O., Gershoff, E. T., & Grogan-Kaylor, A. (2017). Unpacking the impact of adverse childhood experiences on adult mental health. *Child Abuse & Neglect*, *69*, 10-19. https://doi.org/https://doi.org/10.1016/j.chiabu.2017.03.016

Miller, M. W. (2003). Personality and the etiology and expression of PTSD: A three-factor model perspective. *Clinical Psychology: Science and Practice*, *10*(4), 373-393. https://doi.org/10.1093/clipsy.bpg040

*Mitchell, R., Hanna, D., Brennan, K., Curran, D., McDermott, B., Ryan, M., Craig, K., McCullough, E., Wallace, P., & Dyer, K. F. W. (2020). Alienation appraisals mediate the relationships between childhood trauma and multiple markers of posttraumatic stress. *Journal of Child & Adolescent Trauma*, *13*(1), 11-19. https://doi.org/10.1007/s40653-018-0220-1

Moher, D., Liberati, A., Tetzlaff, J., & Altman, D. G. (2009). Preferred Reporting Items for Systematic Reviews and Meta-Analyses: The PRISMA Statement. *PLoS Medicine*, *6*(7), e1000097. https://doi.org/10.1371/journal.pmed.1000097

Monnat, S. M., & Chandler, R. F. (2015). Long-Term Physical Health Consequences of Adverse Childhood Experiences. *The Sociological Quarterly*, *56*(4), 723-752. https://doi.org/https://doi.org/10.1111/tsq.12107

*Murphy, S. M., Shevlin, M., Armour, C., Elklit, A., & Christoffersen, M. N. (2014). Childhood adversity and PTSD experiences: testing a multiple mediator model. *Traumatology*, *20*(3), 225-231. https://doi.org/https://doi.org/10.1037/h0099838

Narayan, A. J., Merrick, J. S., Lane, A. S., & Larson, M. D. (2023). A multisystem, dimensional interplay of assets versus adversities: Revised benevolent childhood experiences (BCEs) in the context of childhood maltreatment, threat, and deprivation. *Development and Psychopathology*, 1-20. https://doi.org/10.1017/S0954579423000536

Narayan, A. J., Rivera, L. M., Bernstein, R. E., Harris, W. W., & Lieberman, A. F. (2018). Positive childhood experiences predict less psychopathology and stress in pregnant women with childhood adversity: A pilot study of the benevolent childhood experiences (BCEs) scale. *Child Abuse Negl*, *78*, 19-30. https://doi.org/10.1016/j.chiabu.2017.09.022

Nazarov, A., Frewen, P., Parlar, M., Oremus, C., MacQueen, G., McKinnon, M., & Lanius, R. (2014). Theory of mind performance in women with posttraumatic stress disorder related to childhood abuse. *Acta Psychiatrica Scandinavica*, *129*(3), 193-201. https://doi.org/https://doi.org/10.1111/acps.12142

Oberpriller, J., de Souza Leite, M., & Pichler, M. (2022). Fixed or random? On the reliability of mixed-effects models for a small number of levels in grouping variables. *Ecology and Evolution*, *12*(7), e9062. https://doi.org/https://doi.org/10.1002/ece3.9062

Ogle, C. M., Rubin, D. C., & Siegler, I. C. (2015). The relation between insecure attachment and posttraumatic stress: Early life versus adulthood traumas. *Psychol Trauma*, *7*(4), 324-332. https://doi.org/10.1037/tra0000015

Omidbakhsh, Z., Mohammadi, Z., & Soltanabadi, S. (2025). Childhood Maltreatment and Complex PTSD: A Systematic Literature Review. *Trauma Violence Abuse*, 15248380251320985. https://doi.org/10.1177/15248380251320985

*Özdemir, M., & Yazici, H. (2025). The Relationship Between Child Maltreatment, Posttraumatic Stress Disorder Symptoms, and Posttraumatic Growth: Mediating Role of Insecure Attachment. *J Interpers Violence*, 8862605251347645. https://doi.org/10.1177/08862605251347645

Preacher, K. J., & Hayes, A. F. (2008a). Asymptotic and resampling strategies for assessing and comparing indirect effects in multiple mediator models. *Behavior Research Methods*, *40*(3), 879-891. https://doi.org/10.3758/BRM.40.3.879

Preacher, K. J., & Hayes, A. F. (2008b). *Contemporary approaches to assessing mediation in communication research* [doi:10.4135/9781452272054.n2]. Sage Publications, Inc.

Preacher, K. J., & Kelley, K. (2011). Effect size measures for mediation models: Quantitative strategies for communicating indirect effects. *Psychological Methods*, *16*(2), 93-115. https://doi.org/10.1037/a0022658

Rizzo, R. R. N., Cashin, A. G., Bagg, M. K., Gustin, S. M., Lee, H., & McAuley, J. H. (2022). A Systematic Review of the Reporting Quality of Observational Studies That Use Mediation Analyses. *Prevention Science*, *23*(6), 1041-1052. https://doi.org/10.1007/s11121-022-01349-5

Röver, C. (2020). Bayesian Random-Effects Meta-Analysis Using the bayesmeta R Package. *Journal of Statistical Software*, *93*(6), 1 - 51. https://doi.org/10.18637/jss.v093.i06

*Russo, J. E., Dhruve, D. M., & Oliveros, A. D. (2023). Childhood trauma and ptsd symptoms: Disentangling the roles of emotion regulation and distress tolerance. *Research on Child and Adolescent Psychopathology*. https://doi.org/10.1007/s10802-023-01048-x

Ryan, R., & Hill, S. (2016). How to GRADE the quality of the evidence. *Cochrane Consumers and Communication Group*, *3.0*. <http://cccrg.cochrane.org/author-resources>.

Salazar Kämpf, M., Adam, L., Rohr, M. K., Exner, C., & Wieck, C. (2023). A Meta-Analysis of the Relationship Between Emotion Regulation and Social Affect and Cognition. *Clinical Psychological Science*, *11*(6), 1159-1189. https://doi.org/10.1177/21677026221149953

Schalinski, I., Teicher, M. H., Nischk, D., Hinderer, E., Müller, O., & Rockstroh, B. (2016). Type and timing of adverse childhood experiences differentially affect severity of PTSD, dissociative and depressive symptoms in adult inpatients. *BMC Psychiatry*, *16*(1). https://doi.org/10.1186/s12888-016-1004-5

*Schumm, J. A., Hob, S. E., & Keogh, N. J. (2004). Revictimization and Interpersonal Resource Loss Predicts PTSD Among Women in Substance-Use Treatment. *Journal of Traumatic Stress*, *17*(2), 173-181. https://doi.org/10.1023/b

Sege, R. D., & Harper Browne, C. (2017). Responding to ACEs With HOPE: Health Outcomes From Positive Experiences. *Academic Pediatrics*, *17*(7, Supplement), S79-S85. https://doi.org/https://doi.org/10.1016/j.acap.2017.03.007

*Seitz, K. I., Bertsch, K., & Herpertz, S. C. (2021). A prospective study of mental health during the covid‐19 pandemic in childhood trauma–exposed individuals: Social support matters. *Journal of Traumatic Stress*. https://doi.org/10.1002/jts.22660

Semochkina, D., & Walsh, C. D. (2025). Incorporating Additional Evidence as Prior Information to Resolve Non-Identifiability in Bayesian Disease Model Calibration: A Tutorial. *Stat Med*, *44*(6), e70039. https://doi.org/10.1002/sim.70039

*Simonelli, A., & Sacchi, C. (2016). *Childhood Traumatic Experiences and Post-Traumatic Stress Disorder in Female Adults: Which is the Role Played by Romantic Attachment?* https://doi.org/10.5772/65367

*Sistad, R. E., Simons, R. M., Mojallal, M., & Simons, J. S. (2021). The indirect effect from childhood maltreatment to PTSD symptoms via thought suppression and cognitive reappraisal. *Child Abuse & Neglect*, *114*. https://doi.org/10.1016/j.chiabu.2021.104939

Stein, D. J., Koenen, K. C., Friedman, M. J., Hill, E., McLaughlin, K. A., Petukhova, M., Ruscio, A. M., Shahly, V., Spiegel, D., Borges, G., Bunting, B., Caldas-de-Almeida, J. M., de Girolamo, G., Demyttenaere, K., Florescu, S., Haro, J. M., Karam, E. G., Kovess-Masfety, V., Lee, S., . . . Kessler, R. C. (2013). Dissociation in posttraumatic stress disorder: evidence from the world mental health surveys. *Biol Psychiatry*, *73*(4), 302-312. https://doi.org/10.1016/j.biopsych.2012.08.022

*Stevens, N. R., Gerhart, J., Goldsmith, R. E., Heath, N. M., Chesney, S. A., & Hobfoll, S. E. (2013). Emotion Regulation Difficulties, Low Social Support, and Interpersonal Violence Mediate the Link Between Childhood Abuse and Posttraumatic Stress Symptoms. *Behavior Therapy*, *44*(1), 152-161. https://doi.org/10.1016/j.beth.2012.09.003

*Street, A. E., Gibson, L. E., & Holohan, D. R. (2005). Impact of childhood traumatic events, trauma-related guilt, and avoidant coping strategies on PTSD symptoms in female survivors of domestic violence. *Journal of Traumatic Stress*, *18*(3), 245-252. https://doi.org/https://doi.org/10.1002/jts.20026

Sullivan, G. M., & Feinn, R. (2012). Using Effect Size-or Why the P Value Is Not Enough. *J Grad Med Educ*, *4*(3), 279-282. [https://doi.org/10.4300/jgme-d-12-00156.1](https://protect.checkpoint.com/v2/r02/___https:/doi.org/10.4300/jgme-d-12-00156.1___.YzJlOnVsc3RlcnVuaXZlcnNpdHk6YzpvOjI2MjBlNGVlYjlmYjExNzIyN2Y0MzA1ZWRmMzBkZTIwOjc6NzI0Nzo0NzE5ZGU1YmQ0NWRmYzI5Yzk5MGY0YmI1YzRlNThjNGRmN2JiMTlhNjRhZGU1YTJjYTg2M2M4OTNhNTQ5ZjJmOnA6VDpO)

Uzer, T., Beşiroğlu, L., & Karakılıç, M. (2020). Event centrality, transitional impact and symptoms of posttraumatic stress in a clinical sample*. Anxiety, stress, and coping*, 33(1), 75–88. https://doi.org/10.1080/10615806.2019.1695252

*van Dijke, A., Ford, J. D., Frank, L. E., & van der Hart, O. (2015). Association of childhood complex trauma and dissociation with complex posttraumatic stress disorder symptoms in adulthood. *Journal of Trauma & Dissociation*, *16*(4), 428-441. https://doi.org/10.1080/15299732.2015.1016253

*van Dijke, A., Hopman, J. A. B., & Ford, J. D. (2018). Affect dysregulation, psychoform dissociation, and adult relational fears mediate the relationship between childhood trauma and complex posttraumatic stress disorder independent of the symptoms of borderline personality disorder. *European Journal of Psychotraumatology*, *9*(1). https://doi.org/10.1080/20008198.2017.1400878

*Vang, M. L., Shevlin, M., Karatzias, T., Fyvie, C., & Hyland, P. (2018). Dissociation fully mediates the relationship between childhood sexual and emotional abuse and DSM-5 PTSD in a sample of treatment-seeking adults. *European Journal of Trauma & Dissociation*, *2*(4), 173-178. https://doi.org/10.1016/j.ejtd.2018.02.004

*Vasilopoulou, E., Karatzias, T., Hyland, P., Wallace, H., & Guzman, A. (2020). The mediating role of early maladaptive schemas in the relationship between childhood traumatic events and complex posttraumatic stress disorder symptoms in older adults (&gt;64 years). *Journal of Loss and Trauma*, *25*(2), 141-158. https://doi.org/10.1080/15325024.2019.1661598

*Vera Békés, Dominik Szabó, Erika Evelyn Lévay, Ella Salgó, & Unoka, Z. (2023). MORAL INJURY AND SHAME MEDIATE THE RELATIONSHIP BETWEEN CHILDHOOD TRAUMA AND BORDERLINE PERSONALITY DISORDER, PTSD, AND COMPLEX PTSD SYMPTOMS IN PSYCHIATRIC INPATIENTS. *Journal of Personality Disorders*, *37*(4), 406–423.

Viswanathan M, Patnode C, Berkman ND, Bass EB, Chang S, Hartling L, Murad HM, Treadwell JR, & RL, K. (2017). Assessing the Risk of Bias in Systematic Reviews of Health Care Interventions. Methods Guide for Comparative Effectiveness Reviews. (Prepared by the Scientific Resource Center under Contract No. 290-2012-0004-C). *AHRQ Publication No. 17(18)-EHC036-EF. Rockville, MD: Agency for Healthcare Research and Quality*, *Posted final reports are located on the Effective Health Care Program search page*. https://doi.org/DOI: <https://doi.org/10.23970/AHRQEPCMETHGUIDE2>.

Viswanathan, M., Ansari, M. T., Berkman, N. D., Chang, S., Hartling, L., McPheeters, M., Santaguida, P. L., Shamliyan, T., Singh, K., Tsertsvadze, A., & Treadwell, J. R. (2008). AHRQ Methods for Effective Health Care Assessing the Risk of Bias of Individual Studies in Systematic Reviews of Health Care Interventions. In *Methods Guide for Effectiveness and Comparative Effectiveness Reviews*. Agency for Healthcare Research and Quality (US).

Vo, T.-T., Superchi, C., Boutron, I., & Vansteelandt, S. (2020). The conduct and reporting of mediation analysis in recently published randomized controlled trials: results from a methodological systematic review. *Journal of Clinical Epidemiology*, *117*, 78-88. https://doi.org/https://doi.org/10.1016/j.jclinepi.2019.10.001

Vo, T.-T., & Vansteelandt, S. (2022). Challenges in Systematic Reviews and Meta-Analyses of Mediation Analyses. *American Journal of Epidemiology*, *191*(6), 1098-1106. https://doi.org/10.1093/aje/kwac028

*Vranceanu, A.-M., Hobfoll, S. E., & Johnson, R. J. (2007). Child multi-type maltreatment and associated depression and PTSD symptoms: The role of social support and stress. *Child Abuse & Neglect*, *31*(1), 71-84. https://doi.org/10.1016/j.chiabu.2006.04.010

Wang, X., & Cheng, Z. (2020). Cross-Sectional Studies: Strengths, Weaknesses, and Recommendations. *Chest*, *158*(1s), S65-s71. https://doi.org/10.1016/j.chest.2020.03.012

*Watts, J., Kim, J., & Warwick, L. (2025). Posttraumatic Appraisals Mediate the Relationship Between Childhood Psychological Abuse and Complex PTSD Symptom Severity. *Journal of Counseling & Development*, *103*(3), 276-287. https://doi.org/https://doi.org/10.1002/jcad.12561

*Watts, J., Leeman, M., O'Sullivan, D., Castleberry, J., & Baniya, G. (2021). Childhood emotional maltreatment and post-traumatic stress disorder in the context of centrality of the event and intrusive rumination. *Rehabilitation Counseling Bulletin*, *64*(2), 108-117. https://doi.org/10.1177/0034355220925889

*Weiss, N. H., Tull, M. T., Lavender, J., & Gratz, K. L. (2013). Role of emotion dysregulation in the relationship between childhood abuse and probable PTSD in a sample of substance abusers. *Child Abuse & Neglect*, *37*(11), 944-954. https://doi.org/https://doi.org/10.1016/j.chiabu.2013.03.014

Williams, J., Bucci, S., Berry, K., & Varese, F. (2018). Psychological mediators of the association between childhood adversities and psychosis: A systematic review. *Clinical psychology review*, *65*, 175-196.

*Wilson, L. C., & Newins, A. R. (2018). The indirect effect of child maltreatment severity on adult PTSD symptoms through anxiety sensitivity. *Journal of Child Sexual Abuse: Research, Treatment, & Program Innovations for Victims, Survivors, & Offenders*, *27*(6), 682-698. https://doi.org/10.1080/10538712.2018.1488333

Wiseman, H., Hamilton-Giachritsis, C., & Hiller, R. M. (2021). The Relevance of Cognitive Behavioral Models of Post-Traumatic Stress Following Child Maltreatment: A Systematic Review. *Trauma, Violence, & Abuse*, *22*(1), 191-206. https://doi.org/10.1177/1524838019827894

Woodhouse, S., Brown, R., & Ayers, S. (2018). A social model of posttraumatic stress disorder: Interpersonal trauma, attachment, group identification, disclosure, social acknowledgement, and negative cognitions. *Journal of Theoretical Social Psychology*, *2*(2), 35-48. https://doi.org/https://doi.org/10.1002/jts5.17

Woodrow, A. (2021). *Chapter 2 –A systematic review of mediators of the pathway between childhood trauma and positive psychotic symptoms* [Doctoral Thesis]. Edinburgh Napier University.

World Health Organisation. (2018). International statistical classification of diseases and related health problems (11th revision) *3*.

*Zhang, H., Pittman, D. M., Lamis, D. A., Fischer, N. L., Schwenke, T. J., Carr, E. R., Shah, S., & Kaslow, N. J. (2015). Childhood maltreatment and PTSD: Spiritual well-being and intimate partner violence as mediators. *Journal of Aggression, Maltreatment & Trauma*, *24*(5), 501-519. https://doi.org/10.1080/10926771.2015.1029182

Zuj, D. V., Palmer, M. A., Lommen, M. J. J., & Felmingham, K. L. (2016). The centrality of fear extinction in linking risk factors to PTSD: A narrative review. *Neuroscience & Biobehavioral Reviews*, *69*, 15-35. https://doi.org/https://doi.org/10.1016/j.neubiorev.2016.07.014
